# Genome-wide association and population-tailored polygenic risk for Parkinson’s disease in Taiwan

**DOI:** 10.1038/s41531-026-01349-0

**Published:** 2026-05-22

**Authors:** Yung-Tsai Chu, Yu-An Su, Chin-Hsien Lin, Chun-Hwei Tai, Yih-Ru Wu, Chien-Tai Hong, Yu-Wei Chen, Mong-Hsun Tsai, John Hardy, Kin-Ying Mok, Ruey-Meei Wu, East Asian Parkinson Disease Genomics Consortium, Yung-Tsai Chu, Yu-An Su, Chin-Hsien Lin, Yih-Ru Wu, Mong-Hsun Tsai, Ruey-Meei Wu, Ji-Feng Guo, Bei-Sha Tang, Huifeng Shang, Chunyu Li, Huong Thi Thanh Nguyen, Thi Thanh Huyen Vu, Anh Tuan Nguyen, Nancy Y. Ip, Amy K. Y. Fu, Fanny C. F. Ip, Xiaopu Zhou, Han Cao, Kin Ying Mok, John Hardy, Germaine Hiu Fai Chan, Nelson Yuk-Fai Cheung, Wing-Chi Fong, Phillip Chan, Norlinah Mohamed Ibrahim, Shahrul Azmin, Nor Azian Abdul Murad, Siti Aishah Sulaiman, Siti Hajar Md Desa, Wael Mohamed, Sulev Koks, Abigail Pfaff, Lewis Singleton, Yun Joong Kim, Seok Jong Chung, Seong-Min Choi, Yu Chen, Yuewen Chen, Tao Ye, Hsiu-Chuan Wu, Tomotaka Shiraishi, Hidetomo Murakami, Renpei Sengoku, Nobutaka Hattori, Manabu Funayama, Tatsushi Toda, Wataru Satake, Kotaro Ogaki, Kenya Nishioka, Andrew B. Singleton, Mike A. Nalls, Cornelis Blauwendraat, Global Parkinson’s Genetics Program, Yung-Tsai Chu, Chin-Hsien Lin, Yih-Ru Wu, Ruey-Meei Wu, Xiaopu Zhou, Nelson Yuk-Fai Cheung, Norlinah Mohamed Ibrahim, Shahrul Azmin, Nor Azian Abdul Murad, Yun Joong Kim, Hsiu-Chuan Wu, Tomotaka Shiraishi, Nobutaka Hattori, Pin-Jui Kung, Serena Wu, Kai Shi Lim, Yi Wen Tay, Azlina Ahmad-Annuar, Tzi Shin Toh, Shen-Yang Lim, Ai Huey Tan, Jia Lun Lim, Lei-Cheng Lit, Anis Nadhirah Khairul Anuar, Hans Xing Ding, Elaine Guo Yan Chew, Jia Nee Foo, Eng-King Tan, Manabu Funayama, Hiroyo Yoshino, Rauan Kaiyrzhanov, Altynay Karimova, Gulnaz Kaishibayeva, Cholpon Shambetova, Mariam Isayan, Samson Khachatryan, Zaruhi Tavadyan, Han-Joon Kim, Jee-Young Lee, Sun Ju Chung, Beomseok Jeon, Beisha Tang, Huifang Shang, Jifeng Guo, Piu Chan, Wei Luo, Germaine Hiu-Fai Chan, Nancy Ip, Phillip Chan, Asha Kishore, K. P. Divya, Pramod Pal, Prashanth Lingappa Kukkle, Roopa Rajan, Rupam Borgohain, Mehri Salari, Bayasgalan Tserensodnom, Rajeev Ojha, Maria Leila Doquenia, Raymond Rosales, Duan Nguyen, Toan Nguyen, Maria Teresa Periñan, Paul Suhwan Lee, Mathew J. Koretsky, Sara Bandres-Ciga, Fulya Akçimen, Henry Houlden, Laurel Screven, Mie Rizig, Njideka Okubadejo, Emilia M. Gatto, Marcelo Kauffman, Claire E. Shepherd, Julie Hunter, Kishore Kumar, Melina Ellis, Miguel E. Rentería, Sulev Koks, Alexander Zimprich, Carlos Rieder, Vitor Tumas, Sarah Camargos, Edward A. Fon, Ted Fon, Oury Monchi, Benjamin Pizarro Galleguillos, Patricio Olguin, Marcelo Miranda, Maria Leonor Bustamante, Gonzalo Arboleda, Jorge Orozco, Marlene Jimenez del Rio, Alvaro Hernandez, Mohamed Salama, Walaa A. Kamel, Yared Z. Zewde, Alexis Brice, Jean-Christophe Corvol, Ana Westenberger, Christine Klein, Eva-Juliane Vollstedt, Harutyun Madoev, Joanne Trinh, Johanna Junker, Katja Lohmann, Lara M. Lange, Anastasia Illarionova, Brit Mollenhauer, Franziska Hopfner, Günter Höglinger, Manu Sharma, Thomas Gasser, Zih-Hua Fang, Sergio Groppa, Albert Akpalu, Efthymios Dardiotis, Georgia Xiromerisiou, Georgios Hadjigeorgiou, Ioannis Dagklis, Ioannis Tarnanas, Leonidas Stefanis, Maria Stamelou, Alex Medina, Andrea Quattrone, Enza Maria Valente, Micol Avenali, Lucilla Parnetti, Tommaso Schirinzi, Rejko Krüger, Wael Mohamed, Daniel Martinez-Ramirez, Mayela Rodriguez-Violante, Paula Reayes-Perez, Tim J. Anderson, Toni L. Pitcher, Oluwadamilola Ojo, Jan O. Aasly, Lasse Pihlstrøm, Manuela Tan, Shoaib Ur-Rehman, Mario Cornejo-Olivas, Angel Vinuela, Elena Iakovenko, Bashayer Al Mubarak, Muhammad Umair, Ferzana Amod, Jonathan Carr, Soraya Bardien, Esther Cubo, Ignacio Alvarez, Janet Hoenicka, Katrin Beyer, Pau Pastor, Sarah El-Sadig, Christiane Zweier, Paul Krack, Rim Amouri, Samia Ben Sassi, A. Nazl Başak, Özgür Öztop Çakmak, Sibel Ertan, Gencer Genc, Alejandro Martínez-Carrasco, Anette Schrag, Anthony Schapira, Eleanor J. Stafford, Huw Morris, John Hardy, Nicholas Wood, Olaitan Okunoye, Rimona Weil, Simona Jasaitye, Vida Obese, Camille Carroll, Claire Bale, Donald Grosset, Kin Y. Mok, Nigel Williams, Patrick A. Lewis, Seth Love, Simon Stott, Alberto Espay, Luca Marsili, Alyssa O’Grady, Bernadette Siddiqi, Bradford Casey, Brian Fiske, Charisse Comart, Justin C. Solle, Kaileigh Murphy, Maggie Kuhl, Naomi Louie, Sohini Chowdhury, Todd Sherer, Andrew K. Sobering, Cabell Jonas, Carlos Cruchaga, Caroline B. Pantazis, Claire Wegel, Deborah Hall, Ejaz Shamim, Jared Williamson, Ekemini Riley, Sonya Dumanis, Geidy E. Serrano, Thomas Beach, Honglei Chen, Ignacio Juan Keller Sarmiento, Niccolò E. Mencacci, Steven Lubbe, Joseph Jankovic, Miguel Inca-Martinez, Joshua Shulman, Karen Nuytemans, Karl Kieburtz, Katerina Markopoulou, Kenneth Marek, Lana M. Chahine, Lauren Ruffrage, Marissa Dean, Lisa Shulman, Roger Albin, Roy Alcalay, Ruth Walker, Tao Xie, Tatiana Foroud, Masharip Atadzhanov

**Affiliations:** 1 Center of Parkinson’s Disease and Movement Disorders, Department of Neurology, National Taiwan University Hospital, Taipei, Taiwan; 2 Genome and Systems Biology Degree Program, Academia Sinica and National Taiwan University, Taipei, Taiwan; 3 National Taiwan University College of Medicine Graduate Institute of Clinical Medicine, Taipei, Taiwan; 4 Department of Neurology, Linkou Chang Gung Memorial Hospital, Taoyuan, Taiwan; 5 College of Medicine, Chang Gung University, Taoyuan, Taiwan; 6 Taipei Medical University-Shuang Ho Hospital, Ministry of Health and Welfare, New Taipei, Taiwan; 7 Department of Neurology, Landseed International Hospital, Taoyuan, Taiwan; 8 Department of Life Sciences, National Central University, Taoyuan, Taiwan; 9 Institute of Biotechnology, National Taiwan University, Taipei, Taiwan; 10 Department of Neurodegenerative Disease, Queen Square Institute of Neurology, UCL, London, UK; 11 Division of Life Science, The Hong Kong University of Science and Technology, Hong Kong Special Administrative Region, Hong Kong, China; 12 Hong Kong Center for Neurodegenerative Diseases, Hong Kong Special Administrative Region, Hong Kong, China; 13 Health science and wellness research center, National Taiwan university, Taipei, Taiwan; 14 Department of Neurology, Xiangya Hospital, Central South University, Changsha, China; 15 Center for Medical Genetics, School of Life Sciences, Central South University, Changsha, China; 16 National Clinical Research Center for Geriatric Disorders, Central South University, Changsha, China; 17 Department of Neurology, Laboratory of Neurodegenerative Disorders, National Clinical Research Center for Geriatrics, West China Hospital of Sichuan University, Chengdu, China; 18 Dinh Tien Hoang Institute of Medicine; Physiology Department, Hanoi Medical University, Hanoi, Vietnam; 19 Scientific Research Department, National Geriatric Hospital, Hanoi, Vietnam; 20 Neurology Department, Neurosurgical Centre, Vietduc University Hospital, Hanoi, Vietnam; 21 Division of Neurology, Department of Medicine, Queen Elizabeth Hospital, Hong Kong, China; 22 Division of Neurology, Department of Medicine and Therapeutics, Queen Elizabeth Hospital, Hong Kong, China; 23 Department of Medicine, Faculty of Medicine, Universiti Kebangsaan Malaysia, Kuala Lumpur, Malaysia; 24 Department of Medicine, Universiti Kebangsaan Malaysia Medical Center, Bandar Tun Razak, Cheras, Kuala Lumpur, Malaysia; 25 UKM Medical Molecular Biology Institute (UMBI), Jalan Ya’acob Latiff, Bandar Tun Razak, Cheras, Kuala Lumpur, Malaysia; 26 Nursing Service Department, Chancellor Tuanku Muhriz Hospital, Universiti Kebangsaan Malaysia Medical Center, Bandar Tun Razak, Cheras, Kuala Lumpur, Malaysia; 27 Department of BMS, Medical School, International Islamic University Malaysia (IIUM), Bandar Indera Mahkota Campus, Jalan Sultan Ahmad Shah, Kuantan, Pahang Darul Makmur Malaysia; 28 Perron Institute for Neurological and Translational Science, Perth, WA Australia; 29 Centre for Molecular Medicine and Innovative Therapeutics, Murdoch University, Perth, WA Australia; 30 Department of Neurology, Center for Neurodegenerative Disorders, Yongin Severance Hospital, Yonsei University College of Medicine, Yonsei University, Seoul, South Korea; 31 Department of Neurology, Chonnam National University Medical School and Hospital, Chonnam National University, Gwangju, South Korea; 32 Chinese Academy of Sciences Key Laboratory of Brain Connectome and Manipulation, Shenzhen Key Laboratory of Translational Research for Brain Diseases, The Brain Cognition and Brain Disease Institute, Shenzhen Institute of Advanced Technology, Chinese Academy of Sciences; Shenzhen-Hong Kong Institute of Brain Science-Shenzhen Fundamental Research Institutions, Shenzhen, Guangdong China; 33 Department of Neurology, Jikei University School of Medicine, Tokyo, Japan; 34 Department of Neurology, Daisan Hospital, Jikei University School of Medicine, Tokyo, Japan; 35 Department of Neurology, Juntendo University School of Medicine, Tokyo, Japan; 36 Neurodegenerative Disorders Collaborative Laboratory, RIKEN Center for Brain Science, Saitama, Japan; 37 Research Institute for Diseases of Old Age, Graduate School of Medicine, Juntendo University, Tokyo, Japan; 38 Department of Neurology, Graduate School of Medicine, The University of Tokyo, Tokyo, Japan; 39 Department of Neurology, Juntendo University Urayasu Hospital, Chiba, Japan; 40 Global Parkinson’s Genetics Program (GP2), Bethesda, MD USA; 41 Data Tecnica, Bethesda, MD USA; 42 Aligning Science Across Parkinson’s (ASAP), Bethesda, MD USA; 43 National Taiwan University, Taipei City, Taiwan; 44 Department of Biomedical Science, Faculty of Medicine, Universiti Malaya, Kuala Lumpur, Malaysia; 45 Department of Medicine, Faculty of Medicine, Universiti Malaya, Kuala Lumpur, Malaysia; 46 The Mah Pooi Soo & Tan Chin Nam Centre for Parkinson’s & Related Disorders, Faculty of Medicine, Universiti Malaya, Kuala Lumpur, Malaysia; 47 Department of Physiology, Faculty of Medicine, Universiti Malaya, Kuala Lumpur, Malaysia; 48 Lee Kong Chian School of Medicine, Nanyang Technological University Singapore, Singapore, Singapore; 49 Duke-National University of Singapore Medical School, Singapore, Singapore; 50 Department of Neurology, National Neuroscience Institute, Singapore General Hospital, Singapore, Singapore; 51 International Collaborative Research Administration, Juntendo University, Tokyo, Japan; 52 South Kazakhstan Medical Academy, Department of Neurology, Shymkent, Kazakhstan; 53 Department of Neuromuscular Diseases, UCL Queen Square Institute of Neurology, University College London, London, UK; 54 Institute of Neurology and Neurorehabilitation, Almaty, Kazakhstan; 55 Kyrgyz State Medical Academy, Bishkek, Kyrgyzstan; 56 Department of Neurology and Neurosurgery, National Institute of Health, Yerevan, Armenia; 57 Somnus Neurology Clinic, Yerevan, Armenia; 58 Department of Neurology, Seoul National University Hospital, College of Medicine, Seoul National University, Seoul, Republic of Korea; 59 Department of Neurology, Seoul Metropolitan Government-Seoul National University Boramae Medical Center, Seoul National University College of Medicine, Seoul, Korea; 60 Department of Neurology, Asan Medical Center, University of Ulsan College of Medicine, Seoul, South Korea; 61 Central South University, Changsha, China; 62 Capital Medical University, Beijing, China; 63 Zhejiang University, Hangzhou, China; 64 Aster Medcity, Kochi, India; 65 Sree Chitra Tirunal Institute for Medical Sciences and Technology, Thiruvananthapuram, India; 66 National Institute of Mental Health & Neurosciences (NIMHANS), Bengaluru, India; 67 Manipal Hospital, Delhi, India; 68 All India Institute of Medical Sciences, Delhi, India; 69 Nizam’s Institute of Medical Sciences, Hyderabad, India; 70 Shahid Beheshti University of Medical Sciences, Tehran, Iran; 71 Mongolian National University of Medical Sciences, Ulaanbaatar, Mongolia; 72 Tribhuvan University, Kirtipur, Nepal; 73 Metropolitan Medical Center, Manila, Philippines; 74 Hue University, Huế, Vietnam; 75 Unidad de Trastornos del Movimiento, Servicio de Neurología y Neurofisiología Clínica, Instituto de Biomedicina de Sevilla, Hospital Universitario Virgen del Rocío, Consejo Superior de Investigaciones Científicas (CSIC), Universidad de Sevilla, Seville, Spain; 76 Centre for Preventive Neurology, Wolfson Institute of Population Health, Queen Mary University of London, London, UK; 77 Center for Alzheimer’s and Related Dementias, National Institute on Aging and National Institute of Neurological Disorders and Stroke, National Institutes of Health, Bethesda, Maryland USA; 78 Laboratory of Neurogenetics, National Institute on Aging, National Institutes of Health, Bethesda, MD USA; 79 Preventive Neurology Unit, Wolfson Institute of Population Health, Queen Mary University of London, London, UK; 80 Department of Medicine, College of Medicine, University of Lagos, Lagos, Nigeria; 81 Sanatorio de la Trinidad Mitre-INEBA, Buenos Aires, Argentina; 82 Hospital JM Ramos Mejia, Buenos Aires, Argentina; 83 Neuroscience Research Australia, Sydney, NSW Australia; 84 ANZAC Research Institute, Concord, NSW Australia; 85 Garvan Institute of Medical Research and Concord Repatriation General Hospital, Darlinghurst, NSW Australia; 86 Concord Hospital, Concord, NSW Australia; 87 QIMR Berghofer Medical Research Institute, Herston, QLD Australia; 88 Murdoch University, Perth, WA Australia; 89 Medical University of Vienna, Vienna, Austria; 90 Universidade Federal do Rio Grande do Sul / Hospital de Clínicas de Porto Alegre, Porto Alegre, Brazil; 91 University of São Paulo, São Paulo, Brazil; 92 Universidade Federal de Minas Gerais, Belo Horizonte, Brazil; 93 McGill University, Montreal, QC Canada; 94 Universidad de Chile, Santiago, Chile; 95 Fundación Diagnosis, Santiago, Chile; 96 Faculty of Medicine, Universidad de Chile, Santiago, Chile; 97 CETRAM, Santiago, Chile; 98 Universidad Nacional de Colombia, Bogotá, Colombia; 99 Fundación Valle del Lili, Santiago de Cali, Colombia; 100 University of Antioquia, Medellín, Colombia; 101 University of Costa Rica, San José, Costa Rica; 102 The American University in Cairo, Cairo, Egypt; 103 Beni-Suef University, Beni Suef, Egypt; 104 Addis Ababa University, Addis Ababa, Ethiopia; 105 Paris Brain Institute, Paris, France; 106 Sorbonne Université, Paris, France; 107 University of Lübeck, Lübeck, Germany; 108 University Medical Center Schleswig-Holstein, Lübeck, Germany; 109 Deutsches Zentrum für Neurodegenerative Erkrankungen (DZNE), Göttingen, Germany; 110 University Medical Center Göttingen, Göttingen, Germany; 111 University Hospital LMU Munich, Munich, Germany; 112 Hannover Medical School, Hannover, Germany; 113 University of Tübingen, Tübingen, Germany; 114 German Center for Neurodegenerative Diseases (DZNE), Tübingen, Germany; 115 University of Mainz, Mainz, Germany; 116 University of Ghana Medical School, Accra, Ghana; 117 University of Thessaly, Larissa, Greece; 118 Aristotle University of Thessaloniki, Thessaloniki, Greece; 119 Ionian University, Corfu, Greece; 120 Biomedical Research Foundation of the Academy of Athens, Athens, Greece; 121 Diagnostic and Therapeutic Centre HYGEIA Hospital, Marousi, Greece; 122 Hospital San Felipe, Tegucigalpa, Honduras; 123 Magna Græcia University of Catanzaro, Catanzaro, Italy; 124 University of Pavia, Pavia, Italy; 125 University of Perugia, Perugia, Italy; 126 University of Rome Tor Vergata, Rome, Italy; 127 Luxembourg Centre for Systems Biomedicine, University of Luxembourg, Belvaux, Luxembourg; 128 Neuroscience Unit, Clinical Pharmacology Dept, Menoufia Medical School, Shebeen El-Kom, Egypt; 129 Instituto Nacional de Neurología y Neurocirugía, Mexico City, Mexico; 130 Universidad Nacional Autónoma de México, Mexico City, Mexico; 131 University of Otago, Christchurch, New Zealand; 132 Norwegian University of Science and Technology, Trondheim, Norway; 133 Oslo University Hospital, Oslo, Norway; 134 University of Science and Technology Bannu, Khyber Pakhtunkhwa, Pakistan; 135 Instituto Nacional de Ciencias Neurológicas, Lima, Peru; 136 University of Puerto Rico, San Juan, PR USA; 137 Research Center of Neurology, Moscow, Russia; 138 King Faisal Specialist Hospital and Research Center, Riyadh, Saudi Arabia; 139 King Abdullah International Medical Research Center, Jeddah, Saudi Arabia; 140 University of KwaZulu-Natal, Durban, South Africa; 141 University of Stellenbosch, Stellenbosch, South Africa; 142 Hospital Universitario de Burgos, Burgos, Spain; 143 University Hospital Mutua Terrassa, Barcelona, Spain; 144 Institut de Recerca Sant Joan de Déu, Barcelona, Spain; 145 Research Institute Germans Trias i Pujol, Badalona, Spain; 146 University Hospital Germans Trias i Pujol, Badalona, Spain; 147 Faculty of Medicine, University of Khartoum, Khartoum, Sudan; 148 Inselspital, Bern University Hospital, University of Bern, Bern, Switzerland; 149 National Institute Mongi Ben Hamida of Neurology, Tunis, Tunisia; 150 Mongi Ben Hmida National Institute of Neurology, Tunis, Tunisia; 151 Koç University, Istanbul, Turkey; 152 Şişli Etfal Training and Research Hospital, Istanbul, Turkey; 153 University College London, London, UK; 154 HKUST Jockey Club Institute for Advanced Study, The Hong Kong University of Science and Technology, Clear Water Bay, Kowloon, Hong Kong, China; 155 University of Plymouth, Plymouth, UK; 156 Parkinson’s UK, London, UK; 157 University of Glasgow, Glasgow, UK; 158 Cardiff University, Cardiff, UK; 159 Royal Veterinary College, University of London, London, UK; 160 University of Bristol, Bristol, UK; 161 Cure Parkinson’s Trust, London, UK; 162 University of Cincinnati, Cincinnati, OH USA; 163 The Michael J. Fox Foundation for Parkinson’s Research, New York, NY USA; 164 Augusta University / University of Georgia Medical Partnership, Athens, GA USA; 165 Mid-Atlantic Permanente Medical Group, Rockville, MD USA; 166 Washington University in St. Louis, St. Louis, MO USA; 167 National Institutes of Health, Bethesda, MD USA; 168 Indiana University, Bloomington, IN USA; 169 Rush University Medical Center, Chicago, IL USA; 170 Kaiser Permanente, Oakland, CA USA; 171 Aligning Science Across Parkinson’s, Washington, DC, USA; 172 Banner Sun Health Research Institute, Sun City, AZ USA; 173 Michigan State University, East Lansing, MI USA; 174 Northwestern University, Chicago, IL USA; 175 Baylor College of Medicine, Houston, TX USA; 176 Baylor College of Medicine/Texas Children’s Hospital, Houston, TX USA; 177 University of Miami Miller School of Medicine, Miami, FL USA; 178 Beth Israel Deaconess Medical Center, Boston, MA USA; 179 NorthShore University HealthSystem, Evanston, IL USA; 180 Institute for Neurodegenerative Disorders, New Haven, CT USA; 181 University of Pittsburgh, Pittsburgh, PA USA; 182 University of Alabama at Birmingham, Birmingham, AL USA; 183 University of Maryland School of Medicine, Baltimore, MD USA; 184 University of Michigan, Ann Arbor, MI USA; 185 Columbia University Irving Medical Center, New York, NY USA; 186 James J. Peters VA Medical Center, Bronx, NY USA; 187 University of Chicago, Chicago, IL USA; 188 Indiana University School of Medicine, Indianapolis, IN USA; 189 University of Zambia, Lusaka, Zambia

**Keywords:** Diseases, Genetics, Neurology, Neuroscience

## Abstract

Genetic risk factors for Parkinson’s disease (PD) remain under-characterized in East Asian populations. We assembled a Taiwanese case–control cohort (2245 PD; 2147 controls), genotyped participants using the Illumina NeuroBooster Array, and imputed 7.6 million variants with the Taiwan Biobank reference panel. Logistic-regression GWAS identified genome-wide significant associations at *SNCA* and *MCCC1*, with the lead *SNCA* signal located in intron 4; conditional and joint analyses suggested an additional 5′ *SNCA* component. We observed suggestive associations at *GCH1*, *PPARGC1A*, and *GALNT13*. Locus-focused haplotype analyses refined the *SNCA* signal, delineating an East Asian–enriched risk haplotype, and confirmed risk effects of *LRRK2* p.G2385R and p.R1628P, including evidence consistent with a gene-dosage effect. A European-derived PRS showed modest discrimination in our cohort (AUC 0.59), while incorporating East Asian and Taiwan-relevant variants improved performance (AUC 0.62). Together, these results define the genetic architecture of PD in Taiwan, highlight shared and population-enriched risk components, and support ancestry-aware PRS construction for improved risk stratification.

## Introduction

Parkinson’s disease (PD) is the second most common neurodegenerative disorder worldwide.^1^ It is characterized clinically by tremor, rigidity, bradykinesia, and postural instability, and neuropathologically by selective dopaminergic neuron loss in the substantia nigra with Lewy bodies composed primarily of α-synuclein.

Although a minority of PD cases arise from rare, high-penetrance variants, most cases are polygenic and multifactorial. Common variants of modest effect collectively account for a meaningful portion of PD heritability; the largest European-ancestry meta-GWAS to date identified 90 independent risk signals explaining an estimated 16–36% of the heritable risk.^2^ Yet the field’s understanding of PD genetics remains disproportionately derived from European cohorts, with fewer large-scale studies in East Asian and Taiwanese populations.^3^

Both clinical features and genetic risk architectures show ancestry-related differences.^4^ Asian patients, for example, have lower reported rates of motor complications such as dyskinesia than European patients, and several PD risk alleles differ in frequency and effect size across populations. Notably, the *LRRK2* p.G2019S founder variant is enriched in European and certain West Asian groups, whereas *LRRK2* p.G2385R and p.R1628P are established risk alleles in individuals of East Asian ancestry.^5–7^ At *SNCA*, ancestry-specific patterns have also been described: association signals and local haplotype structure vary by population^8,9^, and distinct rs356182-centered haplotypes show different relationships to risk in Latino and European cohorts, highlighting locus complexity relevant to non-European populations as well. In *GBA1*, recent GWAS in African cohorts have likewise identified novel PD risk variants among individuals of African ancestry.^10^

These differences have direct implications for risk prediction^11^. Cross-ancestry evaluations consistently show that polygenic risk scores (PRS) trained on European data underperform in non-European populations, arguing for population-tailored models. In response to this imbalance, the East Asian Parkinson Disease Genomics Consortium (EAPDGC) was formed to coordinate multi-center efforts across the region.^12^ We undertook the first Taiwan-wide PD GWAS to (i) map PD risk loci and related haplotypes in a Taiwanese cohort, (ii) benchmark effect sizes against European and pan-East Asian studies, and (iii) construct a Taiwan-optimized PRS to assess improvements in predictive performance over European- and Asian-derived baselines.

## Results

### Cohort demographics

After quality control, the final cohort comprised **2251** individuals with Parkinson’s disease (PD) and **2268** controls (Supplementary Table S1 lists recruitment by site). Cases were slightly older at examination than controls (65.4 ± 11.2 vs. 63.5 ± 8.7 years) and more often male (51.5% vs. 44.4%). The mean age at PD onset was **60.5** ± **11.7** years. Key characteristics are summarized in Table 1. Age and sex were included as covariates in all association models. Population structure was evaluated by principal component analysis (PCA) using the 1000 Genomes Phase 3 reference panel; study participants clustered with the East Asian (EAS) superpopulation (CHB/CHS/JPT/KHV/CDX) (Supplementary Fig. S1). Case–control and site-level PCA were performed. We observed no obvious clustering or separation by disease status or by recruiting site, suggesting minimal population stratification and limited site-specific batch effects (Supplementary Fig. S2).

**Fig. 1:**
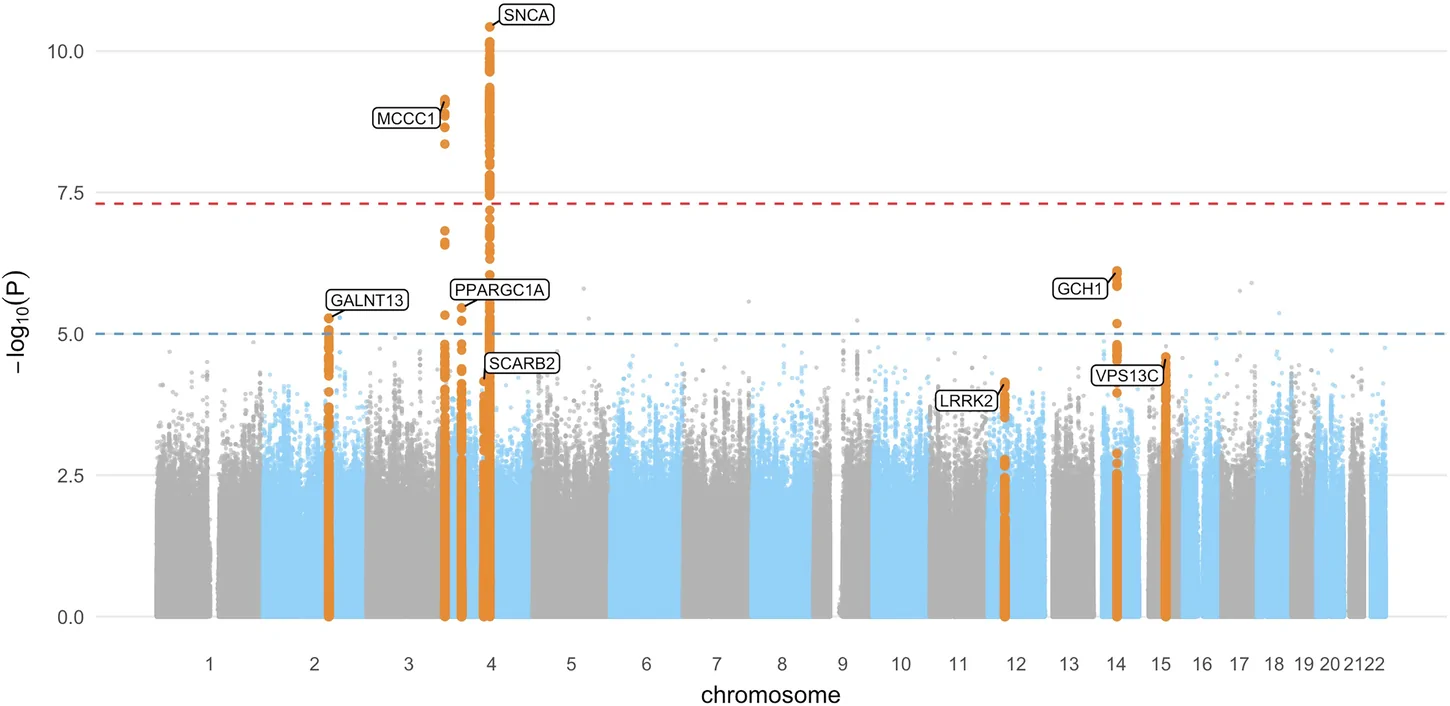
Genome-wide association results for Parkinson’s disease in the Taiwanese cohort. The Manhattan plot displays –log_10_p values for approximately 7.6 million variants after quality control and imputation. The red dashed line denotes the threshold for genome-wide significance (*p* = 5 × 10^–8^), and the blue dashed line indicates the threshold for suggestive significance (*p* = 1 × 10^–5^). Established PD risk loci, including *SNCA*, *MCCC1*, and *GCH1*, were successfully replicated. Additional signals in *PPARGC1A* and *GALNT13* emerged as potential novel associations specific to the Taiwanese population. Although not exceeding the suggestive threshold, loci in *VPS13C*, *LRRK2* (p.G2385R), and *SCARB2* also demonstrated supportive evidence consistent with prior reports, suggesting potential relevance in East Asian populations.

**Table 1:** Demographics of the study cohort after quality control

	PD case, *N* = 2251	Control, *N* = 2268
Male Sex	1184/ 2251 (51.5%)	1007 /2268 (44.4%)
Age at Exam	65.4 (11.2)	63.5 (8.7)
Age at onset	60.5 (11.7)	NA
Disease Duration	4.8 (4.4)	NA
*1* Mean (SD); n / N (%)		

Summary of age, sex, and case–control distribution of participants included in the genome-wide association study after quality control. The detailed number of participants contributed by each recruitment site is provided in Supplementary Table 1.

**Table 2:** Top genetic risk loci associated with PD in Taiwan

Chr	Position (hg38)	SNP (rsID)	Nearest Gene	Effect Allele	Other Allele	MAF	OR_CI	β	SE	*P*	Annotation
4	89759478	rs5860181	*SNCA*	A	ATGCATATT	0.43	0.75 (0.69–0.81)	–0.12	0.04	3.70e–11	Most significat in Taiwan
4	89765968	rs3775427	*SNCA*	G	A	0.43	0.75 (0.69–0.81)	–0.12	0.04	6.90e–11	Most significat in Taiwan
4	89761323	rs6826785	*SNCA*	T	C	0.43	0.76 (0.70–0.82)	–0.12	0.04	1.60e–10	Foo
3	183028420	rs34822404	*MCCC1*	C	T	0.43	1.31 (1.21–1.42)	0.12	0.04	7.20e–10	Most significat in Taiwan
3	183042285	rs10513789	*MCCC1*	T	G	0.43	1.3 (1.20–1.41)	0.12	0.04	1.40e–09	Nalls
4	89836121	rs3756059	*SNCA*	G	A	0.12	0.67(0.58- 0.76)	0.40	0.07	1.86e–09	Reported in the GWAS of RBD, located in 5’UTR
4	89704960	rs356182	*SNCA*	A	G	0.3	0.78 (0.71–0.86)	–0.11	0.05	1.90e–07	Nalls
14	54885803	rs3783640	*GCH1*	T	C	0.46	1.23 (1.14–1.33)	0.09	0.04	7.70e–07	Taiwan
14	54882151	rs11158026	*GCH1*	C	T	0.46	1.23 (1.14–1.33)	0.09	0.04	1.40e–06	Nalls
4	24009221	rs609501	*PPARGC1A*	A	G	0.48	1.22 (1.13–1.32)	0.09	0.04	3.50e–06	New
2	154088802	rs10186542	*GALNT13*	A	G	0.12	1.35 (1.18–1.55)	0.13	0.07	5.30e–06	New

**Table 3:** Replication of established Parkinson’s disease loci and targeted look-up in the Taiwanese cohort

Chr	Position (hg38)	rsID/variant	Gene	Effect Allele	Other Allele	MAF	OR	Beta	SE	*P*
15	61994009	rs12900645	*VPS13C*	G	A	0.352	0.82 (0.76–0.90)	–0.08	0.04	2.57E–05
4	76217199	rs76591264	*SCARB2/ FAM47E*	G	C	0.329	1.20 (1.10-1.32)	0.08	0.05	6.92E–05
12	40363526	rs34778348 (p.G2385R)	*LRRK2*	A	G	0.033	1.59 (1.25–2.01)	0.20	0.12	1.47E–04
4	76213633	rs11547135	*SCARB2/ FAM47E*	C	T	0.295	0.846 (0.772–0.927)	-0.17	0.05	3.55E–04
12	40320043	rs33949390 (p.R1628P)	*LRRK2*	C	G	0.029	1.49 (1.16–1.92)	0.17	0.13	1.94E–03

### Genome-wide association analysis (Fig. 1; Tables 2–3)

For the GWAS, after imputation and model-covariate completeness filters, **2245** PD cases and **2147** controls of Taiwanese ancestry remained, contributing ~**7.6 million** high-quality variants. The quantile–quantile plot showed minimal test-statistic inflation (λ_GC_ = **1.02**), indicating adequate control of population structure (Supplementary Fig. S2).

The Manhattan plot (Fig. 1) highlighted signals at ***SNCA*** and ***MCCC1***, with additional support at ***GCH1*** and several suggestive loci (*P* < 1×10^–5^). The most significant associations localized to established PD risk regions—*SNCA*, *MCCC1*, and *GCH1*. Beyond these, we observed suggestive associations at *PPARGC1A* and *GALNT13*, nominating potentially Taiwan-specific risk variants. Top results are summarized in Table 2 and Supplementary Table S2. Detailed regional association plots are provided in Supplementary Fig. S3.

**Fig. 2:**
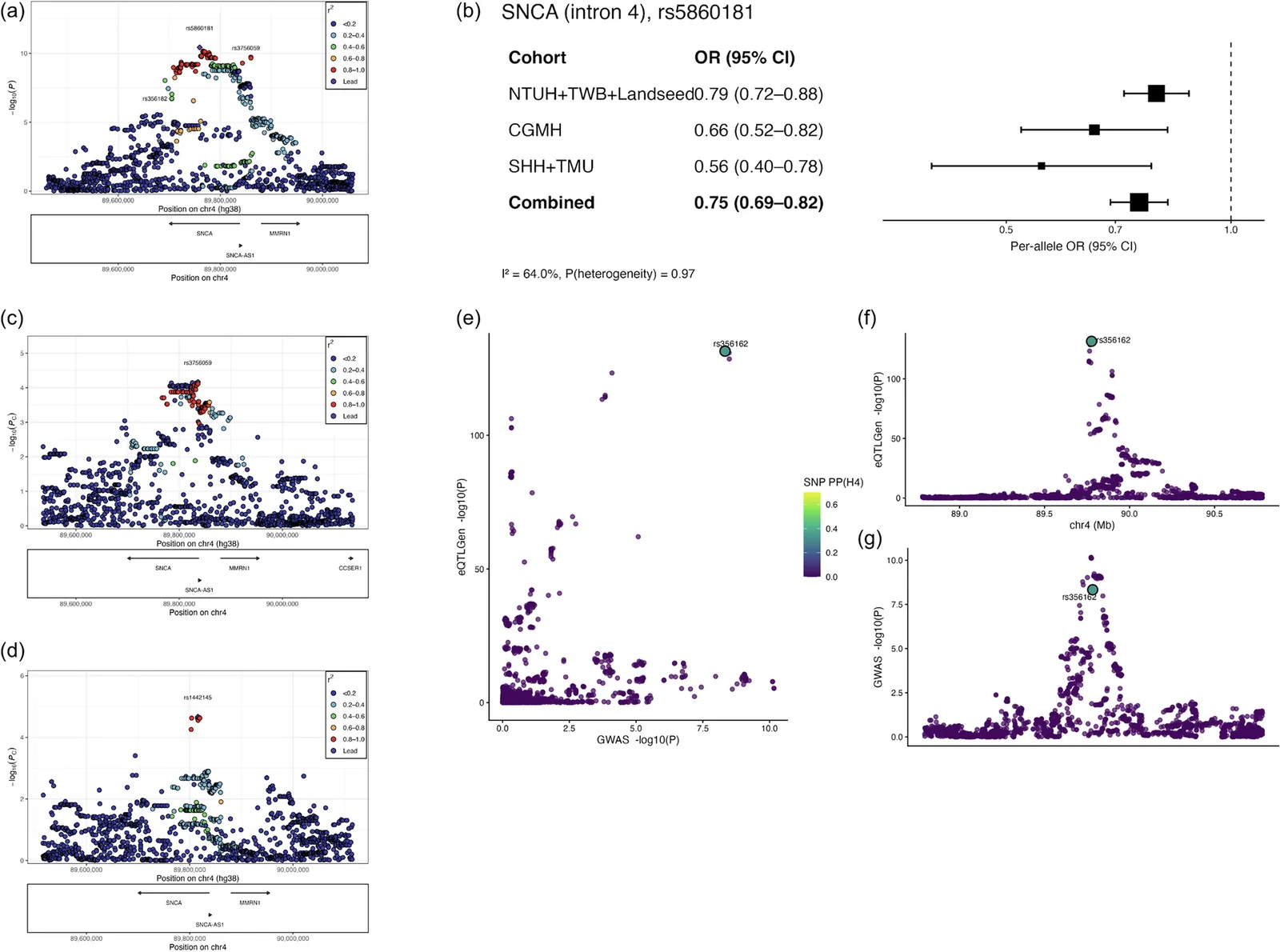
SNCA regional association, site-level meta-analysis, conditional analysis, and colocalization. **a–d** LocusZoom-style regional association plots show −log₁₀(P) versus genomic position across ±300 kb of the indicated index SNP. Linkage disequilibrium (LD; r²) is calculated in cohort controls; the index SNP is shown in purple and other variants are colored by r² with the index. **a** **SNCA** regional association plot indexed on the Taiwan lead variant **rs5860181** (chr4:89,759,478). The European index variant **rs356182** is also labeled. The 5′ UTR variant **rs3756059** additionally reaches genome-wide significance. **b** Site-level GWAS followed by meta-analysis at the *SNCA* locus (index rs5860181). **c** Conditional analysis conditioning on **rs356182**, identifying an additional 5′ signal at **rs3756059** (chr4:89,836,121; conditional β = −0.26, *P* = 6.85e − 5). **d** Conditional analysis conditioning on **rs5860181**, revealing a secondary 5′ signal at **rs1442145** (OR = 0.82, *P* = 1.88e − 5). **e–g** Colocalization of *SNCA* GWAS and whole-blood eQTL signals. **e** LocusCompare plot of *SNCA* GWAS versus **eQTLGen (whole blood)** association signals (−log₁₀ P). Points are colored by SNP-level posterior probability of colocalization (SNP PP.H4) from R “coloc”. The eQTL lead variant **rs356162** shows higher posterior support for a shared causal signal with the GWAS association (PP.H4 = 0.67). **f**, **g** Regional association plots across the same *SNCA* window for the GWAS and eQTLGen signals, illustrating concordant association peaks consistent with colocalization at this locus.

The lead association was **rs5860181** within intron 4 of *SNCA* (odds ratio [OR] = **0.75**, 95% CI 0.68–0.84, *P* = **3.7** **×** **10**^**–11**^) **(**Fig. 2**)**. Notably, the **5′ UTR variant rs3756059**, previously reported in **RBD**^13^ and **PD with RBD**^14^, also reached genome-wide significance (Table 2). A prominent signal was detected at **MCCC1** (rs34822404; OR = **1.31**, 95% CI 1.19–1.44, *P* = **7.2** **×** **10**^**–10**^), consistent with prior East Asian reports. At ***GCH1***, we replicated association at **rs3783640** (OR = **1.23**, 95% CI 1.09–1.39, *P* = **7.7** **×** **10**^**–7**^), which is in linkage disequilibrium with the previously reported rs11158026 signal. (Supplementary Fig. S4)

Among suggestive loci, ***PPARGC1A*** (rs609501; OR = 1.22, 95% CI 1.08–1.38, *P* = 3.5 × 10^–6^) and ***GALNT13*** (rs10186542; OR = 1.35, 95% CI 1.01–1.45, *P* = 5.3 × 10^–6^) have not, to our knowledge, been reported in prior PD GWAS and warrant follow-up. We also observed the expected positive effects for ***LRRK2*** p.G2385R and p.R1628P (Table 3). Finally, variants within ***VPS13C*** and ***SCARB2/FAM47E*** that were not in high LD with previously reported index variants from Nalls et al. or Foo et al. (Supplementary Fig. S3e-f) suggest potentially Taiwan-specific signals. Of note, the protective VPS13C signal at rs12900645 (chr15:61,994,009; hg38) lies within the putatively protective haplotype p.R153H–p.I398I–p.I1132V–p.Q2376Q (chr15:61,920,582–62,023,836) reported by Rudakou et al.^15^ In Taiwanese controls, linkage disequilibrium between **rs12900645** and **p.I1132V** was modest (**r²** ≈ **0.44**), indicating only partial tagging of the Rudakou haplotype. Across the four coding/synonymous variants reported by Rudakou et al., point estimates were protective but not statistically significant (e.g., **p.I1132V OR** = **0.91,** ***P*** = **0.08**). These findings are consistent with a protective background spanning **chr15:61,920,582–62,023,836**; however, in the Taiwanese cohort the **intronic rs12900645** is the **lead marker** of the protective effect in *VPS13C*.

**Site-level GWAS, meta-analysis, and sensitivity analysis** (Fig. 2b, Supplementary Fig. S5, and Supplementary Table S3)

Because an independent external Taiwanese PD cohort was unavailable, we performed site-stratified GWAS and meta-analysis across three strata: **NTUH** + **TWB + Landseed,** **CGMH**, and **SHH** + **TMU biobank**. (Recruitment site information is summarized in Supplementary Table S1). Site-level PCA showed no obvious clustering by recruitment site (Supplementary Fig. S2a–c). The major signals at ***SNCA*** and ***MCCC1*** remained significant across all sites and site-level meta-analysis (Fig. 2b and Supplementary Fig. S6). Compared with the overall Taiwan GWAS, ***GCH1*** and ***PPARGC1A*** were less significant after stratification, likely owing to reduced power. At ***VPS13C*** (**rs12900645**), the effect direction was consistent in **NTUH** + **TWB + Landseed** and **CGMH**, whereas **SHH** + **TMU** showed a non-significant opposite direction; however, the combined meta-analysis remained significant. In contrast, ***WWOX*** (**rs9922439**) showed stronger evidence in the site-level meta-analysis (**OR** = **1.25,** ***p*** = **1.18** **×** **10**^**–6**^) than in the overall Taiwan GWAS (**OR** = **1.21,** ***p*** = **1.21** **×** **10**^**–5**^). Leave-one-site(-stratum)-out and NTUH-only analyses further supported the robustness of most lead loci (Supplementary Table S3).

***SNCA*** **and** ***SCARB2/FAM47E*** **conditional analysis** (Fig. 2c, d and Supplementary Table S4). We further conducted conditional and joint analyses implemented in GCTA-COJO^16^. At the *SNCA* locus, the Taiwan intron 4 lead variant rs5860181 remained associated after conditioning on the European 3′ region variant rs356182 (conditional β = −0.13, *P* = 0.002). Under the same conditional model, we detected an additional 5′ *SNCA* signal at rs3756059 (chr4:89,836,121; conditional β = −0.26, *P* = 6.85e − 5), a variant previously reported to be associated with REM sleep behavior disorder (RBD)^13^. Conversely, conditioning on rs5860181 attenuated the rs356182 signal (conditional β = −0.03, *P* = 0.45), while another 5′ signal rs1442145 (chr4:89,815,772) showed residual evidence of association (conditional β = −0.20, *P* = 2.1e − 5). In joint modeling, rs5860181 remained significant when modeled together with rs356182 (rs5860181 *P* = 3.1e − 5; rs356182 *P* = 0.31). When jointly modeling four variants (rs5860181, rs356182, rs1442145, rs3756059), rs5860181 (*P* = 1.0e − 4) and rs1442145 (*P* = 0.007) remained significant.

At the *SCARB2/FAM47E* locus, the European lead variant rs11547135 (chr4:76,213,633) recently reported by Sun et al.^17^ lies ~3.5 kb from our Taiwan lead variant rs76591264. Using GCTA-COJO two-variant joint modeling, rs76591264 remained more significant (joint β = 0.138, P_joint = 0.00759) than rs11547135 (joint β = −0.105, P_joint = 0.0436), supporting rs76591264 as the stronger independent association in our cohort. The two variants showed a moderate inverse correlation in the LD reference (r = −0.44), consistent with partial LD rather than complete collinearity.

**Table 4:** Distribution of East Asian *LRRK2* risk genotypes and associated PD odds

A				
LRRK2 risk variant number	0	1*	2**	*LRRK2* carrier rate
Control (n = 2268)	2047	216	5	9.7%
PD (n = 2251)	1935	297	19	14.0%
Onset age	60.75 ± 11.19	59.17 ± 11.25	62.00 ± 9.38	One-way ANOVA: *p* value 0.07
B				
	Odds ratio	95% CI		*p* value
single variant vs. non-carrier	1.49	1.23–1.79		4.1E–05
double variants vs. non-carrier	4.29	1.59–11.6		0.004
double variants vs. single variant	2.89	1.06–7.9		0.039

Odds ratios from logistic regression adjusted for age, sex, and top 15 PCs; reference = noncarriers. “Double-variant” includes G2385R/G2385R, R1628P/R1628P, and G2385R/R1628P compound heterozygotes.

* either *LRRK2* G2385R or R1628P one variant carrier.

** double variants : G2385R homozygous, R1628P homozygous or G2385R + R1628P compound heterozygous.

### Genotype analyses of *LRRK2* risk alleles (Table 4)

The combined carrier rate of East Asian *LRRK2* risk variants was **9.7%** in controls and **14.0%** in PD. Relative to noncarriers, individuals carrying **one** variant (either p.G2385R or p.R1628P) had **OR** = **1.49** (95% CI 1.23–1.79; ***P*** = **4.1** **×** **10**^**–5**^). Carriers of **two** risk variants (p.G2385R homozygotes, p.R1628P homozygotes, or p.G2385R/p.R1628P compound heterozygotes) had **OR** = **4.30** (95% CI 1.59–11.6; ***P*** = **0.004**) versus noncarriers and **OR** = **2.89** (95% CI 1.06–7.91; ***P*** = **0.039**) versus single-variant carriers, consistent with a gene-dosage effect.

Age at onset (mean ± SD) did not differ significantly across groups: noncarriers **60.75** ± **11.19** years, one-variant carriers **59.17** ± **11.25** years, and two-variant carriers **62.00** ± **9.38** years. In an exploratory subgroup comparison, **compound heterozygotes (p.G2385R/p.R1628P)** showed a numerically earlier onset (**55.6** ± **5.8** years) versus noncarriers (**60.75** ± **11.19** years), a trend that did not reach significance (***t*** **test** ***P*** = **0.06**); this analysis is limited by small sample size.

**Fig. 3:**
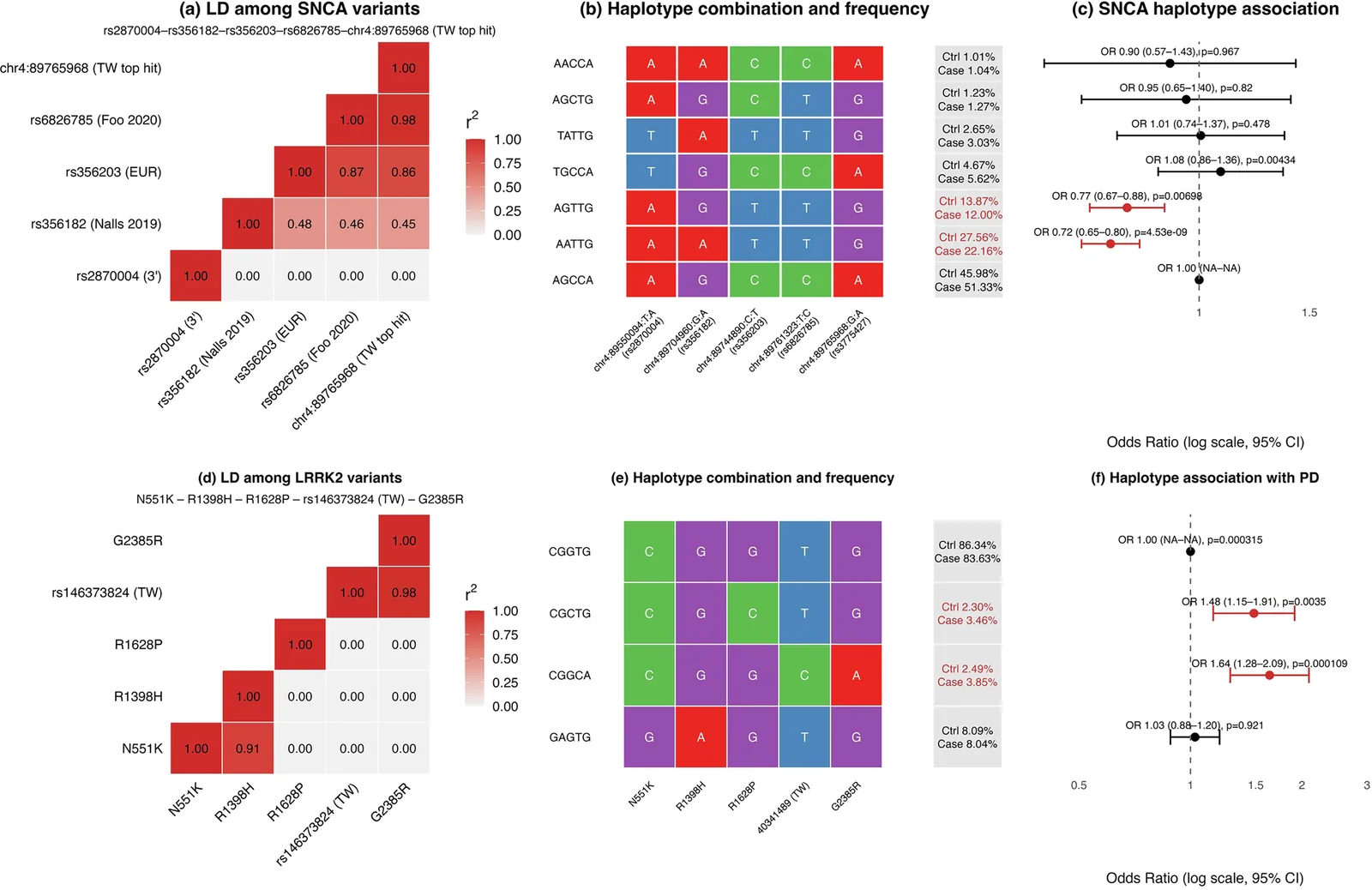
Haplotype structure and association of *SNCA* and *LRRK2* in the Taiwanese Parkinson’s disease cohort. **a** Linkage disequilibrium (LD) heatmap (r²) among five representative *SNCA* variants (rs2870004, rs356182, rs356203, rs6826785, rs3775427) computed in controls. **b** Case–control frequencies of common *SNCA* haplotypes (allele order as in panel a); bases are color-coded by nucleotide. Protective haplotypes **AGTTG** and **AATTG** are highlighted. **c** Forest plot of *SNCA* haplotype associations (odds ratio [OR] with 95% confidence interval [CI]; log scale) with corresponding *P* values; **AGTTG** and **AATTG** show reduced PD risk. **d** LD heatmap (r²) for five *LRRK2* missense/region-specific variants—N551K (rs7308720), R1398H (rs7133914), R1628P (rs33949390), chr12:40341489, and G2385R (rs34778348)—illustrating an East Asian–patterned LD structure. **e** *LRRK2* haplotype composition and case–control frequencies (allele order as in panel **d**). **f** Forest plot of adjusted *LRRK2* haplotype associations (OR, 95% CI, P). Haplotypes **CGGCA** and **CGCTG** exhibit protective effects relative to the reference **CGGTG**. LD linkage disequilibrium, OR odds ratio, CI confidence interval. **Analysis details:** LD was estimated with PLINK v1.9 (–r²) in controls. Haplotypes were inferred by EM (PLINK) and tested for association using the R package **haplo.stats**, with models adjusted for age, sex, and the first five principal components.

### Haplotype structure at *SNCA*, *LRRK2*, and *GCH1* (Fig. 3)

To probe ancestry-specific architecture, we performed regional haplotype analyses for *SNCA*, *LRRK2*, and *GCH1*.

### SNCA

The lead variant **rs3775427** (intron 4) resides on an East Asian–enriched haplotype distinct from the European **rs356182**–centered configuration. Using 1000 Genomes East Asian reference data via LDlink, **rs3775427**—the top *SNCA* signal in Taiwan—was tightly correlated with **rs6826785** (D′ = 0.996, r² = 0.988) and only partially correlated with **rs356182** (D′ = 0.83, r² = 0.469). Haplotype analysis of five representative variants (rs2870004, rs356182, rs356203, rs6826785, rs3775427) identified two common configurations, **AGTTG** and **AATTG**, that were associated with reduced PD risk (Fig. 3a–c). Notably, both protective haplotypes carry the **rs3775427-G** allele, whereas the **rs356182** allele differs between them (A vs. G), consistent with **rs3775427-G** (or variants on its tight LD background) tagging the protective effect. These findings support population-specific LD blocks at *SNCA*, with the Taiwanese pattern aligning more closely with other East Asian cohorts than with Europeans.

### LRRK2

**Fig. 4:**
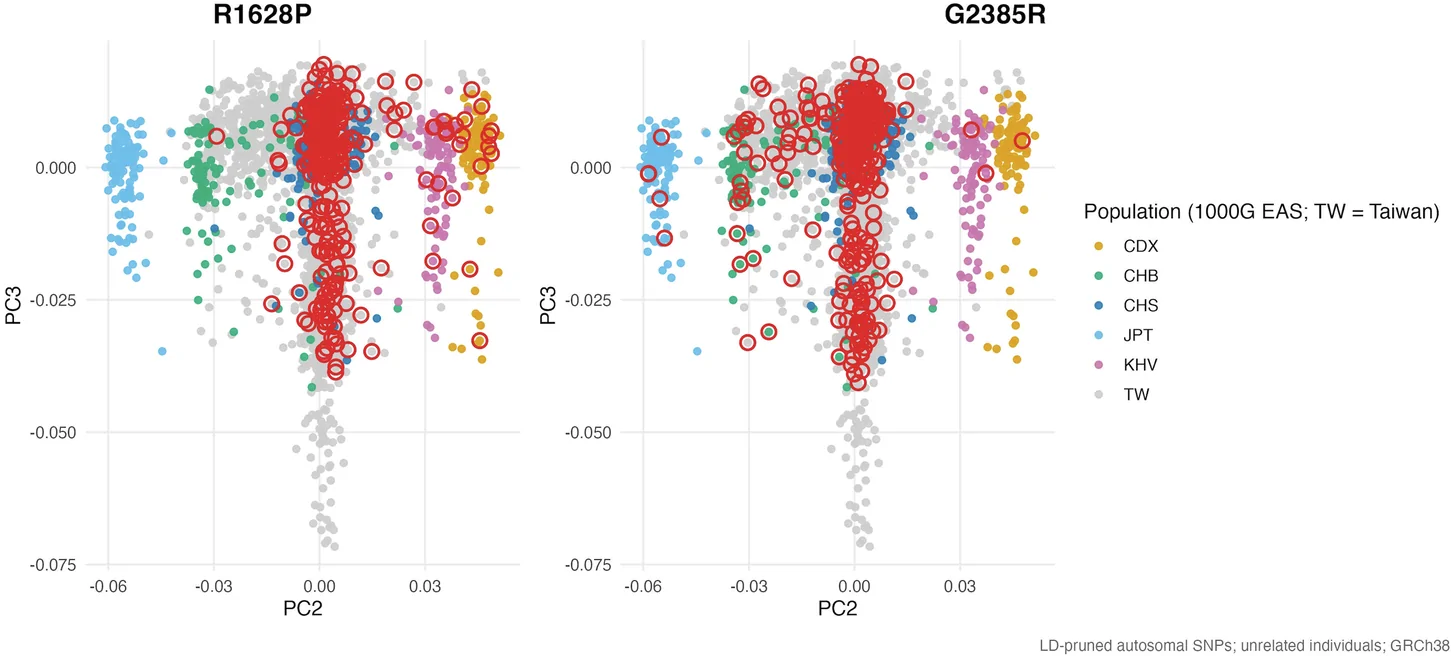
LRRK2 p.R1628P and p.G2385R on East Asian population PCA (PC2–PC3). Principal component analysis using LD-pruned autosomal SNPs (GRCh38) from the 1000 Genomes East Asian populations (CDX, CHB, CHS, JPT, KHV) and a Taiwanese cohort (TW). Points are colored by population; carriers of *LRRK2* p.R1628P (left) and p.G2385R (right) are indicated by red hollow circles. Related individuals were removed prior to PCA.

We evaluated five missense/region-specific variants—p.**N551K** (rs7308720), p.**R1398H** (rs7133914),p. **R1628P** (rs33949390), an intronic variant at chr12:40341489, and East Asian–specific p.**G2385R** (rs34778348). LD patterns indicated three largely independent variant groups (N551K–R1398H; R1628P; G2385R). Haplotype reconstruction followed by adjusted logistic regression identified **CGGCA** and **CGCTG** as **risk** haplotypes relative to the reference **CGGTG**, whereas the N551K–R1398H combination did not show a significant protective effect (Fig. 3d–f). Further principal component analysis (Fig. 4) shows that carriers of both *LRRK2* Asian variants cluster mainly within CHS (Southern Han Chinese). p.R1628P carriers are additionally observed in CDX (Chinese Dai) and KHV (Kinh Vietnamese), suggesting a more southern extension, whereas p.G2385R carriers are also seen in CHB (Han Chinese in Beijing) and JPT (Japanese), indicating a more northeastern distribution.

### GCH1

In Taiwan, **rs3783640** is in LD with **rs11158026** reported previously; both are only in partial LD with **rs841** (r² ≈ 0.31; Supplementary Fig. 2). Haplotype analyses indicated that configurations carrying **rs3783640** and **rs11158026** are associated with PD risk, whereas **rs841** alone was not significantly associated, suggesting that prior protective signals attributed to rs841 may be mediated by rs11158026/rs3783640.

Together, these analyses illustrate that while *SNCA*, *LRRK2*, and *GCH1* are globally implicated in PD, internal LD and haplotypic configurations differ by ancestry, underscoring the value of population-tailored analyses.

### Colocalization analysis

At ***SNCA***, colocalization with whole-blood cis-eQTLs from **eQTLGen** supported a shared signal (**PP.H4** = **0.67**; 2627 SNPs) (Fig. 2e–g). The lead PD GWAS variant (rs5860181) and lead blood eQTL variant (rs356162) fell within the same LD block in the Taiwanese reference (D′ = 1; r² = 0.21), consistent with a shared regional association despite different lead variants. In **metaBrain EAS cortex** eQTLs, evidence was limited (**PP.H4** = **0.08**).

For *PPARGC1A* (rs609501 region), colocalization was not supported in eQTLGen (PP.H4 = 0.08) or metaBrain EAS cortex, and nearby expressed genes also showed uniformly low support (PP.H4 < 0.1). At *VPS13C*, colocalization was not supported in whole blood (PP.H4 = 0.05) or metaBrain EAS cortex (PP.H4 = 0.04). Although the lead PD and blood eQTL variants were in the same LD block (D′ = 0.97; r² = 0.56), the pattern was more consistent with coincident LD-driven signals than a shared causal variant. At *SCARB2/FAM47E*, colocalization was not supported for *SCARB2* in whole blood (PP.H4 = 0.014) or cortex (PP.H4 = 0.020). A nearby gene, *THAP6*, showed only weak blood evidence (PP.H4 = 0.121).

### Functional annotation of associated loci

**CADD-based annotation** showed that both ***LRRK2*** **p.G2385R** and **p.R1628P** had high CADD scores (**23.200** and **26.400**), supporting the functional impact. **rs11547135** at the **SCARB2/FAM47E** locus also showed a potentially deleterious CADD score (**10.780**).

To assess the potential regulatory relevance of the associated variants, we performed functional annotation using GTEx v10 and SCREEN (Search Candidate Regulatory Elements by ENCODE) (Supplementary Tables S7 and S7B). At the ***SNCA*** locus, multiple variants showed regulatory evidence. In GTEx, **rs3756059, rs6826785, and rs356182** were associated with ***SNCA*** expression across several brain regions, including basal ganglia and cerebellar tissues. Consistently, SCREEN annotation showed that the Taiwan ***SNCA*** lead variant **rs5860181** overlapped two **distal enhancer** elements, whereas the 5′ variant **rs3756059** mapped to a **promoter** element, supporting regulatory complexity across this locus. Additional brain eQTL support was observed for ***GCH1*** variants (**rs11158026** and **rs3783640**) and for **rs12900645**, which was associated with ***VPS13C*** expression; notably, **rs12900645** also overlapped a **distal enhancer** in SCREEN. **rs34822404** at ***MCCC1*** overlapped a **CA–H3K4me3** regulatory element, whereas variants at ***MCCC1***, ***GALNT13***, and ***PPARGC1A*** showed predominantly peripheral-tissue eQTL association in GTEx. At the ***SCARB2/FAM47E*** locus, the Taiwan-associated variants **rs76591264** mapped to **distal enhancers**, while nearby previously reported European-region variants **rs11547135** overlapped **promoter** or **proximal enhancer** elements. No nearby cCREs were identified for ***GCH1*** **(rs3783640),** ***PPARGC1A*** **(rs609501)**, or ***GALNT13*** **(rs10186542)**. Overall, these annotations support a regulatory basis for several of the observed associated loci.

**Fig. 5:**
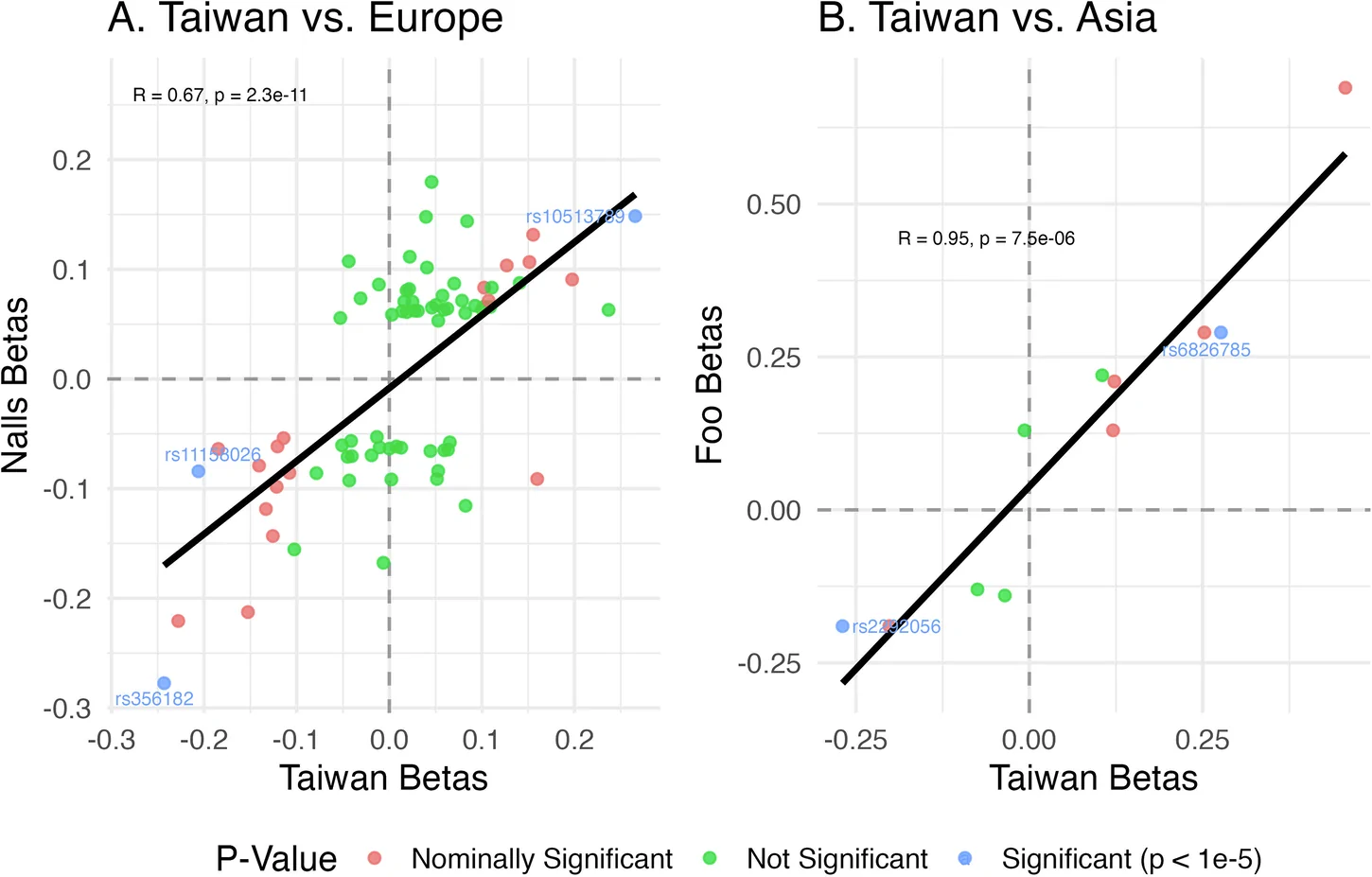
Replication of genome-wide significant variants from Nalls et al. (2019) and Foo et al. (2020). Scatter plots compare the effect sizes (log odds ratios) of PD-associated variants identified in previous GWAS with those observed in the Taiwanese cohort. For detailed statistics, please refer to Supplementary Table 4. Green points represent variants that were not statistically significant in the current dataset. A modest correlation was observed with the European meta-analysis by Nalls et al. (2019) (R = 0.67, *p* = 2.3 × 10⁻¹¹), reflecting limited cross-ancestry transferability of risk alleles. In contrast, effect sizes showed a strong positive correlation with the pan-Asian GWAS by Foo et al. (2020) (R = 0.96, *p* = 7.5 × 10⁻⁶), indicating a high degree of genetic concordance and shared PD risk architecture within East Asian populations.

### Cross-ancestry comparison (Fig. 5; Supplementary Table S3)

We compared effect sizes in this Taiwanese GWAS with those from a large European meta-analysis (Nalls 2019) and a pan-East Asian GWAS (Foo 2020). Scatter plots of log-odds ratios showed a **modest but significant** correlation with European estimates (Pearson’s r = **0.67**, *P* = **2.3** **×** **10**^**–11**^), with over half of variants not reaching significance in our cohort (green points in Fig. 3a). In contrast, effects were **strongly concordant** with East Asian estimates (r = **0.96**, *P* = **7.5** **×** **10**^**–6**^), reflecting shared risk architecture within East Asia. Notably, the ***MCCC1*** risk allele exhibited a larger effect in the Taiwanese cohort (OR = **1.32**) compared with European cohorts (OR ≈ **1.1**), suggesting population-specific modulation of genetic risk.

**Fig. 6:**
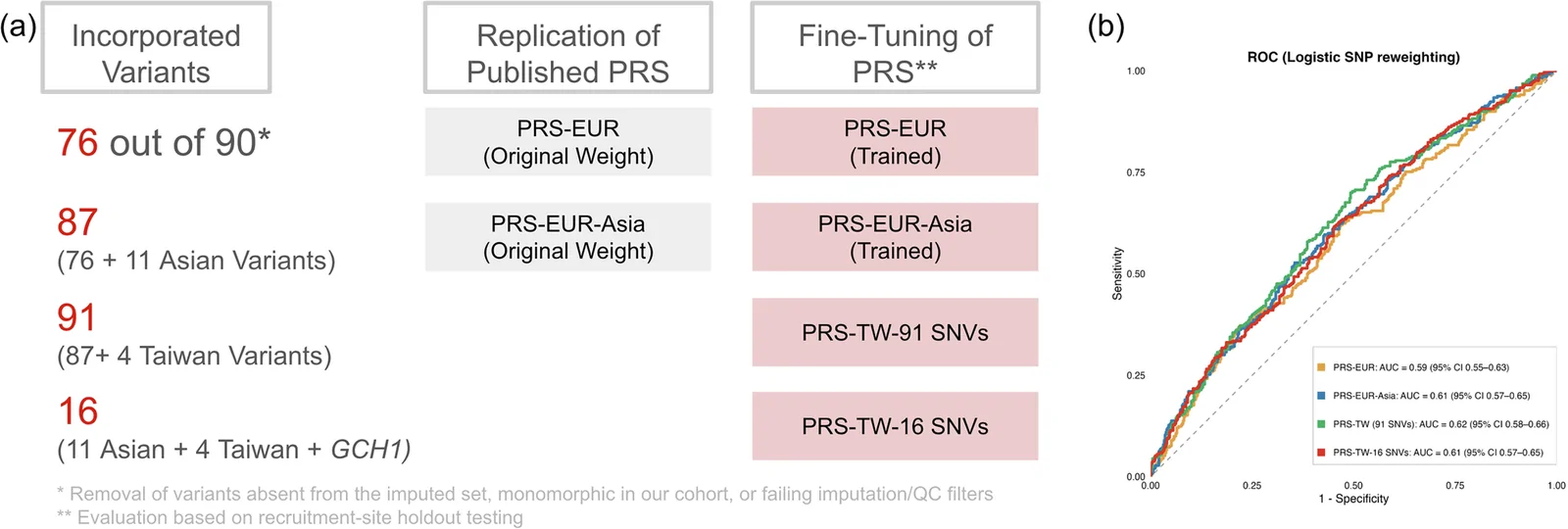
Polygenic risk score (PRS) performance for Parkinson’s disease in the Taiwanese cohort. **a** Overview of the PRS training and evaluation workflow using recruitment-site holdout testing. **b** Site-separated out-of-sample PRS evaluation with **CGMH** held out as an independent test set (290 PD cases / 451 controls; ~16% of the cohort). PRS models were trained on the remaining sites and evaluated in CGMH: (i) **Nalls (EUR-only)**, (ii) **Nalls + Asia**, (iii) **Nalls + Asia + Taiwan (4 variants)**, and (iv) **TW-16SNV (Asia + Taiwan** + ***GCH1*)**. In the held-out CGMH test set, AUCs (95% CI) were **0.59 (0.55–0.63)** for PRS-EUR (76 SNVs), **0.61 (0.568–0.651)** for PRS-EUR+Asia (87 SNVs), **0.62 (0.579–0.662)** for PRS-EUR+Asia+Taiwan (91 SNVs), and **0.61 (0.567–0.650)** for TW-16SNV (16 SNVs).

### Polygenic risk scores and population-specific improvement (Fig. 6)

We evaluated PRS models to test whether adding East Asian and Taiwan-relevant variants improves discrimination. Using published EUR weights (Nalls et al.), **76 of 90** variants were retained after harmonization and QC, and **PRS-EUR** achieved an AUC of **0.590** (95% CI, **0.574–0.607**). Adding **11** East Asian lead variants (Foo et al., 2020) yielded **PRS-EUR-Asia (87 variants)** and improved AUC to **0.606** (95% CI, **0.590–0.623**) (Supplementary Fig. S7).

To avoid overfitting when incorporating Taiwan-specific variants, we used a site-separated out-of-sample design, holding out **CGMH** as an independent test set (290 cases/451 controls) and training on the remaining sites. We evaluated **PRS-EUR,** **PRS-EUR-Asia,** **PRS-TW (91 variants)** by adding four Taiwan-relevant variants (*LRRK2* p.G2385R, *LRRK2* p.R1628P, *VPS13C* rs12900645, *SCARB2/FAM47E* rs76591264), and **PRS-TW-16 SNVs** (11 East Asian + 4 Taiwan + GCH1). In the held-out CGMH set, AUCs (95% CI) were **0.59 (0.55–0.63),** **0.61 (0.568–0.651),** **0.62 (0.579–0.662)**, and **0.61 (0.567–0.650)**, respectively (Fig. 6b), indicating that a compact Taiwan-focused model can achieve discrimination comparable to larger EUR-derived PRS models.

## Discussion

This is the first large-scale genome-wide association study (GWAS) of Parkinson’s disease (PD) in a Taiwanese cohort. It refines PD genetics in East Asia and underscores the value of ancestry-specific analyses. We replicated core PD loci (*SNCA, MCCC1, GCH1*) and East Asian *LRRK2* risk alleles (p.G2385R and p.R1628P), supporting conserved biological pathways across ancestries.^2,3,18^ At the same time, we observed clear ancestry-patterned features at *SNCA*. The Taiwanese lead variants **rs3775427** and **rs5860181** (both intron 4) reside on an East Asian–enriched haplotype. LDlink analyses using 1000 Genomes East Asian data showed tight correlation with **rs6826785** (D′ ≈ 0.996, r²≈0.988) but only partial correlation with the European lead **rs356182** (D′ ≈ 0.83, r²≈0.47), consistent with pan-Asian GWAS architecture.^2,3,9^ Conditional analyses reinforced this pattern: the intron-4 variants **rs5860181** remained associated when conditioning on **rs356182**, whereas the effect at **rs356182** was attenuated when conditioning on **rs5860181**. We also identified evidence for a possible secondary **5′** ***SNCA*** signal, including **rs3756059**, a variant previously implicated in **RBD** and **PD with RBD**^13,14^. This observation raises the possibility that **5′ regulatory variation at** ***SNCA*** may contribute not only to PD susceptibility but also to phenotypic heterogeneity. Notably, the LARGE-PD consortium, which performed a similar conditional analysis (conditioning on rs356182), did not observe any residual intron-4 or 5’ UTR signal^19^. This contrast implies that the intron-4 association may be more prominent in the Taiwanese and broader East Asian populations. Haplotype-based association identified two protective configurations (**AGTTG,** **AATTG**) that both carry rs3775427-G; notably, **AGTTG** retains rs356182-G on the canonical East Asian background.

Together, these findings indicate that variation within the intron 4 region contributes meaningfully to disease risk, and that *SNCA* risk and protection in Taiwan are better understood in the context of regional haplotypes rather than single variants. The putative **5′ UTR** ***SNCA*** signal will require validation in independent East Asian cohorts, ideally with phenotype-informed analyses including **RBD**. This has translational relevance as α-synuclein–targeted assays and therapies evolve, since underlying genetic polymorphisms may differentially influence α-synuclein expression, conformation, and treatment response.^20^

***LRRK2*** provides another example of ancestry-enriched PD risk architecture in East Asians. In our cohort, the two major Asian risk variants, **p.R1628P** and **p.G2385R**, were observed on distinct haplotypic backgrounds, consistent with previous reports that these alleles have different geographic distributions within East Asia^5,21,22^. By contrast, the commonly described East Asian protective haplotype **p.N551K–p.R1398H** was not significantly protective in our dataset^23^, underscoring possible heterogeneity even within Asian populations. Functionally, **p.R1628P** is located in the **COR domain** and has been reported to enhance *LRRK2* interaction with **Cdk5**^7,24^, leading to increased kinase activity, whereas **p.G2385R**, located in the **WD40 domain**^25,26^, appears to have more complex consequences, including altered domain structure, impaired vesicle-related interactions, and context-dependent effects on kinase activity.

The observed gene-dosage effect may reflect the structural biology of ***LRRK2*** rather than simply a cumulative statistical burden. Because *LRRK2* forms dimers and the **WD40 domain** contributes to dimerization^25^, different combinations of wild-type and mutant subunits may generate distinct functional outputs. In this context, the higher PD risk in double-variant carriers^27^ may reflect enhanced kinase dysregulation in mutant-containing complexes. This interpretation is supported by recent functional data showing that **double-variant carriers (p.G2385R** + **p.R1628P)** have markedly increased **pRab10Thr73 phosphorylation** compared with controls, consistent with heightened *LRRK2* pathway activation^28^. Taken together, these findings suggest that combined Asian ***LRRK2*** risk variants may exert additive or synergistic biological effects and support the relevance of genotype-informed stratification in future mechanistic and therapeutic studies.

Given the relative enrichment of double-variant carriers (**0.8%** of PD cases vs **0.2%** of controls) in Taiwan, these individuals represent a priority group for prodromal PD research and preventive intervention. Notably, caffeine intake may modify PD risk among *LRRK2* variant carriers in Asian populations^29^, and first-in-class **type II kinase inhibitors** that stabilize the inactive *LRRK2* conformation have recently been reported^30^, highlighting opportunities for pharmacogenomic stratification and targeted prevention.

At *GCH1*, we replicated association at **rs3783640**, which is in LD with the previously reported **rs11158026** signal^31^; in contrast, **rs841-A** alone was not protective in our data, and haplotype analysis suggested that earlier protective observations attributed to rs841 are likely mediated by the correlation with rs11158026-T^32^. For *MCCC1*, the Taiwanese effect size (OR ≈ 1.32) exceeded European reports (≈1.1) and aligns with observations in southern Chinese cohorts.^33^ We also observed a suggestive association at ***PPARGC1A***, a master regulator of mitochondrial biogenesis/oxidative stress previously linked to PD risk^34^ or age at onset^35^ and to essential tremor (ET)^36^; rare-variant data in Han Chinese further support involvement of ***PPARGC1A*** in PD^34^, motivating cross-disorder analyses in Taiwan. ***PPARGC1A*** is a biologically plausible therapeutic node (PGC-1α). In Taiwan’s National Health Insurance Database, exposure to the PPARγ agonist **pioglitazone** was associated with a lower incidence of PD (adjusted HR **0.66**, 95% CI **0.57–0.78**; dose-dependent)^37^, whereas a phase 2 trial in early PD did not demonstrate disease-modifying effects.^38^ These apparently discordant findings suggest context-dependent efficacy. We hypothesize that the pioglitazone–PD association is modified by *PPARGC1A* genotype, warranting genotype-stratified pharmacoepidemiology and prospective validation studies.

Finally, signals within ***VPS13C*** and ***SCARB2*** not in high LD with previously reported leads suggest potential Taiwan-specific haplotypes that warrant replication and conditional fine-mapping^31^. In ***VPS13C***, we detected an intronic protective association at rs12900645 that lies within a previously described protective region (chr15:61,920,582–62,023,836) encompassing the p.R153H–p.I398I–p.I1132V–p.Q2376Q haplotype reported by Rudakou et al.^15^. Linkage disequilibrium between rs12900645 and p.I1132V was modest (r²≈0.44), and the four coding/synonymous variants showed protective but non-significant effects in our data, together suggesting a shared protective background with rs12900645 as the lead tag in Taiwanese individuals. Given that VPS13C encodes a lipid-transfer protein implicated in lysosomal–mitochondrial homeostasis, this signal is biologically plausible but requires confirmation.

***GALNT13***, a glycosyltransferase, emerged as a putative novel locus, echoing prior Asian evidence at ***WBSCR17*/*GALNT17***^3^ and raising the possibility that O-glycosylation pathways contribute to PD susceptibility in Asian populations^39^. Recent studies have also shown that glycation of α-synuclein promotes its aggregation and exacerbates neuroinflammatory responses.^40^

Cross-ancestry comparisons showed effect sizes that correlated modestly with European meta-analysis and strongly with pan-East Asian estimates, reflecting both shared biology and ancestry-patterned architecture.^2,3^ This also helps explain limitations in PRS transferability. For ***LRRK2***, the Nalls 90-variant panel includes European-informative variants such as **p.G2019S (rs34637584)**^41^, but this variant is extremely rare in East Asians and was absent or near-monomorphic in our dataset; in contrast, **rs76904798** was retained because it is available in our data. At ***GBA1***, several variants in the Nalls panel are rare or absent in East Asians and were not reliably represented in our imputed data, while the locus itself is difficult to evaluate by SNP-array imputation because of ***GBAP1*** and complex local structure. Conversely, Taiwanese-relevant variants such as **p.L444P/L483P**^42^ are not well captured by standard imputation^43^. Therefore, *GBA1* is not included in this study.

Consistent with limited cross-ancestry PRS portability, the **European-derived PRS** achieved modest discrimination in our cohort (**AUC** = **0.590**). Adding **East Asian-associated variants** improved the AUC to **0.606**, and further inclusion of **Taiwan-specific variants** increased it to **0.620**. These results show that incorporating ancestry-relevant variants improves risk prediction in Taiwanese PD.

Importantly, a **compact 16-SNV model** based on **Asia + Taiwan** + **GCH1** variants performed similarly to models that also included a larger set of European-derived variants, suggesting that a smaller, population-tailored panel may be sufficient for practical PRS application in Taiwanese and possibly other East Asian populations.

Functional follow-up provided supportive evidence at the *SNCA* locus, where colocalization with eQTLGen^44^ whole-blood cis-eQTLs suggested a shared regulatory signal. However, interpretation of these analyses is limited by the lack of adequately powered, ancestry-matched brain molecular QTL resources. Although we incorporated the metaBrain East Asian cortex eQTL^45^ dataset to improve ancestry matching, its modest sample size and incomplete locus-level variant coverage reduced power for colocalization and likely limited detection of shared causal signals. In addition, bulk cortex data may not capture cell type–specific or region-specific regulatory effects relevant to PD. Accordingly, our functional analyses should be regarded as supportive and hypothesis-generating rather than definitive, and future studies using larger East Asian brain eQTL datasets or cohort-matched molecular QTL resources will be important to refine the regulatory interpretation of population-specific signals.

Several limitations merit mention. First, we used SNP arrays with imputation rather than comprehensive sequencing, which reduces sensitivity to rare coding variants and structural variation and performs suboptimally at complex loci (e.g., *GBA1* with its pseudogene)^46^. Deep whole-genome sequencing and/or long-read approaches with locus-specific validation would address this. Second, this is a Taiwan-focused cohort designed to fill a clear gap; findings may not capture the full heterogeneity across East and Southeast Asia. Replication in independent EAPDGC sites^12^ and additional regional cohorts will be important. Third, Taiwan-specific variants have not yet been externally replicated because a truly independent Taiwanese dataset for evaluating Taiwan-specific components is currently unavailable. Likewise, although we held out certain recruiting sites as internal validation sets, the PRS-TW model was developed and evaluated within the same overarching cohort, introducing the potential for overfitting and optimistic bias in estimated discrimination. Accordingly, the observed AUC gains—particularly those attributable to Taiwan-specific variants—should be interpreted as preliminary until validated in an independent Taiwanese cohort. Moreover, the PRS performance was assessed using logistic regression without clinical or lifestyle covariates (e.g., caffeine^29^, smoking), prodromal markers, or biomarkers. Future work should evaluate integrative models that combine genetics with environmental and clinical features, consider machine-learning approaches^47^ with rigorous internal/external validation, and report calibration and clinical utility (e.g., decision-curve analysis).^48^

In conclusion, this Taiwan-wide GWAS refines PD genetics in East Asians and underscores the value of ancestry-specific analysis. We confirmed established loci (*SNCA, MCCC1, GCH1*), defined an East Asian intron 4 *SNCA* haplotype underlying risk and protection, and showed that the common Asian *LRRK2* variants p.G2385R and p.R1628P have a gene-dosage effect. We also identified a *VPS13C* protective region and suggestive associations at *PPARGC1A* and *GALNT13*, although these population-specific loci require external replication. A Taiwan-optimized PRS improved discrimination over a European-derived model, but because it was developed and tested within the same cohort, potential overfitting should be acknowledged and external validation remains necessary. These findings provide a basis for future replication in East and Southeast Asia, alongside fine-mapping, functional follow-up, and integration with clinical and environmental data.

## Methods

### Study population and cohort design

Participants were recruited through the East Asian Parkinson Disease Genomics Consortium (EAPDGC) via a multi-center collaboration. Taiwanese recruitment sites included National Taiwan University Hospital (NTUH), Chang Gung Memorial Hospital (CGMH), Shuang Ho Hospital/Taipei Medical University (SHH/TMU), Landseed Hospital and Taiwan Biobank.^49,50^ Parkinson’s disease (PD) diagnosis followed the Movement Disorder Society (MDS) Clinical Diagnostic Criteria.^51^ We excluded atypical parkinsonism and secondary parkinsonism, including drug-induced parkinsonism, vascular parkinsonism, and normal-pressure hydrocephalus. Participants with major comorbid neurological or psychiatric conditions that could confound PD diagnosis or clinical assessment (e.g., prior stroke with significant sequelae, epilepsy, schizophrenia) were also excluded. All participants provided written informed consent. The study protocol was approved by the NTUH Institutional Review Board (NTUH-REC No. 201905035RINC).

### Genotyping and quality control

Genotyping used the Illumina NeuroBooster Array, which combines the Global Diversity Array backbone with custom neuro-focused content.^43^ Quality control (QC) was performed in PLINK v1.9 and v2.0.^52,53^ Samples were excluded for low call rate (<95%), sex discordance, or excess relatedness (one of each pair with PI_HAT > 0.20 removed). Variants were removed for Hardy–Weinberg disequilibrium in controls (p < 1×10⁻⁴), missingness >5%, or minor allele frequency (MAF) < 0.005. Principal component analysis (PCA) was performed to control for population structure. Association models adjusted for age, sex, and ancestry PCs derived from study genotypes.

### Imputation

Post-QC genotypes were imputed using the Taiwan Biobank (TWB) imputation service, which employs Minimac4 and a population-specific reference panel of ~1500 TWB whole-genome sequences.^49,54^ Imputation was performed on the TWB server pipeline; we applied post-imputation filters of MAF > 0.01 and Rsq>0.30, yielding ~7.6 million high-quality variants for analysis.

### Principal component analysis (PCA)

Autosomal, biallelic SNPs were LD-pruned in PLINK v1.9 (parameters: --maf 0.10; --indep 50 5 1.5), and principal components were then computed on the pruned set using --pca. Study genotypes were merged with the 1000 Genomes Project Phase 3 (GRCh38) reference to anchor ancestry inference. Scatter plots of top PC were generated in R (v4.4.2) using ggplot2 to visualize clustering; participants aligned with the East Asian superpopulation (Fig. 3 and Supplementary Fig. S1).

### Statistical analysis

Genome-wide association testing used logistic regression in PLINK 2.0 with PD status as the outcome, adjusting for age, sex, and the top 15 principal components. Genome-wide significance was defined as *p* < 5 × 10^–8^ and suggestive significance as *p* < 1 × 10^–5^. Analyses were later conducted in R (v4.4.2).^55^ Specifically, we ran logistic regression in PLINK 2.0 using the --condition flag to include the index variant as a covariate, adjusting for age, sex, and the first 15 principal components.

### Site-level GWAS, meta-analysis and sensitivity analysis

We performed site-stratified GWAS across three recruitment strata (NTUH + TWB+Landseed, CGMH, and SHH + TMU) using logistic regression with covariate adjustment for age, sex, and ancestry principal components. Stratum-specific additive-model summary statistics were combined using PLINK 1.9 fixed-effects inverse-variance meta-analysis (plink19 --meta-analysis <study1.meta.in > <study2.meta.in > <study3.meta.in > + study --out <prefix > ), with between-stratum heterogeneity assessed by Cochran’s Q and I². Key loci were summarized using forest plots displaying per-allele odds ratios with 95% confidence intervals for each stratum and the combined estimate. (Supplementary Fig. 5 and Supplementary Table 4)

### Linkage disequilibrium and haplotype analysis

Population LD context was evaluated using LDlink to query East Asian reference panels.¹² Within the Taiwanese cohort, pairwise LD (r²) was estimated with PLINK (–r2). Haplotype visualization for *SNCA*, *LRRK2*, and *GCH1* used the **geneHapR** R package^56^; haplotype association testing employed **haplo.stats**^57^ with EM-based haplotype frequency estimation, adjusting for age, sex, and the first five PCs.

### GCTA-COJO (conditional and joint) analyses at *SNCA*

We applied GCTA-COJO^16^ to perform approximate conditional (cojo-cond) and joint (cojo-joint) analyses at the *SNCA* locus using GWAS summary statistics and an ancestry-matched LD reference panel derived from our study genotypes after QC.

### Functional annotation and colocalization analysis

Preliminary functional annotation is performed using SNPnexus^58^ (https://snpnexus.org/) and GTEx portal^59^ (https://gtexportal.org/home/). To further explore potential regulatory mechanisms underlying associated loci, we performed colocalization analyses using publicly available eQTL summary statistics. We leveraged eQTLGen for whole-blood eQTLs^44^ and brain eQTL resources—metaBrain (East Asian subset)^45^—for the genes of interest. Colocalization was conducted in R using the *coloc* package^60^, applying an approximate Bayes factor framework to estimate the posterior probabilities for five competing hypotheses (H0–H4), with primary interest in evidence supporting a shared causal variant between the GWAS and eQTL signals (H4).

Functional annotation was performed using **SNPnexus**^58^ and **GTEx Analysis Release V10**^59^. Histone modification and broader regulatory annotation were examined using **SCREEN (**https://screen.wenglab.org/**)**, which provides access to the ENCODE registry of **candidate cis-regulatory elements (cCREs)**.^61^

### Cross-ancestry replication and effect-size correlation

To assess cross-population consistency, we extracted significant variants from the European meta-analysis (Nalls et al., 2019) and the pan-Asian GWAS (Foo et al., 2020), harmonized alleles to GRCh38, and computed Pearson correlations of log-odds ratios between this study and reference datasets.

### Polygenic risk score construction and site-separated evaluation

We evaluated PRS models that progressively incorporated **European-, East Asian-, and Taiwan-relevant variants** (Fig. 6a). **PRS-EUR** used the published variant list and effect sizes from **Nalls et al**.^2^, after standard harmonization to our imputed dataset. **PRS-EUR-Asia** further added **11 independent East Asian lead variants** from **Foo et al**.^3^ For these two models, published effect sizes were used directly without re-estimating SNP weights.

To limit overfitting when adding Taiwan-specific variants, PRS performance was evaluated using a **recruitment-site holdout design**, with **CGMH** as the held-out test set in the primary analysis. All model fitting and score scaling were performed in the training sites only, and performance was assessed exclusively in the held-out site. We evaluated four models: **PRS-EUR,** **PRS-EUR-Asia,** **PRS-TW (91 SNVs)**, which added ***LRRK2*** **p.G2385R,** ***LRRK2*** **p.R1628P,** ***VPS13C*** **rs12900645**, and ***SCARB2/FAM47E*** **rs76591264**, and **PRS-TW-16 SNVs**, a parsimonious model including the **11 East Asian variants, 4 Taiwan variants, and** ***GCH1*** **(rs11158026)**. For the Taiwan-focused models, SNP weights were re-estimated in the training set by logistic regression and applied to the held-out site for out-of-sample prediction.

## Supplementary information

Supplementary information

Supplementary table7

## Data Availability

Summary statistics generated in this study will be made available through the GP2 platform. Access to the individual-level data used in the preparation of this article is coordinated by the corresponding authors and governed by the GP2 Tier 2 data access policy, and may be granted upon approval of the request and execution of a Data Use Agreement by the applicant’s institution. GP2 data are available via the AMP PD platform (https://amp-pd.org). Summary statistics from Nalls et al. were retrieved from the GWAS Catalog (https://www.ebi.ac.uk/gwas/), and summary statistics from Foo et al. were obtained from the supplementary materials of the original publication in JAMA Neurology.^3^ Publicly available eQTL summary statistics were used for colocalization analyses. Brain eQTL data were obtained from the metaBrain resource described by de Klein et al^45^, and whole-blood cis-eQTL summary statistics were obtained from the eQTLGen consortium website^44^ (cis-eQTLs; full cis-eQTL summary statistics).

## Code availability

All code generated for this article, and the identifiers for all software programs and packages used, are available on GitHub https://github.com/GP2code/Taiwan-GWAS-and-PRS and were given a persistent identifier via Zenodo DOI 10.5281/zenodo.18232864.
