# Supplementary material for: Genome-wide association and population-tailored polygenic risk for Parkinson’s disease in Taiwan
Source: NPJ Parkinsons Dis. 2026 May 22;12:191. doi: 10.1038/s41531-026-01349-0 (PMC13470500; doi:10.1038/s41531-026-01349-0)

Supplementary Table S1. Recruitment centers and sample sizes
List of hospitals and institutions contributing PD cases and controls

	Case before QC	Case After QC	Control Before QC	Control After QC
National Taiwan Univesity Hospital (NTUH)	1916	1850	900	846
Chang-Gung Memorial Hospital (CGMH)	317	290	484	451
Taiwan Biobank (TWB)			530	476
Shuang-Ho Hospital (SHH) and Taipei Medical University Biobank (TMU)	166	111	400	396
Landseed Biobank			130	99
Total Number	2399	2251	2444	2268

Supplementary Table S2: Full Genetic Risk Loci Associated with PD in Taiwan with p value less than 1e-5

Chr	Position (hg38)	SNP (rsID)	Nearest Gene	Effect Allele	Other Allele	MAF	OR_CI	β	SE	P	Annotation
2	154088802	rs10186542	*GALNT13*	A	G	0.12	1.35 (1.18–1.55)	0.13	0.07	5.30e-06	New
2	179727418	rs4894119	*ZNF385B*	G	A	0.02	2.11 (1.54–2.89)	0.32	0.16	5.20e-06	New
3	183017423	rs2292056	*MCCC1*	T	G	0.43	1.31 (1.21–1.42)	0.12	0.04	8.50e-10	Foo
3	183028420	rs34822404	*MCCC1*	C	T	0.43	1.31 (1.21–1.42)	0.12	0.04	7.20e-10	Most significat in Taiwan
3	183042285	rs10513789	*MCCC1*	T	G	0.43	1.3 (1.20–1.41)	0.12	0.04	1.40e-09	Nalls
4	24009221	rs609501	*PPARGC1A*	A	G	0.48	1.22 (1.13–1.32)	0.09	0.04	3.50e-06	New
4	89704960	rs356182	*SNCA*	A	G	0.3	0.78 (0.71–0.86)	-0.11	0.05	1.90e-07	Nalls
4	89744890	rs356203	*SNCA*	T	C	0.44	0.77 (0.71–0.83)	-0.11	0.04	9.50e-10	Past European
4	89759478	rs5860181	*SNCA*	A	ATGC ATATT	0.43	0.75 (0.69–0.81)	-0.12	0.04	3.70e-11	Most significat in Taiwan
4	89761323	rs6826785	*SNCA*	T	C	0.43	0.76 (0.70–0.82)	-0.12	0.04	1.60e-10	Foo
4	89765968	rs3775427	*SNCA*	G	A	0.43	0.75 (0.69–0.81)	-0.12	0.04	6.90e-11	Most significat in Taiwan
5	119707094	rs2194056	*AC008550.1*	C	G	0.04	0.57 (0.45–0.72)	-0.24	0.12	1.60e-06	New
5	131320353	rs185601781	*CDC42SE2*	G	C	0.02	0.52 (0.39–0.70)	-0.29	0.15	5.40e-06	New

7	153256105	rs12671235	*LOC102723686*	A	T	0.32	0.8 (0.73–0.88)	-0.09	0.05	2.70e-06	New
9	102260653	rs76204651	*RNU6-329P*	A	G	0.06	0.65 (0.54–0.78)	-0.18	0.09	5.80e-06	New
14	54885803	rs3783640	*GCH1*	T	C	0.46	1.23 (1.14–1.33)	0.09	0.04	7.70e-07	Taiwan
14	54882151	rs11158026	*GCH1*	C	T	0.46	1.23 (1.14–1.33)	0.09	0.04	1.40e-06	Nalls
17	43331428	rs78543719	*CCDC200*	A	G	0.02	0.45 (0.32–0.63)	-0.35	0.17	1.70e-06	New
17	70673898	rs17792098	*AC005771.1*	G	A	0.19	1.31 (1.16–1.47)	0.12	0.06	1.30e-06	New
18	51753748	rs3844136	*AC008550.1*	G	A	0.06	0.65 (0.54–0.78)	-0.19	0.09	4.30e-06	New

Supplementary Table S3. Meta-analysis and sensitivity analyses for selected loci.

We performed site-stratified GWAS across three recruitment strata (NTUH+TWB+Landseed, CGMH, and SHH+TMU) and combined stratum-specific effects using fixed-effects inverse-variance meta-analysis. For each selected variant, the table reports the effect estimate as **β = log(OR)** with **95% CI** and **P value** for the overall meta-analysis, three leave-one-out meta-analyses (excluding each stratum in turn), and the individual stratum-specific results. An additional single-center sensitivity analysis is also provided. TWB and Landseed contributed community-recruited controls only (no PD cases).

(a)

CHR	BP	Variant_label	Effect_allele	Meta_beta(95%CI)	Meta_P	LOO_no_NTUH_TWB_Landseed_beta(95%CI)	LOO_no_NTUH_TWB_Landseed_P	LOO_no_CGMH_beta(95%CI)	LOO_no_CGMH_P	LOO_no_SHH_TMU_beta(95%CI)	LOO_no_SHH_TMU_P
12	39993947	*LRRK2 rs141336855*	T	0.449 (0.201, 0.698)	3.95E-04	0.561 (0.056, 1.066)	2.93E-02	0.417 (0.142, 0.692)	2.95E-03	0.449 (0.192, 0.705)	6.04E-04
12	40320043	*LRRK2 p.R1628P*	C	0.362 (0.100, 0.623)	6.68E-03	0.597 (0.047, 1.146)	3.33E-02	0.322 (0.040, 0.605)	2.54E-02	0.340 (0.068, 0.613)	1.44E-02
12	40363526	*LRRK2 p.G2385R*	A	0.444 (0.194, 0.693)	4.90E-04	0.393 (-0.132, 0.919)	1.42E-01	0.428 (0.154, 0.702)	2.18E-03	0.469 (0.213, 0.725)	3.38E-04
14	54885803	*GCH1, rs3783640*	T	0.181 (0.095, 0.268)	4.30E-05	-0.001 (-0.183, 0.181)	9.92E-01	0.215 (0.121, 0.309)	7.67E-06	0.196 (0.105, 0.287)	2.32E-05
15	61994009	*VPS13C, rs12900645*	G	-0.176 (-0.268, -0.084)	1.67E-04	-0.106 (-0.292, 0.080)	2.62E-01	-0.167 (-0.267, -0.067)	1.07E-03	-0.203 (-0.299, -0.107)	3.27E-05
16	78393869	*WWOX, rs9922439*	G	0.221 (0.132, 0.311)	1.18E-06	0.234 (0.047, 0.421)	1.42E-02	0.227 (0.130, 0.324)	4.31E-06	0.213 (0.120, 0.306)	7.16E-06
2	154088802	*GALNT13, rs10186542*	A	0.342 (0.207, 0.477)	6.64E-07	0.348 (0.080, 0.616)	1.10E-02	0.359 (0.212, 0.506)	1.81E-06	0.324 (0.183, 0.466)	6.64E-06
3	183028420	*MCCC1, rs10186542*	C	0.278 (0.189, 0.368)	1.22E-09	0.254 (0.067, 0.442)	7.89E-03	0.285 (0.187, 0.383)	1.11E-08	0.279 (0.185, 0.372)	4.81E-09
4	24009221	*PPARGC1A, rs609501*	A	0.178 (0.089, 0.267)	8.45E-05	0.213 (0.023, 0.403)	2.79E-02	0.181 (0.085, 0.277)	2.32E-04	0.168 (0.075, 0.260)	3.66E-04

4	76217199	*SCARB2/FAM47E, rs76591264*	G	0.168 (0.074, 0.262)	4.52E-04	0.298 (0.100, 0.496)	3.22E-03	0.159 (0.057, 0.262)	2.23E-03	0.145 (0.047, 0.242)	3.60E-03
4	89704960	*SNCA (3' UTR), rs356182*	A	-0.241 (-0.337, -0.146)	7.16E-07	-0.399 (-0.604, -0.194)	1.40E-04	-0.232 (-0.336, -0.128)	1.19E-05	-0.213 (-0.312, -0.115)	2.14E-05
4	89759478	*SNCA (intron 4), rs5860181*	ATGCATATT	-0.283 (-0.372, -0.195)	3.44E-10	-0.471 (-0.660, -0.283)	8.90E-07	-0.258 (-0.354, -0.162)	1.38E-07	-0.261 (-0.353, -0.170)	2.28E-08
4	89765968	*SNCA (intron 4), rs3775427*	G	-0.283 (-0.372, -0.195)	3.84E-10	-0.469 (-0.658, -0.280)	1.20E-06	-0.258 (-0.354, -0.162)	1.48E-07	-0.262 (-0.354, -0.171)	2.16E-08
4	89815772	*SNCA (5' UTR), rs1442145*	G	-0.181 (-0.275, -0.087)	1.64E-04	-0.183 (-0.375, 0.008)	6.09E-02	-0.176 (-0.279, -0.074)	7.72E-04	-0.185 (-0.283, -0.087)	2.25E-04
4	89836121	*SNCA (5' UTR), rs3756059*	G	-0.390 (-0.526, -0.253)	2.05E-08	-0.532 (-0.829, -0.236)	4.28E-04	-0.379 (-0.527, -0.232)	4.91E-07	-0.367 (-0.507, -0.226)	3.08E-07

(b)

CHR	BP	Variant_label	Effect_allele	NTUH_TWB_Landseed_beta_ci	NTUH_TWB_Landseed_p	NTUH_only_beta_ci	NTUH_only_p	CGMH_beta_ci	CGMH_p	SHH_TMU_beta_ci	SHH_TMU_p
12	39993947	*LRRK2 rs141336855*	T	0.414 (0.128, 0.699)	4.54E-03	0.557 (0.193, 0.921)	2.73E-03	0.596 (0.013, 1.179)	4.52E-02	0.458 (-0.553, 1.469)	3.74E-01
12	40320043	*LRRK2 p.R1628P*	C	0.293 (-0.004, 0.590)	5.33E-02	0.176 (-0.174, 0.525)	3.25E-01	0.592 (-0.093, 1.277)	9.04E-02	0.605 (-0.314, 1.524)	1.97E-01
12	40363526	*LRRK2 p.G2385R*	A	0.458 (0.175, 0.742)	1.53E-03	0.504 (0.158, 0.851)	4.33E-03	0.517 (-0.085, 1.120)	9.25E-02	0.001 (-1.071, 1.073)	9.99E-01
14	54885803	*GCH1, rs3783640*	T	0.236 (0.137, 0.335)	3.10E-06	0.266 (0.146, 0.385)	1.32E-05	-0.012 (-0.238, 0.214)	9.19E-01	0.019 (-0.287, 0.324)	9.04E-01
15	61994009	*VPS13C, rs12900645*	G	-0.199 (-0.304, -0.093)	2.22E-04	-0.198 (-0.324, -0.073)	1.98E-03	-0.223 (-0.452, 0.006)	5.58E-02	0.122 (-0.198, 0.441)	4.55E-01
16	78393869	*WWOX, rs9922439*	G	0.218 (0.116, 0.319)	2.70E-05	0.220 (0.098, 0.342)	4.22E-04	0.188 (-0.041, 0.418)	1.08E-01	0.324 (0.002, 0.646)	4.89E-02

2	154088802	*GALNT13, rs10186542*	A	0.340 (0.184, 0.496)	1.93E-05	0.390 (0.198, 0.582)	6.81E-05	0.254 (-0.079, 0.587)	1.34E-01	0.520 (0.068, 0.973)	2.42E-02
3	183028420	*MCCC1, rs10186542*	C	0.286 (0.183, 0.388)	4.39E-08	0.278 (0.156, 0.400)	7.87E-06	0.244 (0.016, 0.471)	3.58E-02	0.277 (-0.055, 0.608)	1.02E-01
4	24009221	*PPARGC1A, rs609501*	A	0.169 (0.068, 0.269)	1.02E-03	0.251 (0.130, 0.373)	5.12E-05	0.163 (-0.068, 0.393)	1.66E-01	0.318 (-0.016, 0.652)	6.22E-02
4	76217199	*SCARB2/FAM47E, rs76591264*	G	0.131 (0.024, 0.237)	1.65E-02	0.148 (0.019, 0.276)	2.41E-02	0.216 (-0.023, 0.454)	7.67E-02	0.479 (0.124, 0.834)	8.13E-03
4	89704960	*SNCA (3' UTR), rs356182*	A	-0.198 (-0.306, -0.090)	3.19E-04	-0.231 (-0.359, -0.103)	4.05E-04	-0.291 (-0.532, -0.049)	1.82E-02	-0.685 (-1.076, -0.293)	6.09E-04
4	89759478	*SNCA (intron 4), rs5860181*	ATGCATATT	-0.230 (-0.330, -0.130)	6.99E-06	-0.269 (-0.390, -0.148)	1.28E-05	-0.421 (-0.647, -0.195)	2.59E-04	-0.584 (-0.923, -0.245)	7.26E-04
4	89765968	*SNCA (intron 4), rs3775427*	G	-0.231 (-0.331, -0.131)	6.46E-06	-0.271 (-0.392, -0.150)	1.15E-05	-0.424 (-0.652, -0.196)	2.64E-04	-0.569 (-0.909, -0.229)	1.04E-03
4	89815772	*SNCA (5' UTR), rs1442145*	G	-0.180 (-0.288, -0.072)	1.07E-03	-0.138 (-0.268, -0.008)	3.70E-02	-0.205 (-0.439, 0.029)	8.59E-02	-0.139 (-0.473, 0.195)	4.15E-01
4	89836121	*SNCA (5' UTR), rs3756059*	G	-0.351 (-0.505, -0.198)	7.07E-06	-0.320 (-0.505, -0.135)	6.83E-04	-0.447 (-0.797, -0.097)	1.23E-02	-0.750 (-1.308, -0.193)	8.36E-03

Supplementar Table S4. GCTA-COJO joint analysis of *SNCA* and *SCARB2/FAM47E* loci in the Taiwanese PD GWAS

Approximate conditional and joint association results for the *SNCA* and *SCARB2/FAM47E* loci were generated using GCTA-COJO. Each block corresponds to a specified joint model, with variants included in that model listed in the corresponding rows. For *SNCA*, variants include the Taiwan lead intron 4 variant rs5860181, the 3′ region lead variant rs356182, and two 5′ region variants rs1442145 and rs3756059 (previously reported for REM sleep behavior disorder and PD with RBD). For *SCARB2/FAM47E*, the joint model includes rs11547135 (EUR lead) and rs76591264 (Taiwan signal). **EAF** denotes effect-allele frequency. **Beta_marginal** and **SE_marginal** are the marginal (single-variant) log-odds effect estimate and standard error from GWAS summary statistics (**P_marginal**). **Beta_joint** and **SE_joint** are the joint effect estimate and standard error conditional on the other variant(s) included in the same joint model (**P_joint**).

Model	Chr	BP	rsID	Variant	Effect_ allele	Beta_ marginal	SE_ marginal	P_ marginal	Beta_ joint	SE_ joint	P_joint
SNCA joint_2vars (TW intron4 + Nalls 3′UTR)	4	89759478	rs5860181	*SNCA* intron 4 (rs5860181; Taiwan lead)	A	-0.286	0.043	3.729e-11	-0.245	0.059	3.054e-05
	4	89704960	rs356182	*SNCA* 3′ region (rs356182; EUR lead)	A	-0.243	0.047	1.889e-07	-0.065	0.063	3.088e-01
SNCA joint_3vars (+ RBD 5′ rs3756059)	4	89759478	rs5860181	*SNCA* intron 4 (rs5860181; Taiwan lead)	A	-0.286	0.043	3.729e-11	-0.197	0.060	1.148e-03
	4	89704960	rs356182	*SNCA* 3′ region (rs356182; EUR lead)	A	-0.243	0.047	1.889e-07	-0.031	0.064	6.319e-01
	4	89836121	rs3756059	*SNCA* 5′ region (rs3756059; RBD/PD-RBD reported)	G	-0.398	0.066	1.859e-09	-0.255	0.074	5.886e-04
SNCA *joint_4vars* (+ TW 5′ rs1442145 + RBD 5′ rs3756059)	4	89759478	rs5860181	*SNCA* intron 4 (rs5860181; Taiwan lead)	A	-0.286	0.043	3.729e-11	-0.242	0.063	1.159e-04
	4	89704960	rs356182	SNCA 3′ region (rs356182; EUR lead)	A	-0.243	0.047	1.889e-07	-0.030	0.064	6.370e-01
	4	89815772	rs1442145	*SNCA* 5′ region (rs1442145; Taiwan 5′ signal)	G	-0.182	0.046	7.657e-05	-0.152	0.057	7.157e-03
	4	89836121	rs3756059	*SNCA* 5′ region (rs3756059; RBD/PD-RBD reported)	G	-0.398	0.066	1.859e-09	-0.114	0.091	2.092e-01

joint_2vars (*SCARB2* locus)	4	76217199	rs76591264	*SCARB2/FAM47E* locus (rs76591264; Taiwan signal)	G	0.184	0.046	0.0000692	0.138	0.052	0.007585
	4	76213633	rs11547135	*SCARB2* locus (rs11547135; EUR lead)	C	-0.167	0.047	0.000355	-0.105	0.052	0.0436

Supplementary Table S5. Colocalization of GWAS signals with eQTLs for population-enriched loci

Colocalization was performed using the R "coloc" framework with publicly available whole-blood eQTL summary statistics from **eQTLGen** and brain cortex eQTL summary statistics from **metaBrain (EAS subset)**. For *SNCA*, the locus window was **chr4: 88.7–90.8 Mb**; for other loci, we tested **±500 kb around the lead GWAS variant**. Posterior probabilities are shown for **PP.H3** (distinct causal variants) and **PP.H4** (shared causal variants).

Locus / Gene	Lead variant or window	eQTLGen (whole blood) PP.H3	eQTLGen (whole blood) PP.H4	metaBrain EAS (cortex) PP.H3	metaBrain EAS (cortex) PP.H4
**SNCA**	chr4: 88.7–90.8 Mb	0.33	0.67	0.007	0.03
**PPARGC1A**	rs609501	0.64	0.08	1.8e−5	0.003
**GALNT13**	rs10186542	NA	NA	0.004	0.03
**VPS13C**	rs12900645	0.66	0.05	0.02	0.04
**SCARB2/FAM47E (THAP6 eQTL)**	rs76591264	0.353	0.121	NA	NA
**SCARB2/FAM47E (SCARB2 eQTL)**	rs76591264	0.400	0.014	0.02	0.02

Supplementary Table S6. Look-up of published PD risk variants in the Taiwanese cohort

This table lists PD-associated variants reported by **Nalls et al. (2019)** and **Foo et al. (2020)** ("source") and their corresponding **allele frequency and association results** in our Taiwanese GWAS (Taiwan A1_FREQ, OR/logOR, and P, when available). The rightmost column ("Original effect weight/logOR") reports the **published logOR** from the source study/PRS weights. **NA** indicates variants not available or not reliable after imputation/QC, or monomorphic/near-monomorphic in our cohort. EAS and EUR allele frequencies are provided for reference.

source	rsID	Nearest Gene	chr	position hg38	A1	REF	(Taiwan) A1_FREQ	(Taiwan) OR	(Taiwan) logOR	(Taiwan) P	EAS Frequency	EUR Frequency	Original effect weight/ logOR
Nalls 2019	rs114138760	*PMVK/ GBA1*	1	154925709	NA	NA	NA	NA	NA	NA	None	0.01	0.28
Nalls 2019	rs35749011	*GBA1*	1	155162560	NA	NA	NA	NA	NA	NA	None	0.02	0.61
Nalls 2019	rs76763715	*GBA1*	1	155235843	NA	NA	NA	NA	NA	NA	None	0.00	-0.75
Nalls 2019	rs6658353	*None*	1	161499264	C	G	0.30	1.05	0.05	0.33	0.22	0.51	0.07
Nalls 2019	rs11578699	*None*	1	171750629	T	C	0.18	0.96	-0.04	0.47	0.16	0.17	-0.07
Nalls 2019	rs823118	*None*	1	205754444	C	T	0.40	0.86	-0.15	0.00	0.51	0.53	0.11
Nalls 2019	rs11557080	*RAB29*	1	205768611	G	A	0.40	0.86	-0.16	0.00	0.51	0.13	0.13
Nalls 2019	rs4653767	*ITPKB*	1	226728377	C	T	0.27	0.90	-0.10	0.03	0.26	0.31	0.08
Nalls 2019	rs10797576	*SIPA1L2*	1	232528865	T	C	0.14	1.02	0.02	0.72	0.15	0.14	0.11
Nalls 2019	rs76116224	*KCNS3*	2	17966582	NA	NA	NA	NA	NA	NA	0.00	0.09	0.11
Nalls 2019	rs2042477	*KCNIP3*	2	95335195	A	T	0.44	1.05	0.04	0.30	0.59	0.76	-0.07
Nalls 2019	rs11683001	*MAP4K4*	2	101780501	A	T	0.48	1.03	0.03	0.56	0.49	0.35	0.07

Nalls 2019	rs57891859	*TMEM163*	2	134707046	A	G	0.25	1.02	0.02	0.70	0.76	0.31	0.08
Nalls 2019	rs1474055	*None*	2	168253884	T	C	0.34	1.05	0.05	0.30	0.33	0.12	0.18
Nalls 2019	rs73038319	*TBC1D5*	3	18320267	NA	NA	NA	NA	NA	NA	None	0.03	-0.17
Nalls 2019	rs6808178	*AC098650.1*	3	28664199	T	C	0.20	1.11	0.10	0.06	0.78	0.63	0.07
Nalls 2019	rs6808178	*RBMS3*	3	28664199	T	C	0.20	1.11	0.10	0.06	0.78	0.63	0.07
Nalls 2019	rs12497850	*IP6K2*	3	48711556	G	T	0.04	0.90	-0.10	0.37	0.95	0.66	0.06
Nalls 2019	rs55961674	*KPNA1*	3	122478045	T	C	0.13	0.99	-0.01	0.86	0.11	0.17	0.09
Nalls 2019	rs11707416	*MED12L*	3	151391177	A	T	0.14	1.01	0.01	0.83	0.14	0.38	-0.06
Nalls 2019	rs1450522	*SPTSSB*	3	161359842	A	G	0.44	1.01	0.01	0.86	0.54	0.31	-0.06
Nalls 2019	rs10513789	*MCCC1*	3	183042285	T	G	0.43	1.30	0.27	0.00	0.57	0.20	0.15
Nalls 2019	rs873786	*GAK*	4	931588	NA	NA	NA	NA	NA	NA	0.00	0.10	-0.17
Nalls 2019	rs34311866	*TMEM175*	4	958159	C	T	0.13	1.16	0.15	0.02	0.13	0.19	-0.21
Nalls 2019	rs4698412	*BST1*	4	15735725	A	G	0.40	1.14	0.13	0.00	0.38	0.56	0.10
Nalls 2019	rs34025766	*LCORL*	4	17967188	A	T	0.13	1.05	0.05	0.40	0.16	0.13	-0.08
Nalls 2019	rs6825004	*SCARB2*	4	76189212	G	C	0.39	0.97	-0.03	0.54	0.36	0.31	0.06
Nalls 2019	rs4101061	*SCARB2/FAM47E*	4	76226816	A	G	0.23	1.05	0.05	0.31	0.78	0.28	-0.09
Nalls 2019	rs6854006	*FAM47E-STBD1*	4	76276901	T	C	0.09	1.17	0.16	0.03	0.08	0.39	-0.09

Nalls 2019	rs356182	*SNCA*	4	89704960	A	G	0.30	0.78	-0.24	0.00	0.37	0.64	-0.28
Nalls 2019	rs5019538	*SNCA*	4	89715479	NA	NA	NA	NA	NA	NA	1.00	0.68	-0.16
Nalls 2019	rs13117519	*None*	4	113447909	T	C	0.09	1.15	0.14	0.06	0.08	0.18	0.09
Nalls 2019	rs62333164	*NA*	4	169662006	A	G	0.06	0.83	-0.18	0.04	NA	NA	-0.06
Nalls 2019	rs1867598	*ELOVL7*	5	60842132	G	A	0.06	1.11	0.10	0.26	0.05	0.11	-0.16
Nalls 2019	rs26431	*PAM*	5	103030090	G	C	0.44	0.97	-0.03	0.47	0.55	0.72	0.06
Nalls 2019	rs11950533	*AC006077.1*	5	134863415	A	C	0.24	1.00	0.00	0.96	0.31	0.14	-0.09
Nalls 2019	rs4140646	*None*	6	27771022	A	G	0.22	1.12	0.11	0.06	0.18	0.22	0.08
Nalls 2019	rs9261484	*TRIM40*	6	30140906	T	C	0.31	1.00	0.00	1.00	0.26	0.26	-0.06
Nalls 2019	rs112485576	*NA*	6	32610995	A	C	0.08	0.99	-0.01	0.96	NA	NA	-0.17
Nalls 2019	rs12528068	*None*	6	71778059	T	C	0.08	1.12	0.11	0.17	0.09	0.26	0.07
Nalls 2019	rs997368	*None*	6	111922088	G	A	0.37	0.92	-0.08	0.08	0.36	0.20	0.07
Nalls 2019	rs75859381	*None*	6	132889222	C	T	0.04	1.26	0.23	0.05	0.02	0.03	-0.22
Nalls 2019	rs199351	*GPNMB*	7	23260430	C	A	0.33	0.96	-0.04	0.37	0.35	0.38	0.10
Nalls 2019	rs76949143	*AC006001.3/AC008267.3*	7	66544864	A	T	0.14	0.88	-0.13	0.04	0.17	0.06	-0.14
Nalls 2019	rs1293298	*CTSB*	8	11854934	NA	NA	NA	NA	NA	NA	0.00	0.26	0.09
Nalls 2019	rs620513	*AC011586.2*	8	16840084	T	G	0.42	0.90	-0.11	0.01	0.42	0.29	-0.09

Nalls 2019	rs2280104	*BIN3*	8	22668467	T	C	0.17	0.95	-0.05	0.35	0.80	0.63	0.06
Nalls 2019	rs2086641	*FAM49B*	8	129889663	C	T	0.50	1.05	0.05	0.24	0.52	0.28	-0.06
Nalls 2019	rs13294100	*SH3GL2*	9	17579692	G	T	0.47	1.08	0.08	0.07	0.48	0.64	-0.09
Nalls 2019	rs10756907	*SH3GL2*	9	17727067	G	A	0.39	1.04	0.04	0.32	0.37	0.23	-0.09
Nalls 2019	rs6476434	*UBAP2*	9	34046393	C	T	0.16	1.13	0.12	0.04	0.83	0.75	-0.06
Nalls 2019	rs896435	*ITGA8*	10	15515407	T	C	0.44	0.97	-0.03	0.47	0.43	0.71	0.07
Nalls 2019	rs10748818	*GBF1*	10	102255522	G	A	0.36	1.15	0.14	0.00	0.37	0.12	-0.08
Nalls 2019	rs72840788	*BAG3*	10	119656173	NA	NA	NA	NA	NA	NA	0.00	0.22	0.08
Nalls 2019	rs117896735	*INPP5F*	10	119776815	NA	NA	NA	NA	NA	NA	None	0.01	0.44
Nalls 2019	rs7938782	*RNF141*	11	10537230	G	A	0.16	0.93	-0.07	0.22	0.14	0.15	0.09
Nalls 2019	rs12283611	*DLG2*	11	83776234	A	C	0.25	1.07	0.06	0.20	0.28	0.43	-0.06
Nalls 2019	rs3802920	*IGSF9B*	11	133917106	T	G	0.15	0.96	-0.04	0.46	0.15	0.18	0.11
Nalls 2019	rs76904798	*LRRK2*	12	40220632	T	C	0.05	1.09	0.08	0.39	0.06	0.13	0.14
Nalls 2019	rs34637584	*LRRK2*	12	40340400	NA	NA	NA	NA	NA	NA	None	None	2.43
Nalls 2019	rs7134559	*None*	12	46025303	C	T	0.40	1.12	0.11	0.01	0.56	0.41	-0.05
Nalls 2019	rs10847864	*HIP1R*	12	122842051	G	T	0.45	0.96	-0.04	0.35	0.53	0.34	0.15
Nalls 2019	rs11610045	*None*	12	132487182	A	G	0.13	1.09	0.08	0.20	0.11	0.45	0.06
Nalls 2019	rs9568188	*CAB39L*	13	49353596	T	C	0.35	1.01	0.01	0.75	0.63	0.26	0.06
Nalls 2019	rs4771268	*None*	13	97212767	T	C	0.40	1.05	0.05	0.25	0.65	0.79	0.07

Nalls 2019	rs12147950	*MIPOL1*	14	37520065	T	C	0.46	0.99	-0.01	0.75	0.51	0.56	-0.05
Nalls 2019	rs11158026	*GCH1*	14	54882151	C	T	0.46	1.23	0.21	0.00	0.55	0.31	-0.08
Nalls 2019	rs3742785	*RPS6KL1*	14	74906331	C	A	0.20	0.98	-0.02	0.76	0.20	0.23	0.07
Nalls 2019	rs979812	*None*	14	87997920	G	T	0.35	0.98	-0.02	0.67	0.61	0.42	0.06
Nalls 2019	rs2251086	*AC018618.1*	15	61705186	T	C	0.15	0.88	-0.13	0.03	0.81	0.84	-0.12
Nalls 2019	rs6497339	*SYT17*	16	19266171	A	T	0.01	1.27	0.24	0.19	0.99	0.54	0.06
Nalls 2019	rs2904880	*CD19/RABEP2*	16	28933075	C	G	0.08	1.06	0.06	0.46	0.90	0.73	-0.07
Nalls 2019	rs11150601	*SETD1A*	16	30966478	G	A	0.08	0.82	-0.20	0.01	0.90	0.64	0.09
Nalls 2019	rs6500328	*NOD2*	16	50702745	G	A	0.20	1.00	0.00	0.96	0.18	0.43	0.06
Nalls 2019	rs3104783	*CASC16*	16	52602330	C	A	0.21	0.91	-0.09	0.08	0.80	0.43	0.07
Nalls 2019	rs10221156	*PHBP21*	16	52935514	A	G	0.04	1.09	0.08	0.47	0.03	0.08	-0.12
Nalls 2019	rs12600861	*CHRNB1*	17	7452302	C	A	0.23	1.04	0.04	0.42	0.22	0.34	-0.06
Nalls 2019	rs12951632	*RETREG3*	17	42588995	C	T	0.44	0.94	-0.06	0.14	0.45	0.27	0.06
Nalls 2019	rs2269906	*UBTF*	17	44216969	A	C	0.23	1.06	0.06	0.25	0.75	0.32	0.06
Nalls 2019	rs850738	*FAM171A2*	17	44357262	G	A	0.48	1.05	0.05	0.29	0.52	0.41	-0.07
Nalls 2019	rs62053943	*LINC02210-CRHR1*	17	45666837	NA	NA	NA	NA	NA	NA	0.00	0.17	-0.27

Nalls 2019	rs117615688	*LINC02210-CRHR1*	17	45720942	NA	NA	NA	NA	NA	NA	None	0.07	-0.23
Nalls 2019	rs11658976	*WNT3*	17	46789439	A	G	0.38	0.99	-0.01	0.81	0.40	0.62	-0.06
Nalls 2019	rs61169879	*BRIP1*	17	61840005	C	T	0.44	0.98	-0.02	0.62	0.56	0.15	0.08
Nalls 2019	rs666463	*DNAH17*	17	78429399	T	A	0.04	0.94	-0.06	0.60	0.02	0.18	0.08
Nalls 2019	rs1941685	*ASXL3*	18	33724354	G	T	0.10	0.95	-0.05	0.45	0.88	0.51	0.05
Nalls 2019	rs12456492	*RIT2*	18	43093415	G	A	0.40	1.13	0.12	0.01	0.36	0.34	-0.10
Nalls 2019	rs8087969	*None*	18	51157219	G	T	0.28	0.94	-0.07	0.17	0.24	0.44	-0.06
Nalls 2019	rs55818311	*SPPL2B/AC005258.1*	19	2341049	T	C	0.30	0.98	-0.02	0.68	0.34	0.67	-0.07
Nalls 2019	rs77351827	*CRLS1*	20	6025395	NA	NA	NA	NA	NA	NA	0.00	0.13	0.08
Nalls 2019	rs2248244	*DYRK1A*	21	37480059	A	G	0.38	1.11	0.11	0.02	0.37	0.26	0.07
source	rsID	Nearest Gene	chr	position hg38	A1	REF	A1_FREQ	OR	logOR	P	EAS Frequency	EUR Frequency	Original effect weight/ logOR
Foo 2020	rs6679073	*PARK16*	1	205787356	A	C	0.41	1.13	0.12	4.9E-03	0.50	0.25	0.21
Foo 2020	rs16846351	*ITPKB*	1	226659011	G	C	0.07	1.29	0.25	2.5E-03	0.07	None	0.29
Foo 2020	rs12278023	*DLG2*	11	83799074	C	T	0.49	0.93	-0.07	7.8E-02	0.54	0.45	-0.13
Foo 2020	rs141336855	*LRRK2*	12	39993947	T	G	0.03	1.58	0.46	1.9E-04	0.02	None	0.69
Foo 2020	rs4130047	*RIT2*	18	43098270	C	T	0.39	1.13	0.12	5.9E-03	0.36	0.34	0.13

Foo 2020	rs2292056	*MCCC1*	3	183017423	G	T	0.43	0.76	-0.27	8.5E-10	0.57	0.20	-0.19
Foo 2020	rs3816248	*SCARB2/FAM47E*	4	76179915	C	T	0.36	0.97	-0.04	4.3E-01	0.33	0.13	-0.14
Foo 2020	rs6826785	*SNCA*	4	89761323	C	T	0.43	1.32	0.28	1.6E-10	0.54	0.07	0.29
Foo 2020	rs246814	*SV2C*	5	76303383	T	C	0.09	1.11	0.11	1.5E-01	0.09	0.08	0.22
Foo 2020	rs1887316	*FYN*	6	111830249	A	G	0.12	0.82	-0.20	1.8E-03	0.12	0.11	-0.19
Foo 2020	rs9638616	*WBSCR17/GALNT17*	7	71285507	T	G	0.46	0.99	-0.01	8.7E-01	0.48	0.32	0.13

Supplementary Table 8. Polygenic Risk Score (PRS) variants and weights
This table summarizes the variants and effect-size weights used to construct the PRS models evaluated in this study.

Supplementary Table 8A. PRS model specifications and performance
Number of variants and AUC performance for the four PRS models evaluated.

Supplementary Table 8B. Variant list and weights for the reweighted PRS (EUR + Asian11 + TW4)
This table provides the full variant list and weights for the combined model comprising 76 European variants and 11 East Asian–specific variants as reported by Foo et al. (2020), plus four additional variants included to reflect Taiwan-specific genetic architecture. The Taiwan-specific set includes two *LRRK2* exonic variants (p.R1628P and p.G2385R) and two additional variants in/near *VPS13C* and *SCARB2/FAM47E* identified in this study.

Supplementary Table 8C. Variant list and weights for the extended model (Asian11 + TW4 + GCH1)
This table provides the variant list and weights for a model consisting of the 11 East Asian–specific variants (Foo et al., 2020), the four Taiwan-specific variants described above, and an additional *GCH1* variant that showed evidence of association in the Taiwanese cohort.

Supplementary Table 8A.

	SNP number	AUC
PRS-EUR (Nalls)	76	0.59 (0.55-0.63)
PRS-EUR-Asia	87	0.61 (0.568-0.651)
PRS-TW-91SNVs	91	0.62 (0.579-0.662)
PRS-TW-16SNVs	16	0.61 (0.567-0.650)

Supplementary Table 8B.

rsID	chromosome	position	effect_allele	weight
rs6658353	1	161499264	G	-0.022
rs11578699	1	171750629	C	0.013
rs823118	1	205754444	T	-0.444
rs11557080	1	205768611	A	0.735
rs6679073	1	205787356	A	0.038
rs16846351	1	226659011	C	-0.023
rs4653767	1	226728377	T	-0.061
rs10797576	1	232528865	C	-0.034

rs896435	10	15515407	C	-0.016
rs10748818	10	102255522	A	-0.02
rs7938782	11	10537230	A	-0.049
rs12283611	11	83776234	C	0.207
rs12278023	11	83799074	C	0.028
rs3802920	11	133917106	G	-0.016
rs141336855	12	39993947	G	-0.012
rs76904798	12	40220632	C	-0.277
rs33949390	12	40320043	G	-0.153
rs34778348	12	40363526	G	-0.103
rs7134559	12	46025303	T	-0.072
rs10847864	12	122842051	T	-0.178
rs11610045	12	132487182	G	-0.168
rs9568188	13	49353596	C	-0.142
rs4771268	13	97212767	C	0.115
rs12147950	14	37520065	C	-0.127
rs11158026	14	54882151	T	0.147
rs3742785	14	74906331	A	-0.103
rs979812	14	87997920	T	0.001
rs2251086	15	61705186	C	0.015
rs12900645	15	61994009	A	-0.092
rs6497339	16	19266171	T	0.03
rs2904880	16	28933075	G	0.02
rs11150601	16	30966478	A	-0.16
rs6500328	16	50702745	A	0.008
rs3104783	16	52602330	A	-0.252
rs10221156	16	52935514	G	0.041
rs12600861	17	7452302	A	0.089
rs12951632	17	42588995	T	0.087
rs2269906	17	44216969	C	0.018
rs850738	17	44357262	A	-0.078

rs11658976	17	46789439	G	-0.066
rs61169879	17	61840005	T	-0.041
rs666463	17	78429399	A	-0.067
rs1941685	18	33724354	T	0.045
rs12456492	18	43093415	A	-0.13
rs4130047	18	43098270	T	0.071
rs8087969	18	51157219	T	-0.156
rs55818311	19	2341049	C	0.091
rs2042477	2	95335195	T	-0.161
rs11683001	2	101780501	T	-0.114
rs57891859	2	134707046	G	0.054
rs1474055	2	168253884	C	-0.111
rs2248244	21	37480059	G	-0.009
rs6808178	3	28664199	C	-0.039
rs12497850	3	48711556	T	0.035
rs55961674	3	122478045	C	-0.211
rs11707416	3	151391177	T	-0.013
rs1450522	3	161359842	G	-0.008
rs2292056	3	183017423	G	0.12
rs10513789	3	183042285	G	-0.191
rs34311866	4	958159	T	0.007
rs4698412	4	15735725	G	0.263
rs34025766	4	17967188	T	0.007
rs3816248	4	76179915	T	0.089
rs6825004	4	76189212	C	0.011
rs76591264	4	76217199	C	-0.04
rs4101061	4	76226816	G	0.064
rs6854006	4	76276901	C	-0.08
rs356182	4	89704960	G	-0.032
rs6826785	4	89761323	C	0.012
rs13117519	4	113447909	C	-0.026

rs62333164	4	169662006	G	-0.019
rs1867598	5	60842132	A	0.015
rs246814	5	76303383	C	-0.374
rs26431	5	103030090	C	0.037
rs11950533	5	134863415	C	0.018
rs4140646	6	27771022	G	-0.121
rs9261484	6	30140906	C	-0.15
rs112485576	6	32610995	C	-0.232
rs12528068	6	71778059	C	-0.143
rs1887316	6	111830249	G	-0.135
rs997368	6	111922088	A	0.277
rs75859381	6	132889222	T	-0.023
rs199351	7	23260430	A	0.17
rs76949143	7	66544864	T	0.172
rs9638616	7	71285507	G	-0.124
rs620513	8	16840084	G	0.088
rs2280104	8	22668467	C	-0.004
rs2086641	8	129889663	T	-0.378
rs13294100	9	17579692	T	-0.346
rs10756907	9	17727067	A	-0.238
rs6476434	9	34046393	T	0.206

Supplementary Table 8C.

**rsID**	**chromosome**	**position**	**effect_allele**	**weight**
rs6679073	1	205787356	A	0.13
rs16846351	1	226659011	C	-0.235
rs12278023	11	83799074	C	-0.078
rs141336855	12	39993947	G	-0.163
rs33949390	12	40320043	G	-0.097
rs34778348	12	40363526	G	-0.279
rs11158026	14	54882151	T	-0.007

rs12900645	15	61994009	A	0.249
rs4130047	18	43098270	T	-0.119
rs2292056	3	183017423	G	0.091
rs3816248	4	76179915	T	0.008
rs76591264	4	76217199	C	-0.331
rs6826785	4	89761323	C	-0.298
rs246814	5	76303383	C	-0.185
rs1887316	6	111830249	G	0.202
rs9638616	7	71285507	G	-0.215

Supplementary Figure S1. Principal component analysis of the 1000 Genomes reference panel with projection of the Taiwanese cohort (TW, gray). TW samples cluster with the East Asian (EAS, green) superpopulation on PC1–PC2, consistent with East Asian ancestry. PCs were computed on LD-pruned autosomal SNPs in the 1000 Genomes Project; Taiwanese samples were projected without refitting. Other superpopulations are shown for context (AFR, AMR, EUR, SAS).

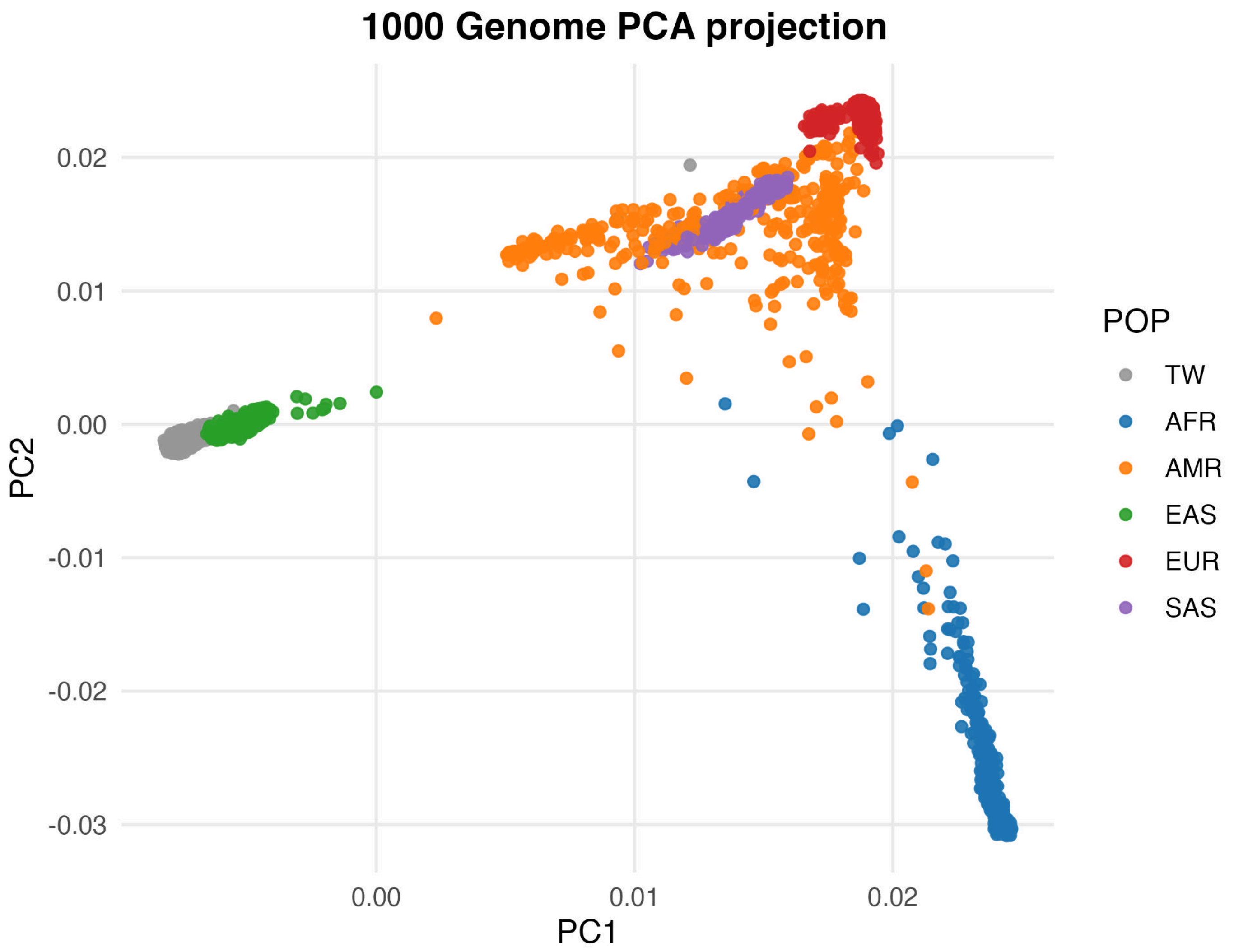

Supplementary Figure S2. Principal component analysis (PCA) for population structure and site effects.

PCA was performed to assess potential genetic stratification between (a) Parkinson's disease (PD) cases and controls and (b–c) recruiting hospitals/sites. (a) PCA colored by case–control status. (b) PCA colored by all six recruiting sites (NTUH, CGMH, TWB, SHH, Landseed, TMU). (c) PCA colored by recruiting sites excluding NTUH. Overall, no obvious clustering/separation was observed by case–control status or by recruiting site, suggesting minimal population stratification and site-specific batch effects. (d-e) Scree plot and variance explained in the top 20 principal components. (f) Manhattan plot based on the GWAS adjusted for top 5 PCs.

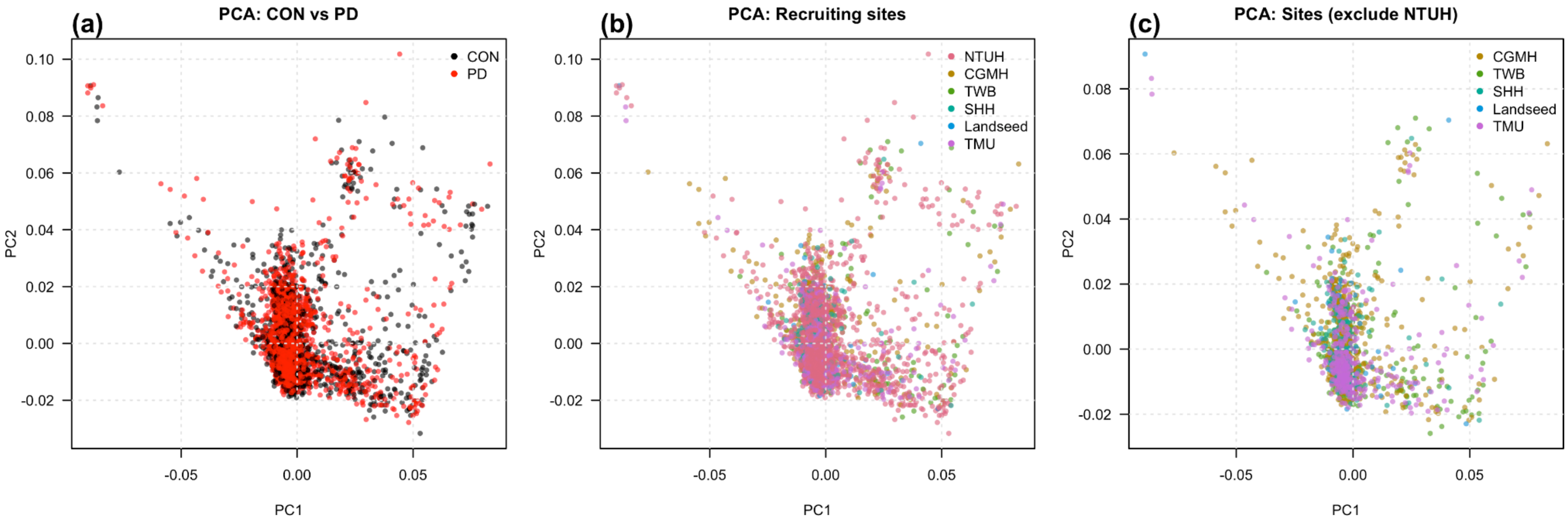

(d-e)

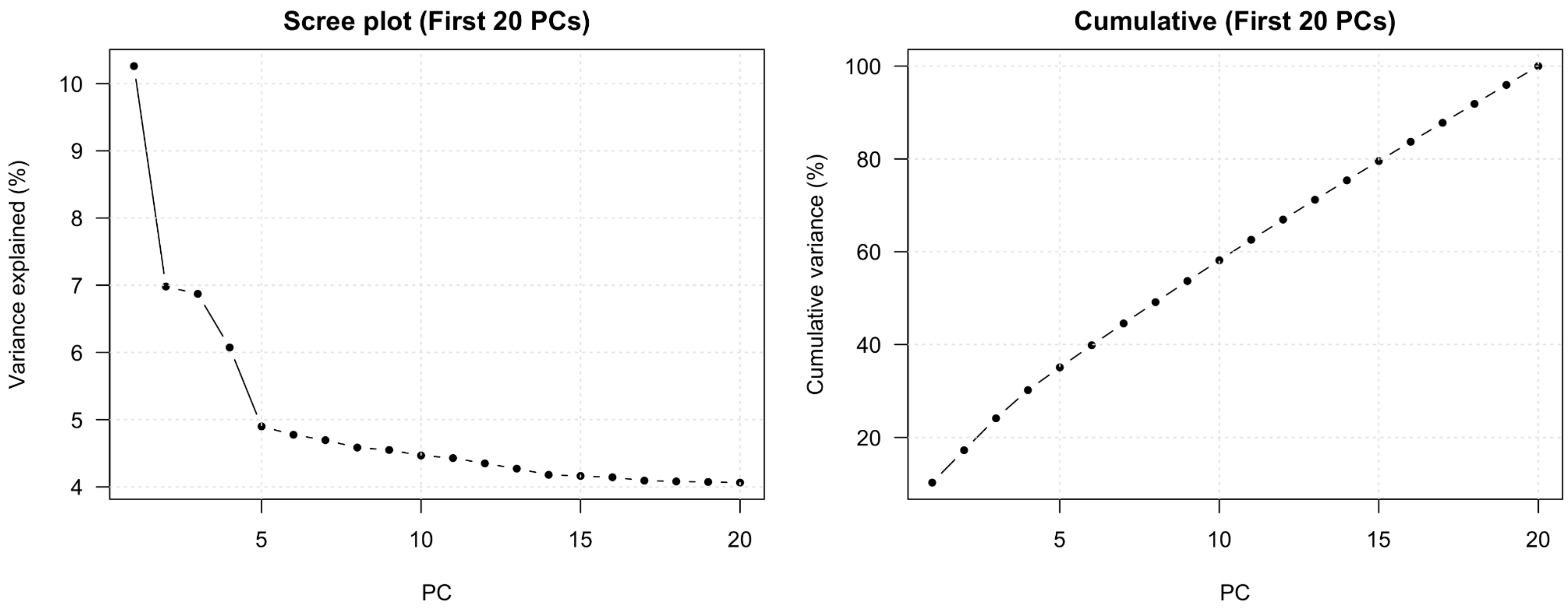
Scree plot (First 20 PCs)
Variance explained (%)
PC
4
5
6
7
8
9
10
5
10
15
20
Cumulative (First 20 PCs)
Cumulative variance (%)
PC
20
40
60
80
100
5
10
15
20

(f)

Supplementary Figure S3. Quantile–quantile (QQ) plot of genome-wide association p-values.

The QQ plot demonstrates excellent calibration of test statistics, with a genomic control inflation factor (λ_{GC} = 1.02). The observed p-value distribution closely follows the expected null distribution (dashed line), indicating minimal residual population stratification or technical artifacts.

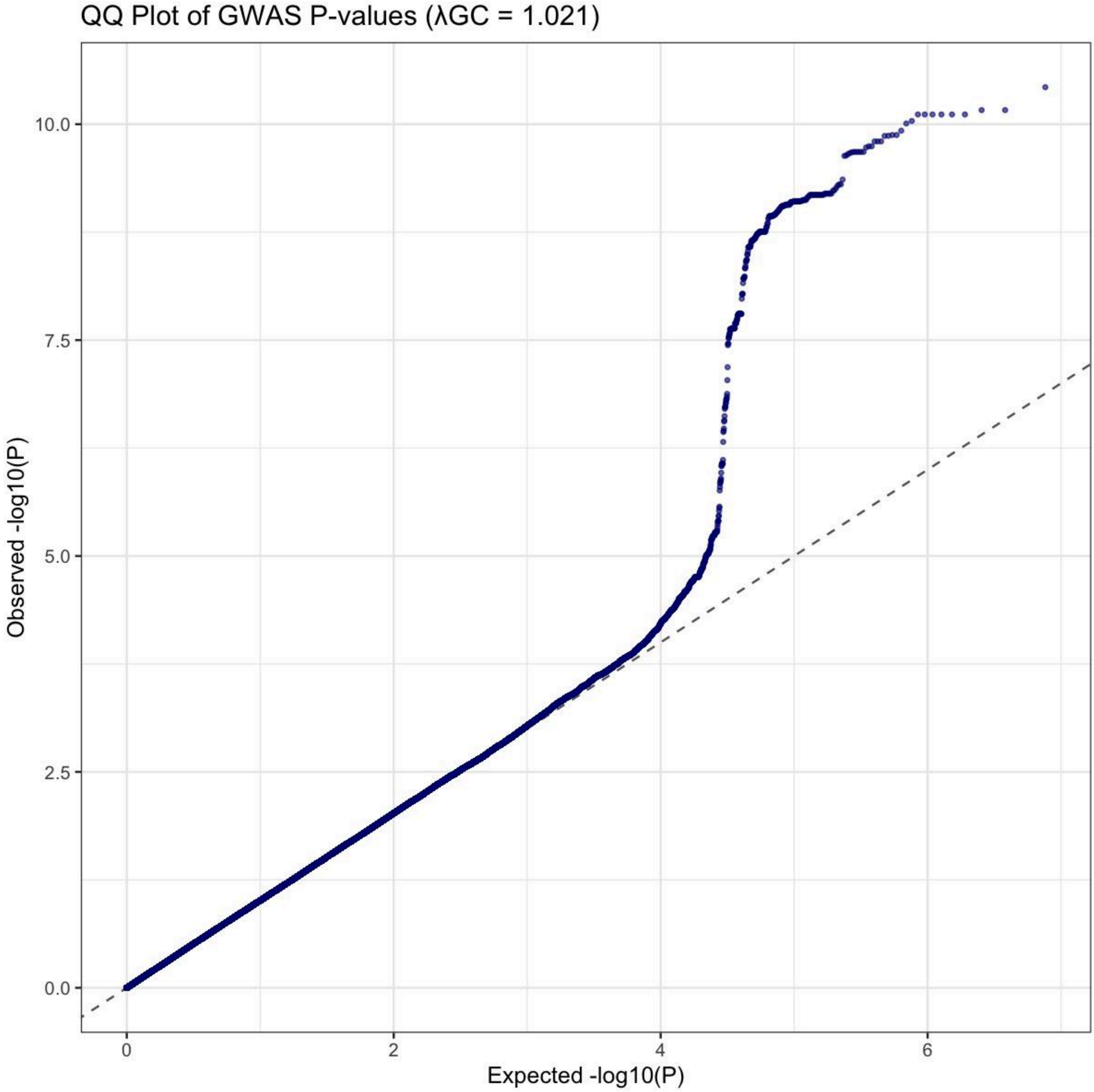

Supplementary Figure S4. Regional association plots for key loci (a–h). LocusZoom-style plots show $-\log_{10}(P)$ by genomic position (hg38) across ±300 kb around each lead SNP. LD is computed from cohort controls; lead variants are highlighted in purple and other variants are colored by r^2 with the lead.

(a) ***MCCC1*** (lead rs34822404, chr3:183,028,420); (b) ***GCH1*** (lead rs3783640, chr14:54,885,803); (c) ***PPARGC1A*** (lead rs2970847, chr4:23,756,312); (d) ***GALNT13*** (lead rs10186542, chr2:154,088,802); (e) ***VPS13C*** (lead rs12900645, chr15:61,994,009), distant from and not in LD with rs2251086 reported by Nalls; rs2251086 lies within *VPS13C*, supporting *VPS13C* as the risk locus; (f) ***SCARB2*** (lead rs76591264, chr4:76,217,199), not in LD with rs4101061 reported by Nalls.

a. ***MCCC1*** (lead rs34822404, chr3:183,028,420).

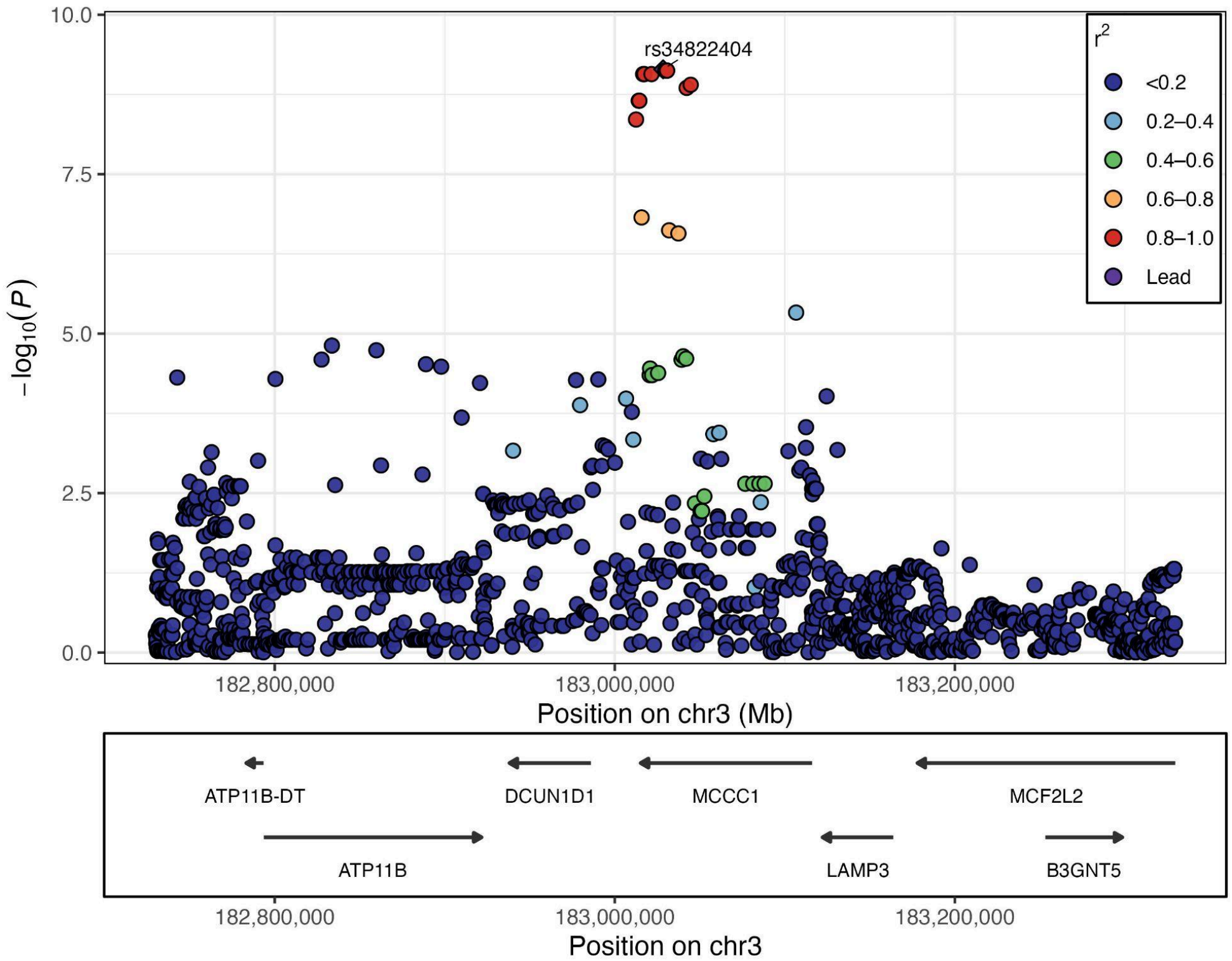

b**. *GCH1*** (lead rs3783640, chr14:54,885,803).

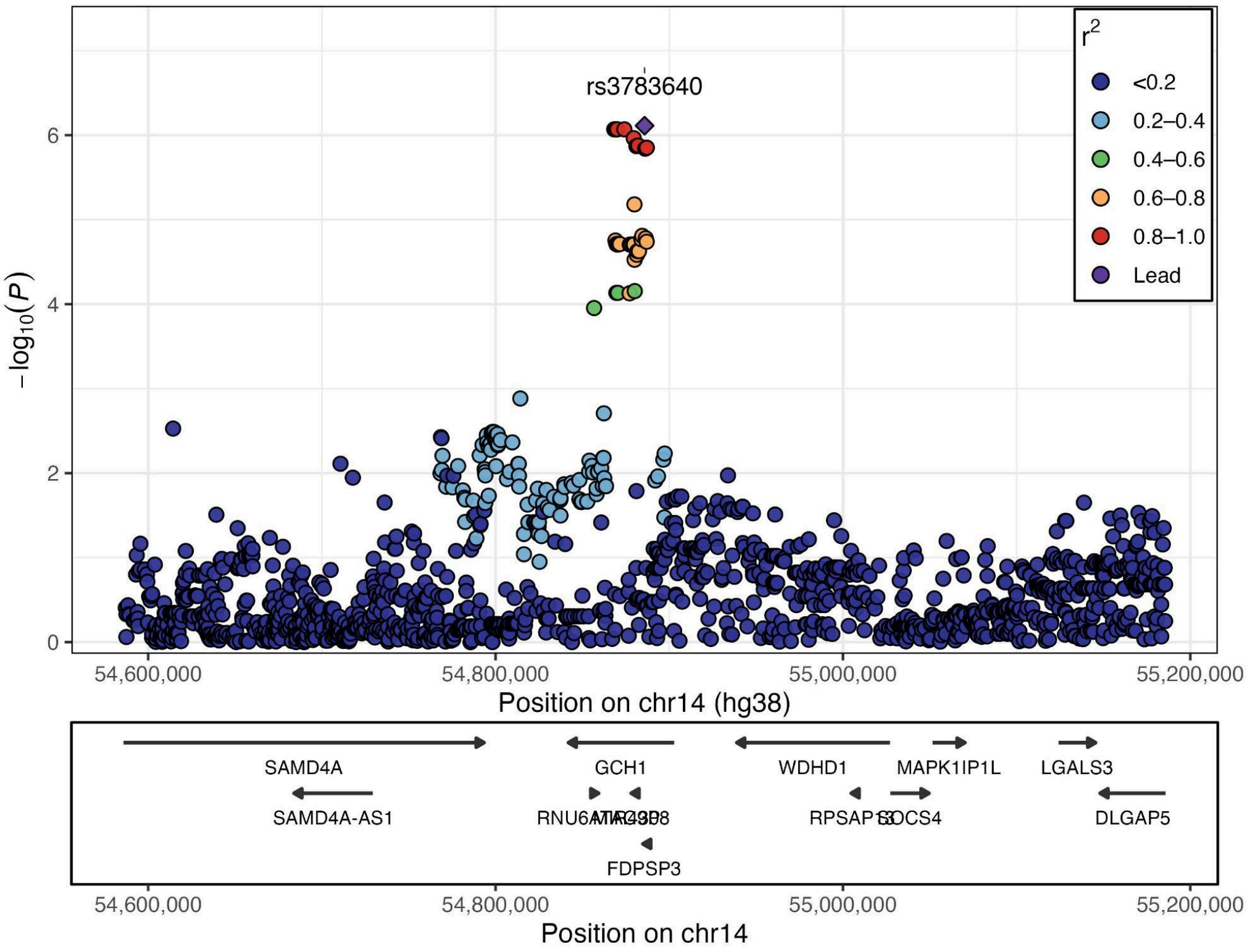

c. *PPARGC1A* (lead rs2970847, chr4:23,756,312).

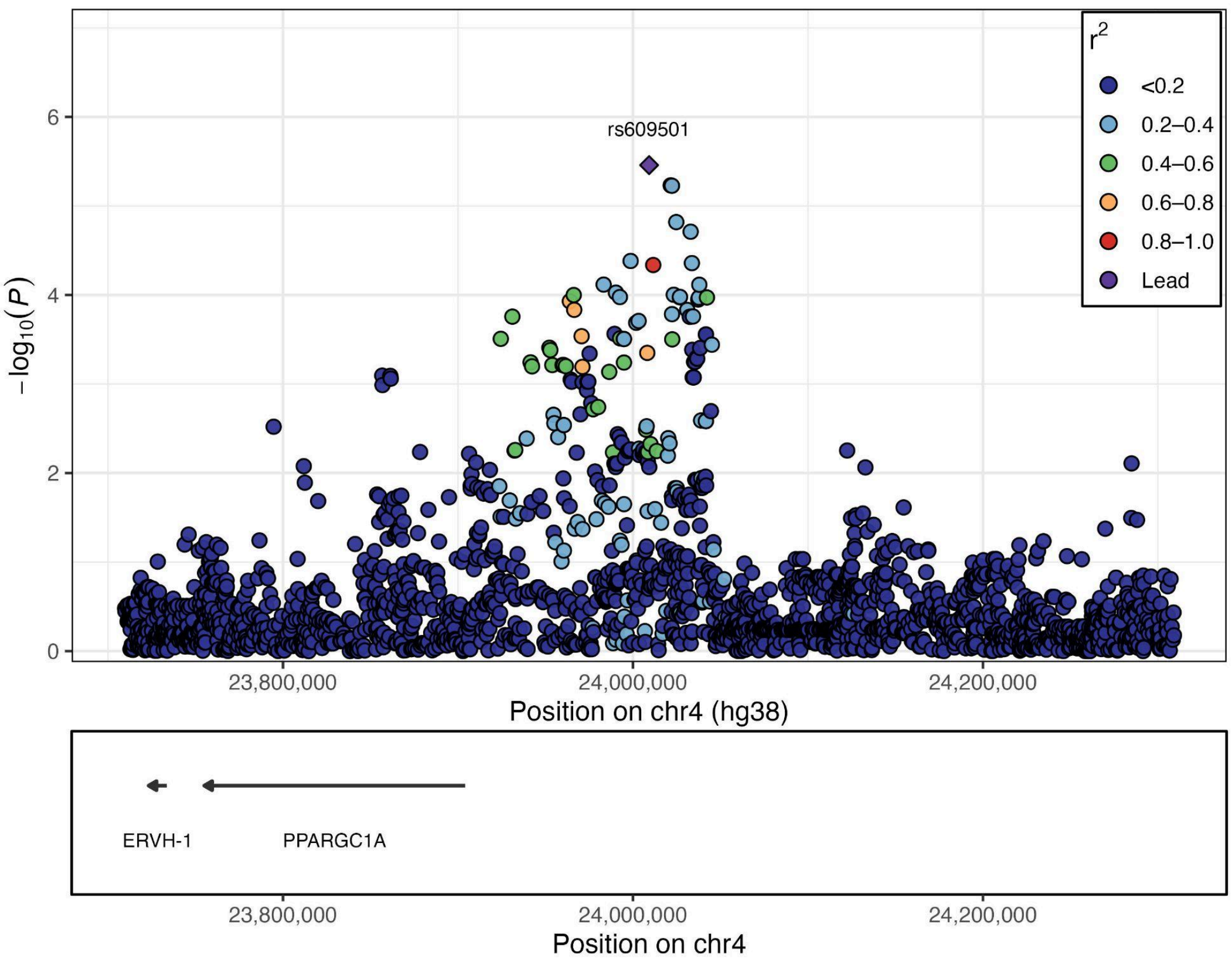

d. ***GALNT13*** (lead rs10186542, chr2:154,088,802).

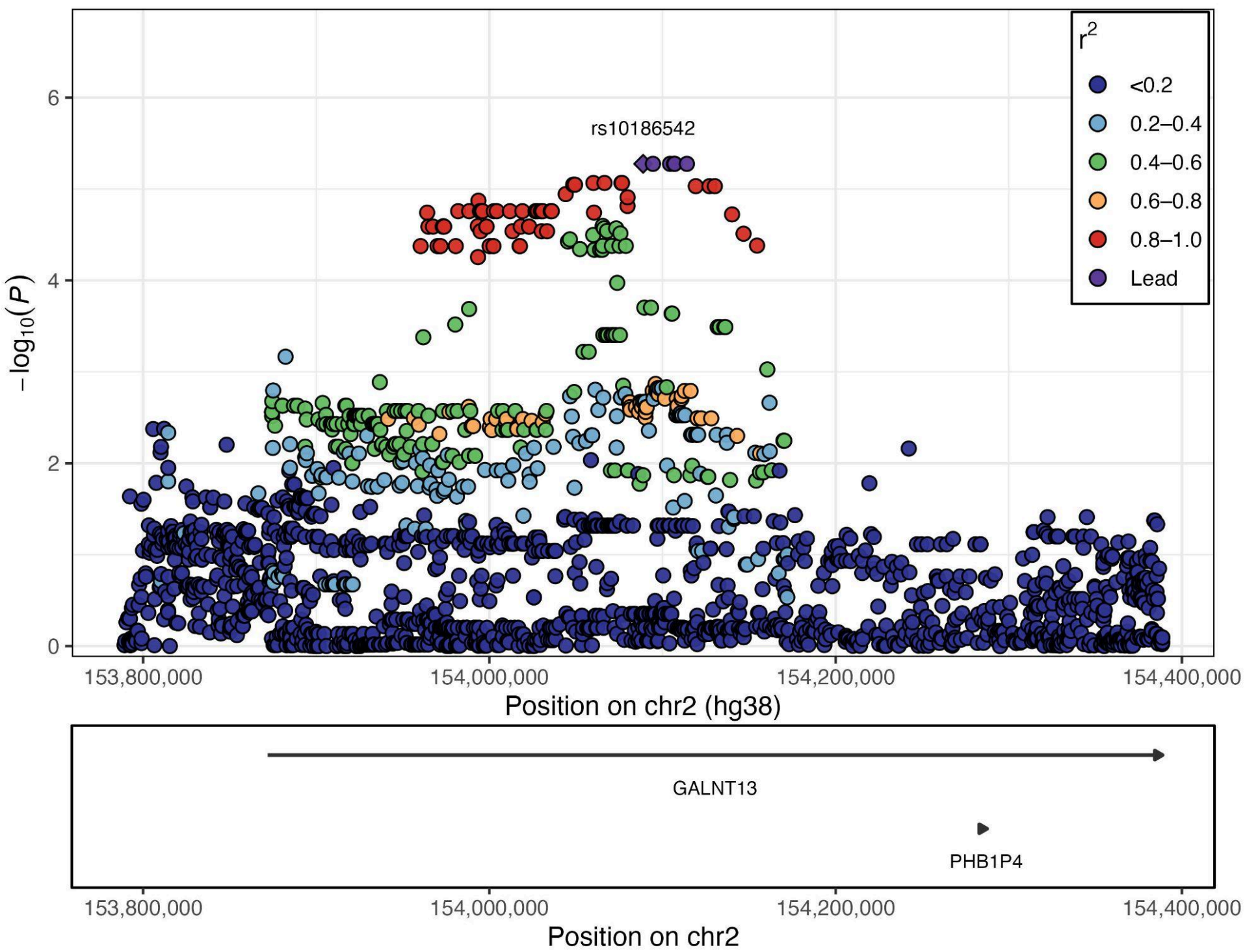

e. ***VPS13C*** (lead rs12900645, chr15:61,994,009). This signal is distant from and not in LD with rs2251086 reported by Nalls et al.; rs12900645 lies within *VPS13C*, supporting *VPS13C* as the risk locus.

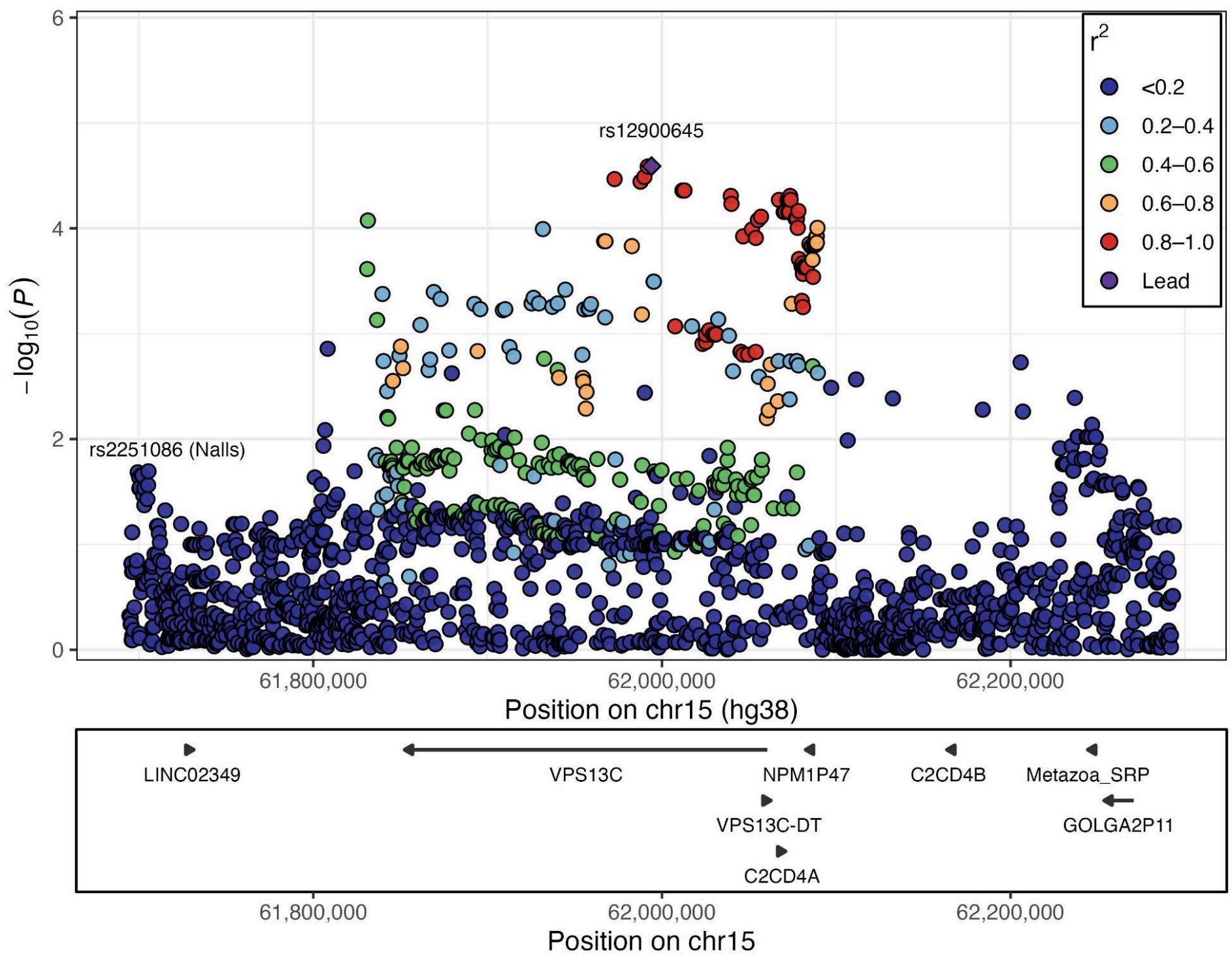

f. *SCARB2* (lead rs76591264, chr4:76,217,199). This signal is not in LD with rs4101061 reported by Nalls et al.

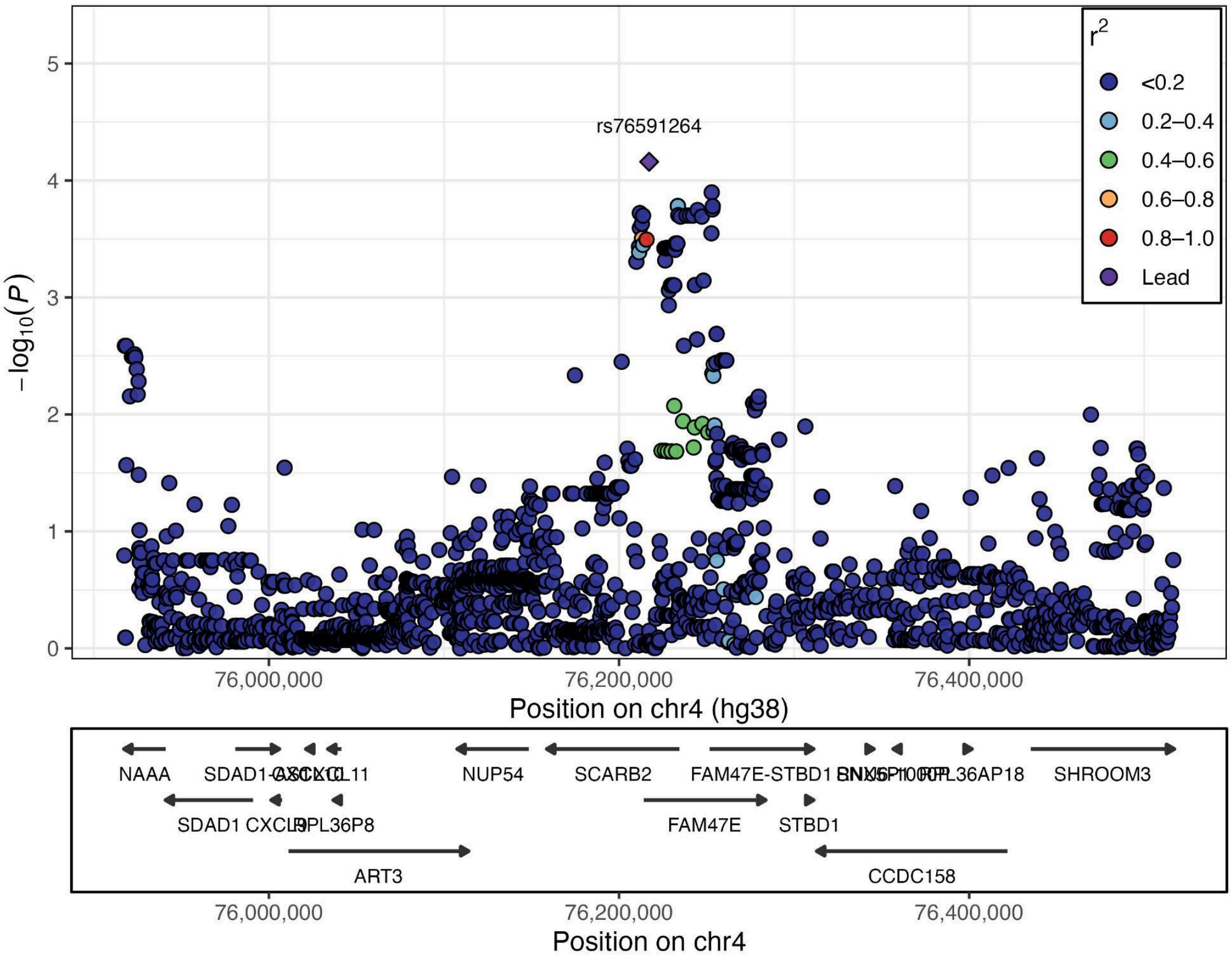

Supplementary Figure S5. Site-stratified forest plots for key loci identified in the Taiwanese PD GWAS.
For each variant, we performed site-stratified association analyses in three recruitment strata—**NTUH+TWB+Landseed**, **CGMH**, and **SHH+TMU**—followed by **fixed-effects inverse-variance meta-analysis** ("Combined"). Squares denote the per-allele odds ratio (OR) estimates from each stratum and the meta-analysis; square size is proportional to the inverse-variance weight. Horizontal lines indicate **95% confidence intervals**. The vertical dashed line marks **OR = 1.0** (no effect). Between-stratum heterogeneity is summarized by **I²** and the Cochran's Q-test P value shown below each panel.

SNCA (intron 4), rs5860181

Cohort	OR (95% CI)
NTUH+TWB+Landseed	0.79 (0.72–0.88)
CGMH	0.66 (0.52–0.82)
SHH+TMU	0.56 (0.40–0.78)
**Combined**	**0.75 (0.69–0.82)**

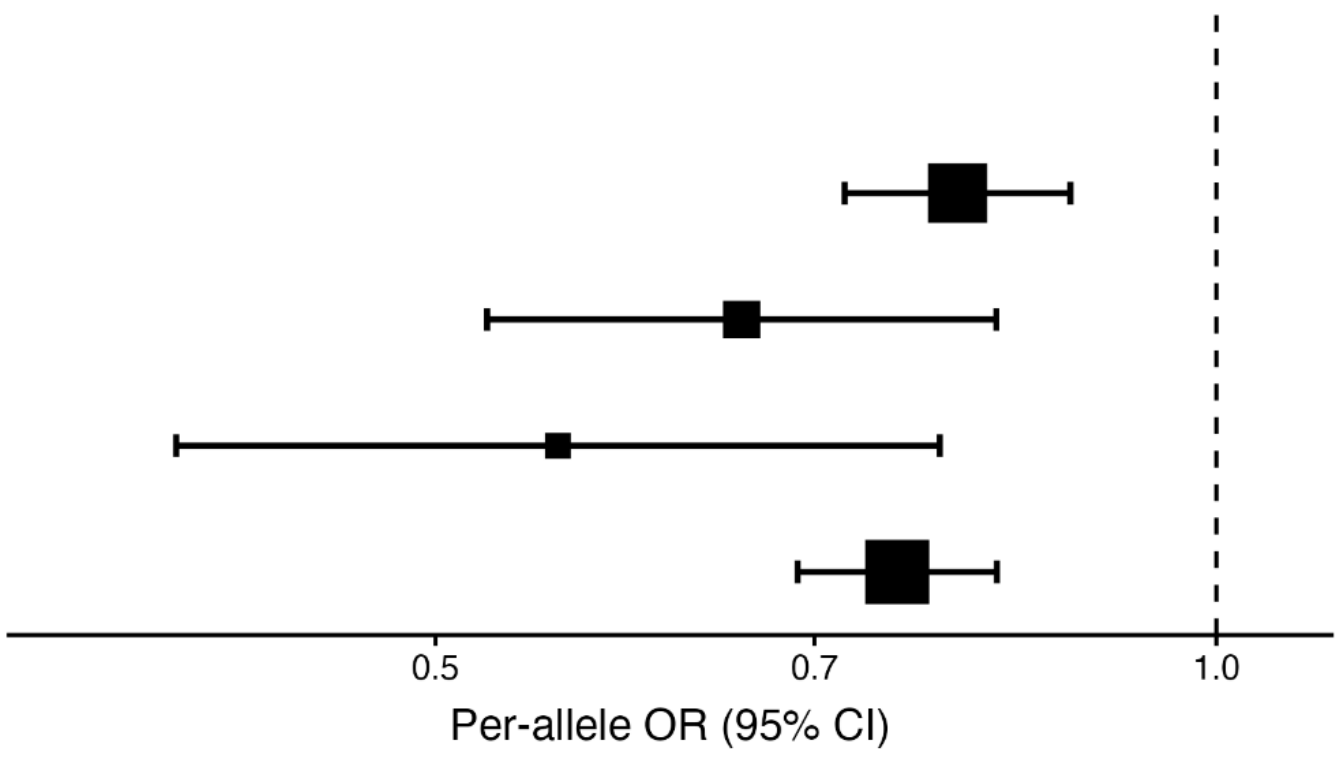

I^2 = 64.0%, P(heterogeneity) = 0.97

SNCA (intron 4), rs3775427

Cohort	OR (95% CI)
NTUH+TWB+Landseed	0.79 (0.72–0.88)
CGMH	0.65 (0.52–0.82)
SHH+TMU	0.57 (0.40–0.80)
**Combined**	**0.75 (0.69–0.82)**

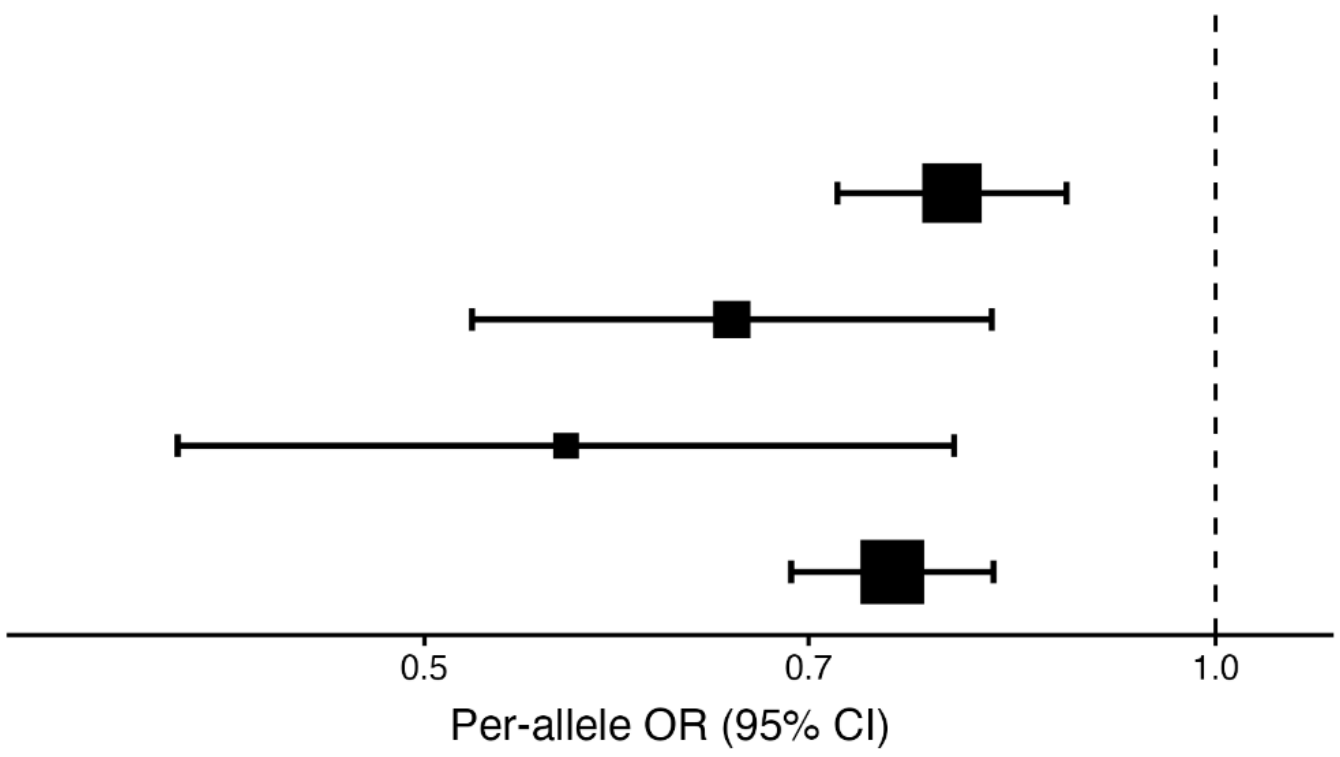

I^2 = 61.7%, P(heterogeneity) = 0.96

SNCA (3' UTR), rs356182

Cohort	OR (95% CI)
NTUH+TWB+Landseed	0.82 (0.74–0.91)
CGMH	0.75 (0.59–0.95)
SHH+TMU	0.50 (0.34–0.75)
**Combined**	**0.79 (0.71–0.86)**

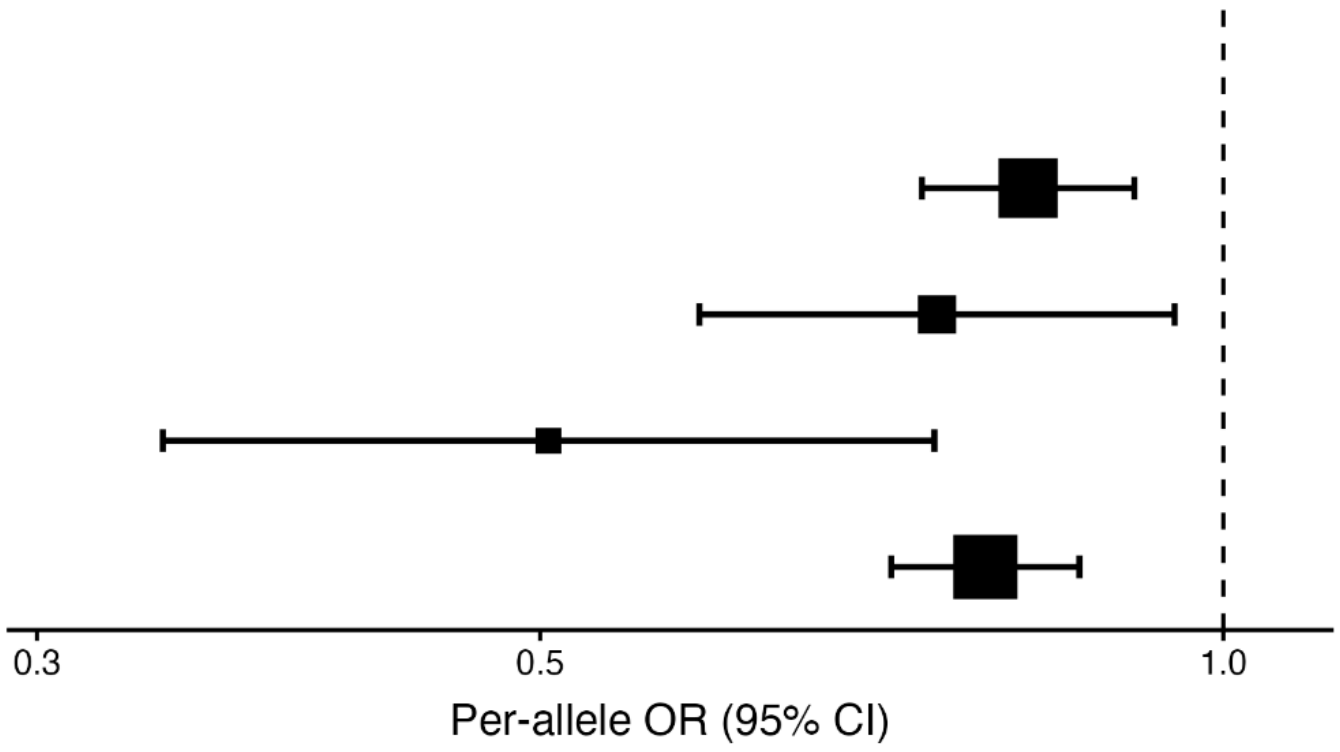

I² = 65.0%, P(heterogeneity) = 0.97

SNCA (5' UTR), rs1442145

Cohort	OR (95% CI)
NTUH+TWB+Landseed	0.84 (0.75–0.93)
CGMH	0.81 (0.64–1.03)
SHH+TMU	0.87 (0.62–1.22)
**Combined**	**0.83 (0.76–0.92)**

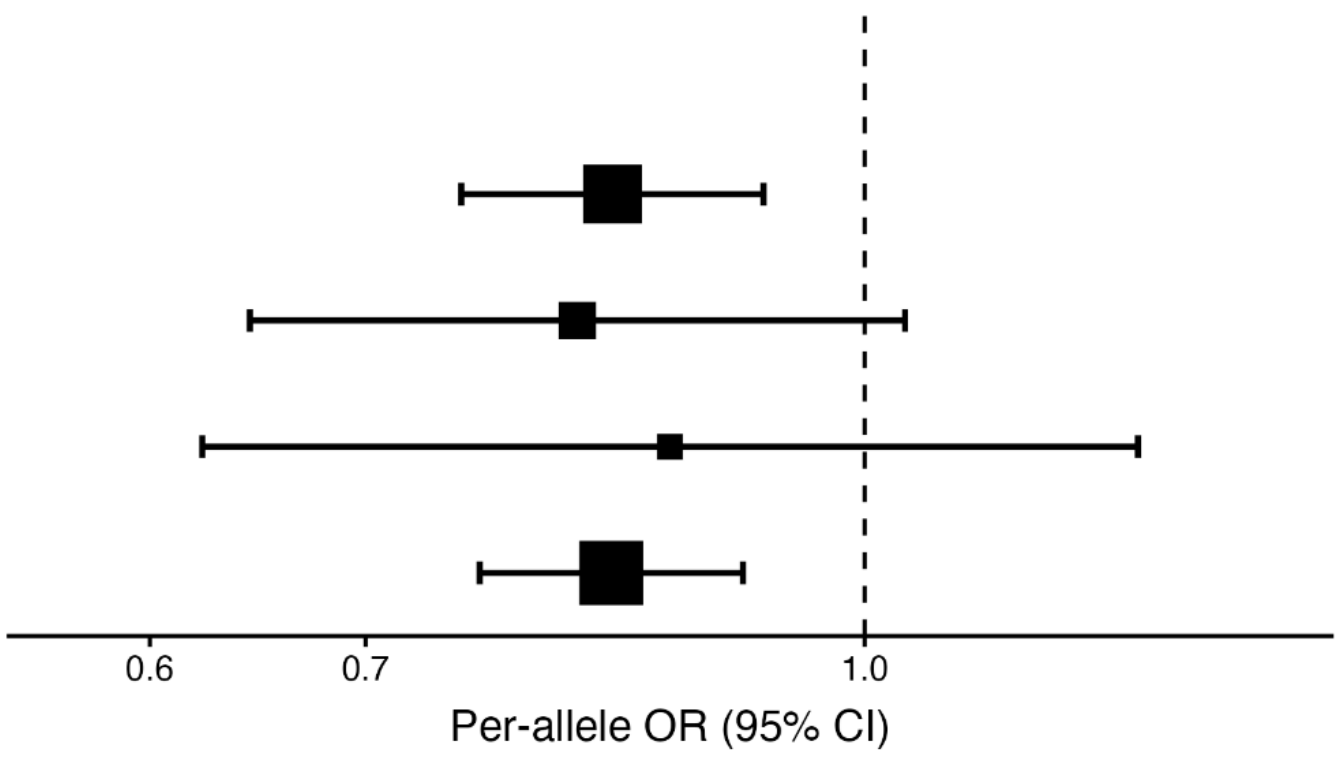

I² = 0.0%, P(heterogeneity) = 0.62

MCCC1, rs34822404

Cohort	OR (95% CI)
NTUH+TWB+Landseed	1.33 (1.20–1.47)
CGMH	1.28 (1.02–1.60)
SHH+TMU	1.32 (0.95–1.84)
**Combined**	**1.32 (1.21–1.45)**

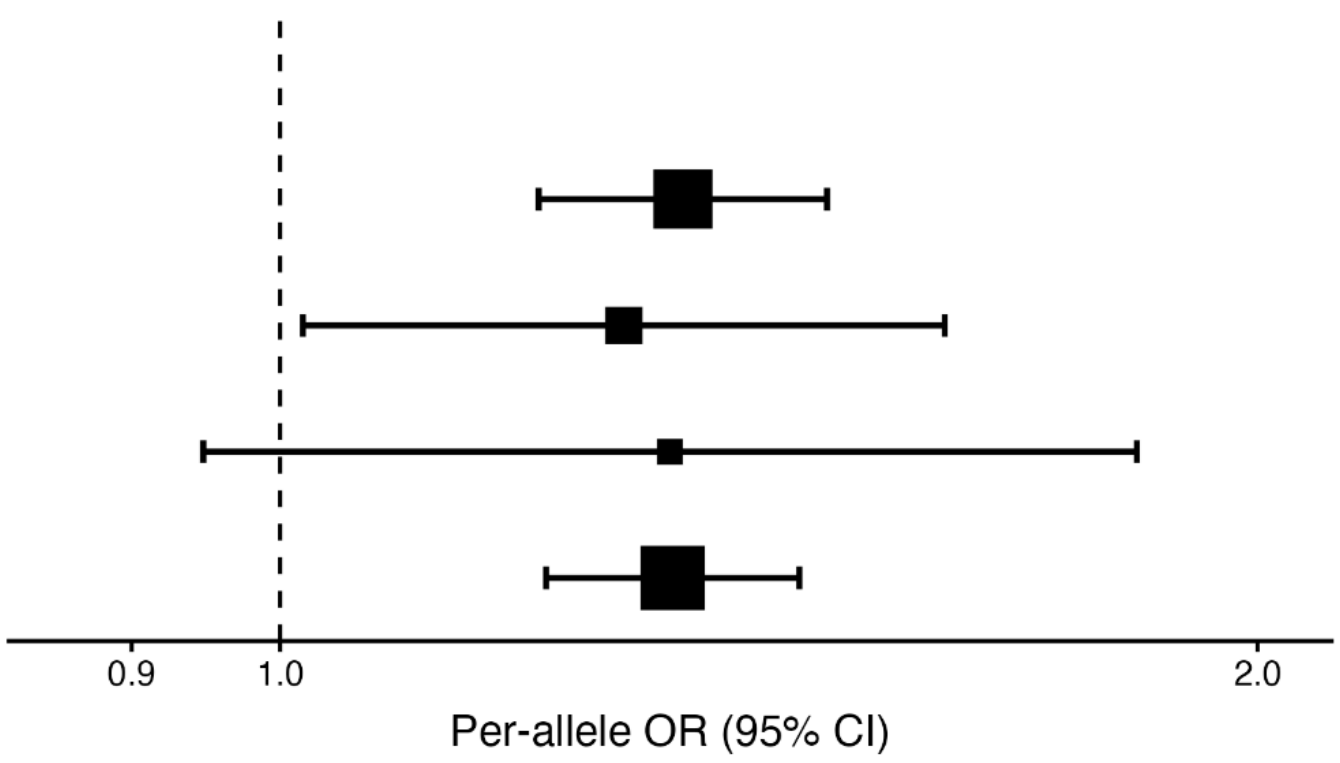

I² = 0.0%, P(heterogeneity) = 0.62

GCH1, rs3783640

Cohort	OR (95% CI)
NTUH+TWB+Landseed	1.27 (1.15–1.40)
CGMH	0.99 (0.79–1.24)
SHH+TMU	1.02 (0.75–1.38)
**Combined**	**1.20 (1.10–1.31)**

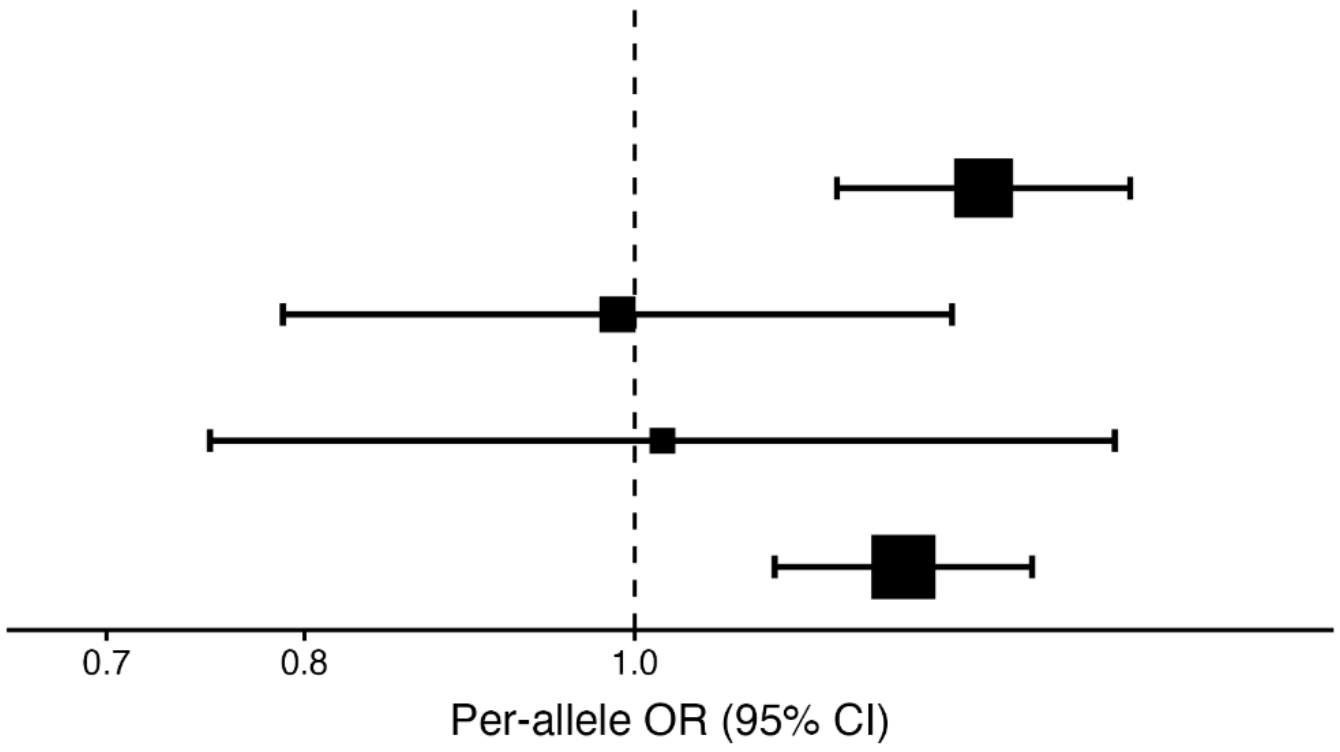

I² = 60.4%, P(heterogeneity) = 0.96

GALNT13, rs10186542

Cohort	OR (95% CI)
NTUH+TWB+Landseed	1.40 (1.20–1.64)
CGMH	1.29 (0.92–1.80)
SHH+TMU	1.68 (1.07–2.65)
**Combined**	**1.41 (1.23–1.61)**

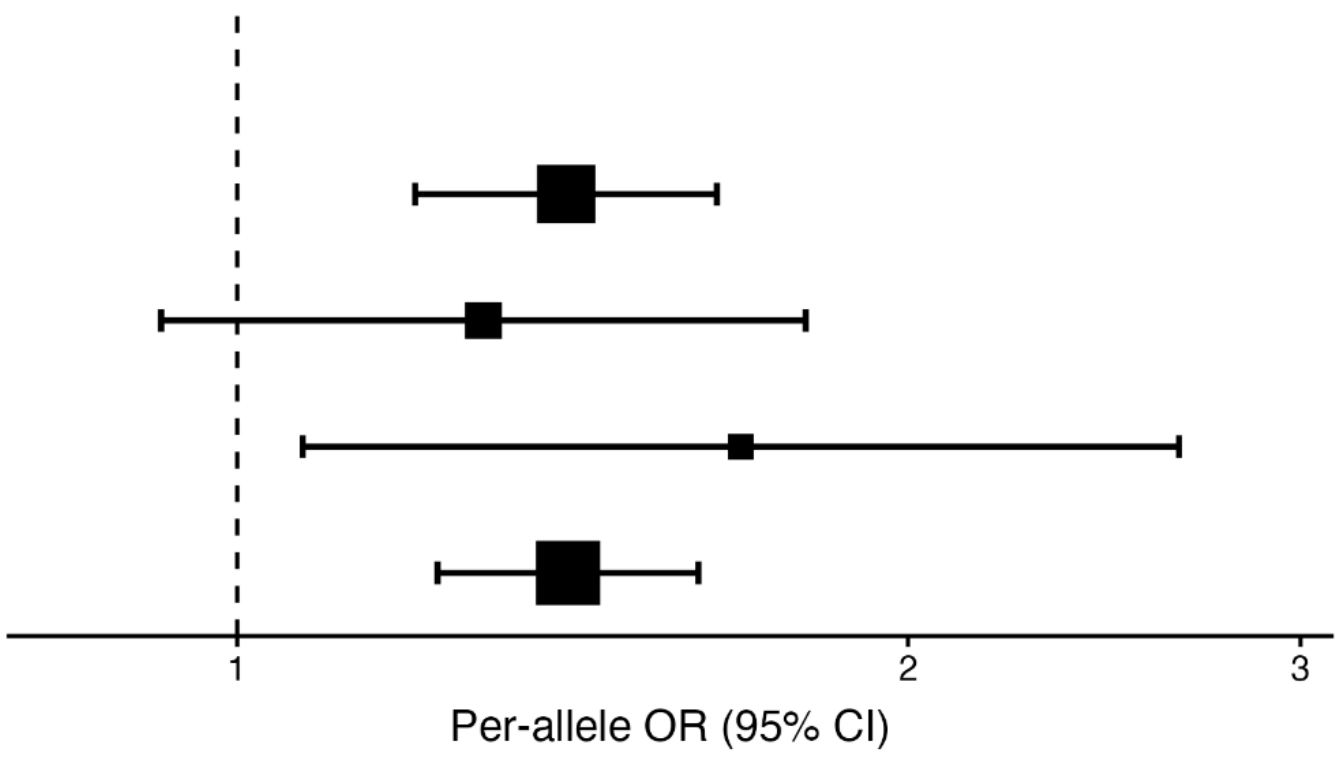

I² = 0.0%, P(heterogeneity) = 0.72

SCARB2/FAM47E, rs76591264

Cohort	OR (95% CI)
NTUH+TWB+Landseed	1.14 (1.02–1.27)
CGMH	1.24 (0.98–1.57)
SHH+TMU	1.61 (1.13–2.30)
**Combined**	**1.18 (1.08–1.30)**

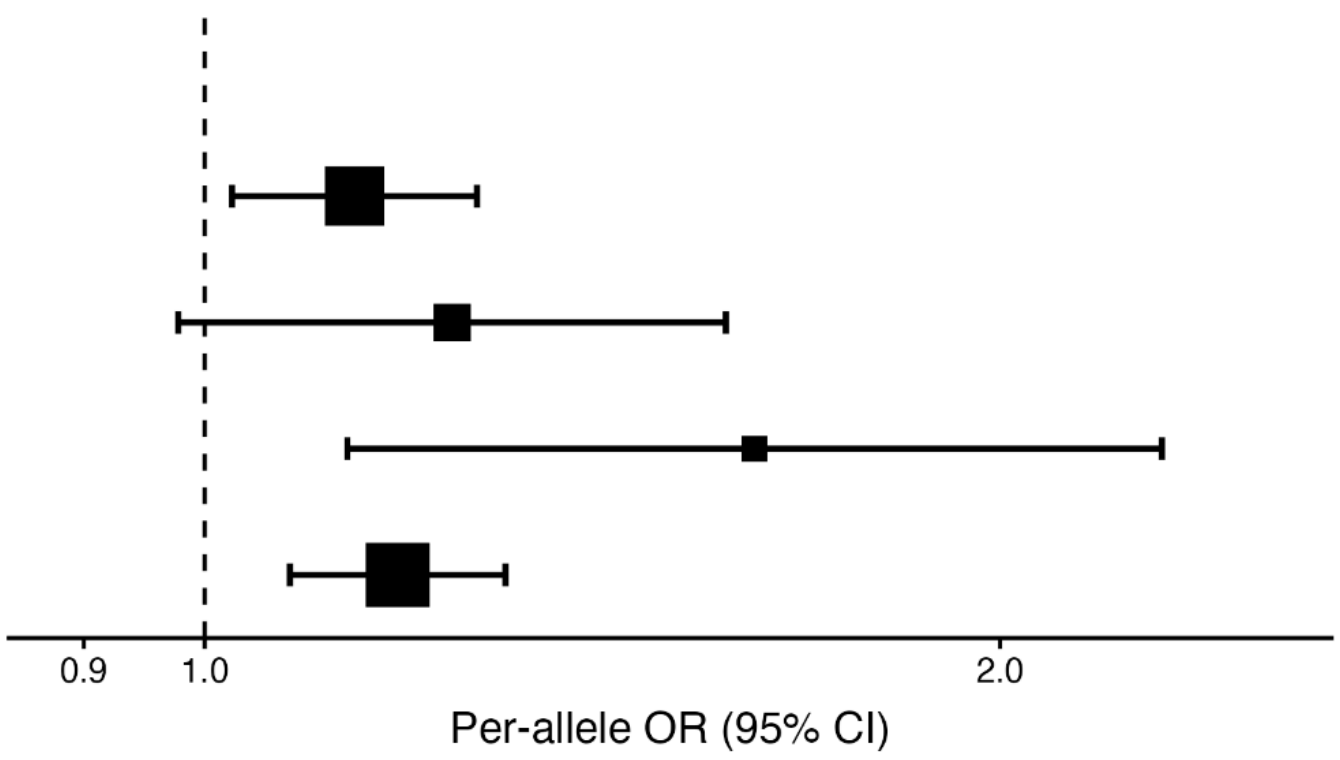

I² = 44.1%, P(heterogeneity) = 0.92

VPS13C, rs12900645

Cohort	OR (95% CI)
NTUH+TWB+Landseed	0.82 (0.74–0.91)
CGMH	0.80 (0.64–1.01)
SHH+TMU	1.13 (0.82–1.55)
**Combined**	**0.84 (0.76–0.92)**

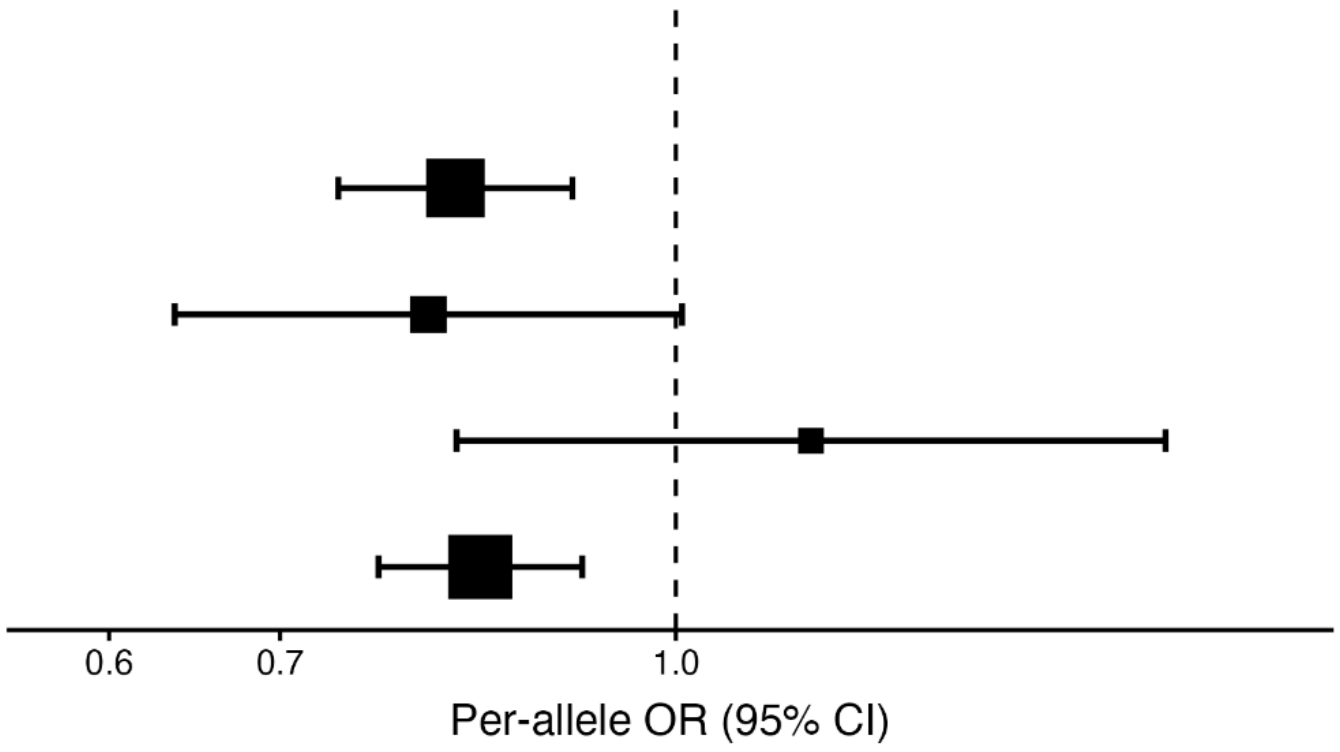

I^2 = 45.6%, P(heterogeneity) = 0.92

PPARGC1A, rs609501

Cohort	OR (95% CI)
NTUH+TWB+Landseed	1.18 (1.07–1.31)
CGMH	1.18 (0.93–1.48)
SHH+TMU	1.37 (0.98–1.92)
**Combined**	**1.20 (1.09–1.31)**

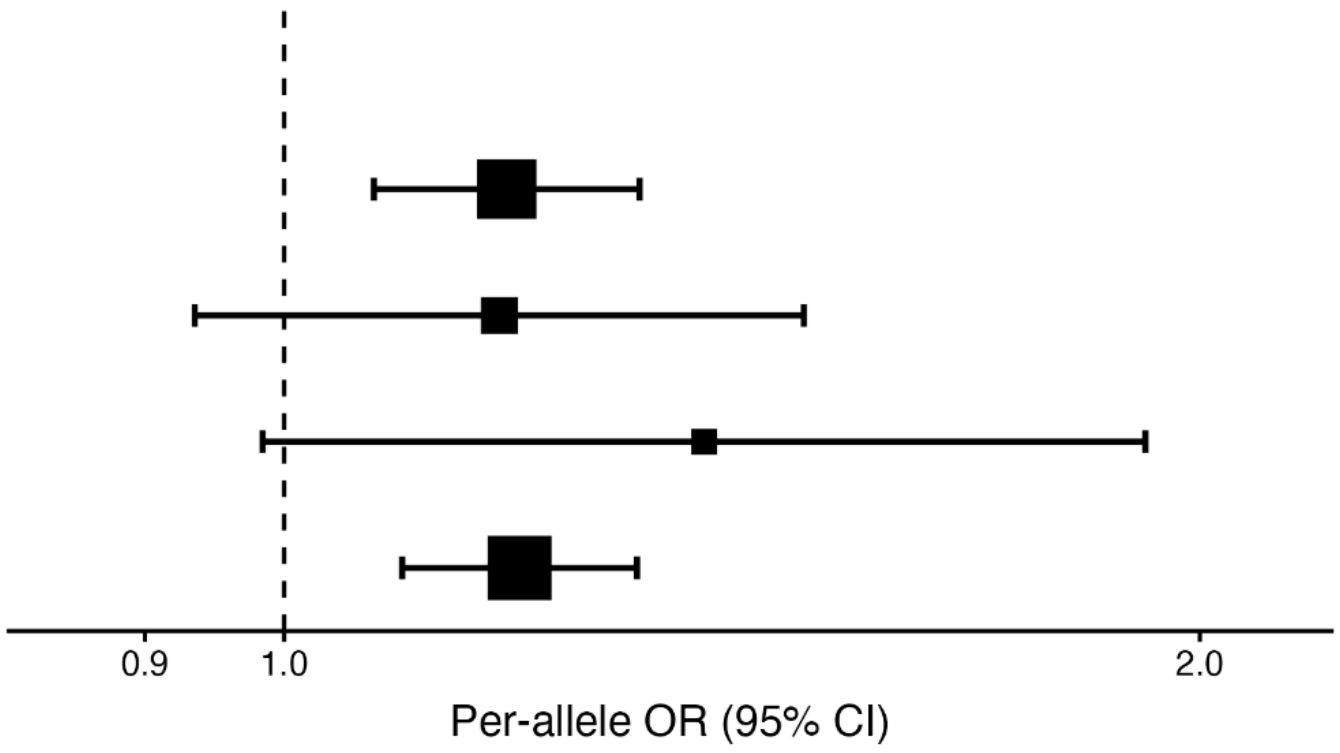

I^2 = 0.0%, P(heterogeneity) = 0.71

LRRK2 rs141336855

Cohort	OR (95% CI)
NTUH+TWB+Landseed	1.51 (1.14–2.01)
CGMH	1.81 (1.01–3.25)
SHH+TMU	1.58 (0.58–4.35)
**Combined**	**1.57 (1.22–2.01)**

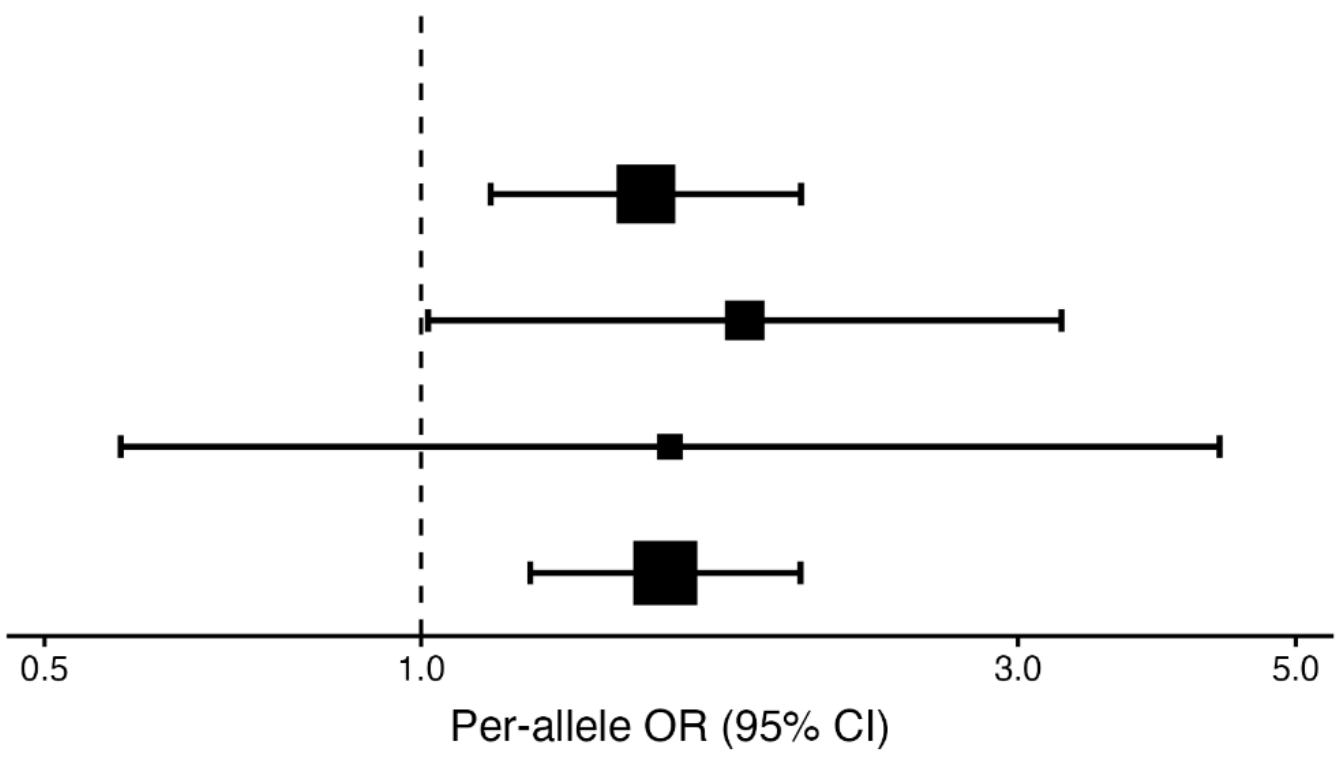

I^2 = 0.0%, P(heterogeneity) = 0.65

LRRK2 p.R1628P

Cohort	OR (95% CI)
NTUH+TWB+Landseed	1.34 (1.00–1.80)
CGMH	1.81 (0.91–3.59)
SHH+TMU	1.83 (0.73–4.59)
**Combined**	**1.44 (1.11–1.86)**

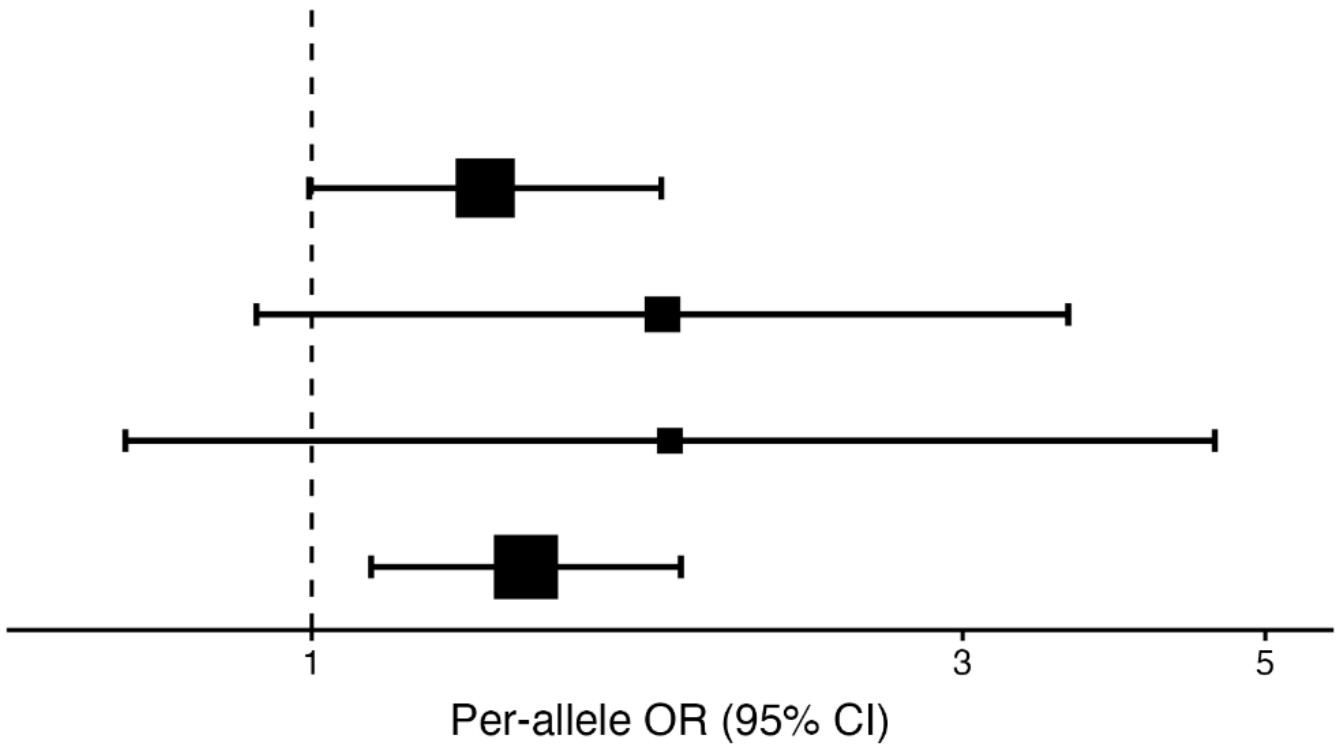

I^2 = 0.0%, P(heterogeneity) = 0.73

LRRK2 p.G2385R

Cohort	OR (95% CI)
NTUH+TWB+Landseed	1.58 (1.19–2.10)
CGMH	1.68 (0.92–3.07)
SHH+TMU	1.00 (0.34–2.92)
**Combined**	**1.56 (1.21–2.00)**

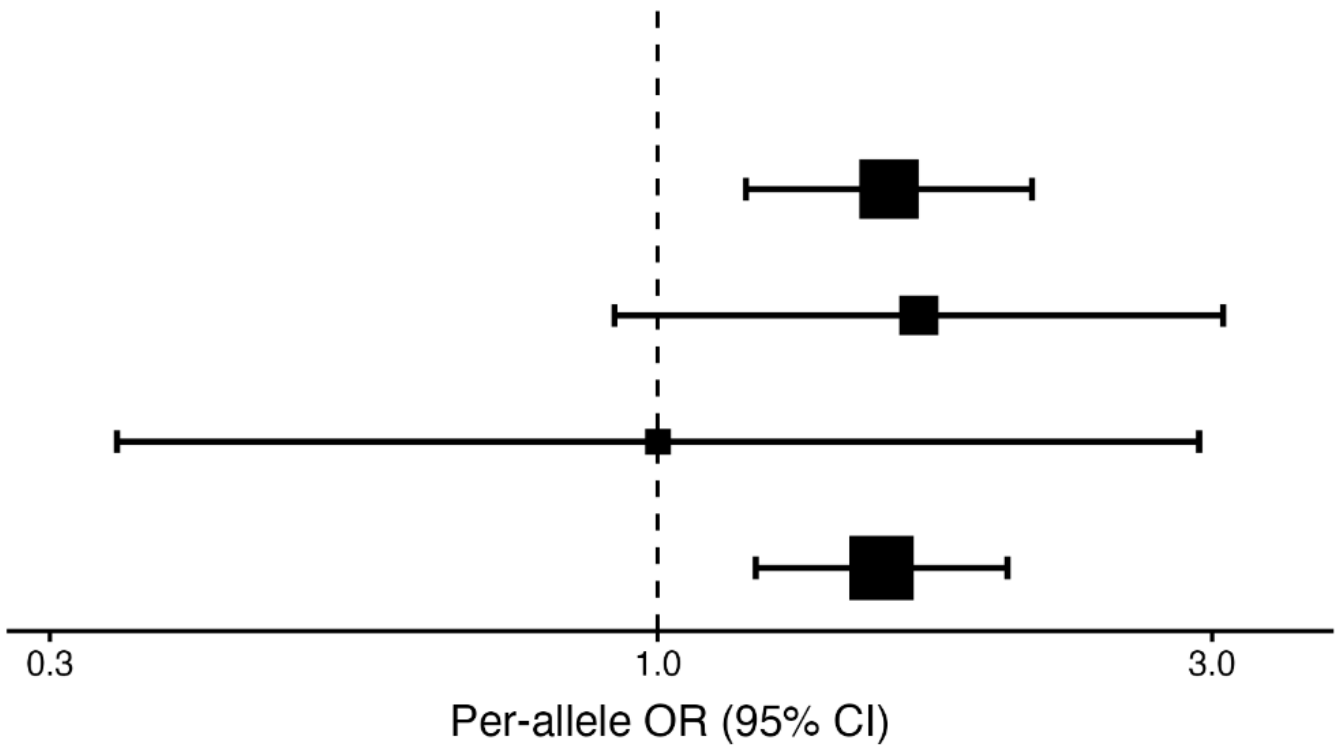

I^2 = 0.0%, P(heterogeneity) = 0.71

WWOX, rs9922439

Cohort	OR (95% CI)
NTUH+TWB+Landseed	1.24 (1.12–1.38)
CGMH	1.21 (0.96–1.52)
SHH+TMU	1.38 (1.00–1.91)
**Combined**	**1.25 (1.14–1.36)**

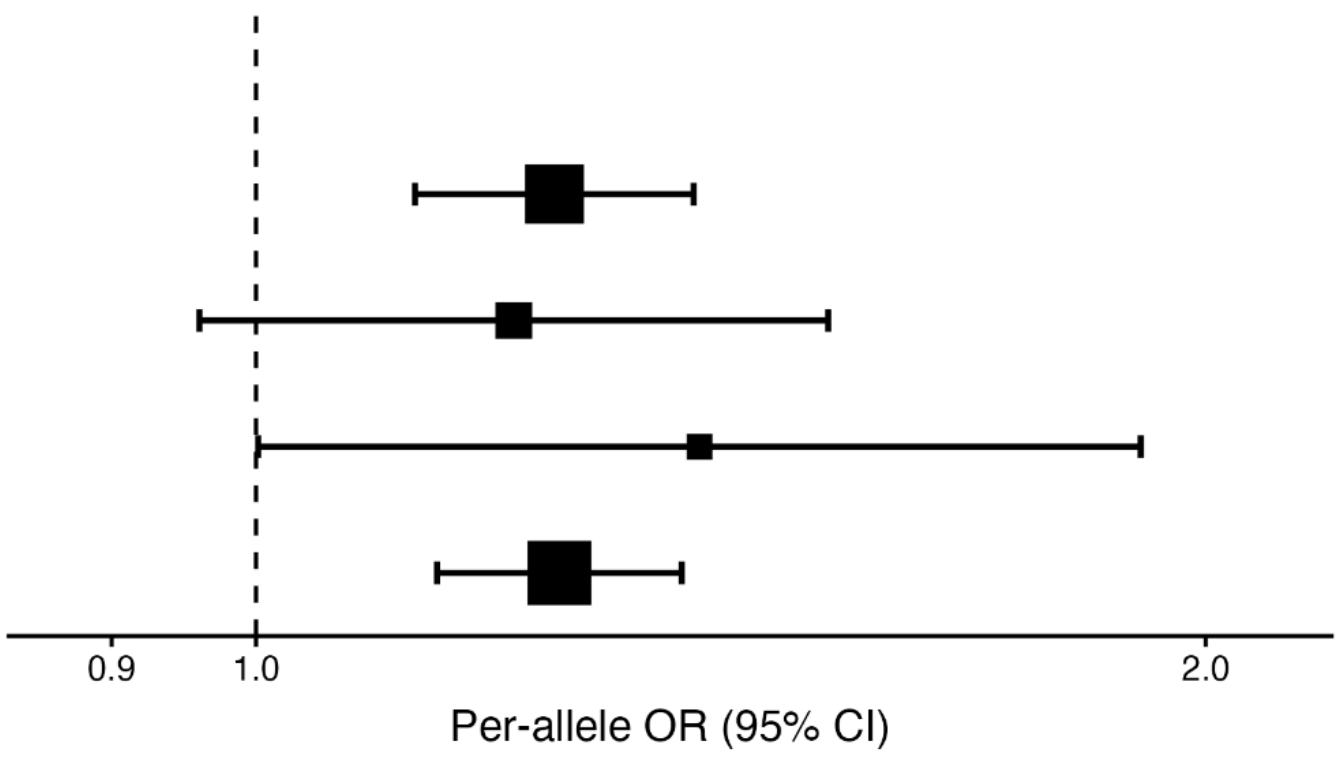

I^2 = 0.0%, P(heterogeneity) = 0.67

Supplementary Figure S6. Haplotype structure and association of *GCH1* in the Taiwanese Parkinson's disease cohort.

(a) Lower-triangle LD heatmap among rs841, rs11158026, and rs3783640 (hg38), calculated in cohort controls; the Taiwan-specific rs3783640 is in LD with rs11158026 (Nalls et al.), and both are in partial LD with rs841 ($r^2 = 0.31$). (b) Haplotype composition and frequencies (displayed for haplotypes with control frequency >1%), ordered by control frequency. (c) Forest plot of haplotype associations with PD (OR, 95% CI). Haplotypes carrying rs3783640-C and rs11158026-T show a protective effect against PD, whereas rs841 alone is not significantly associated.

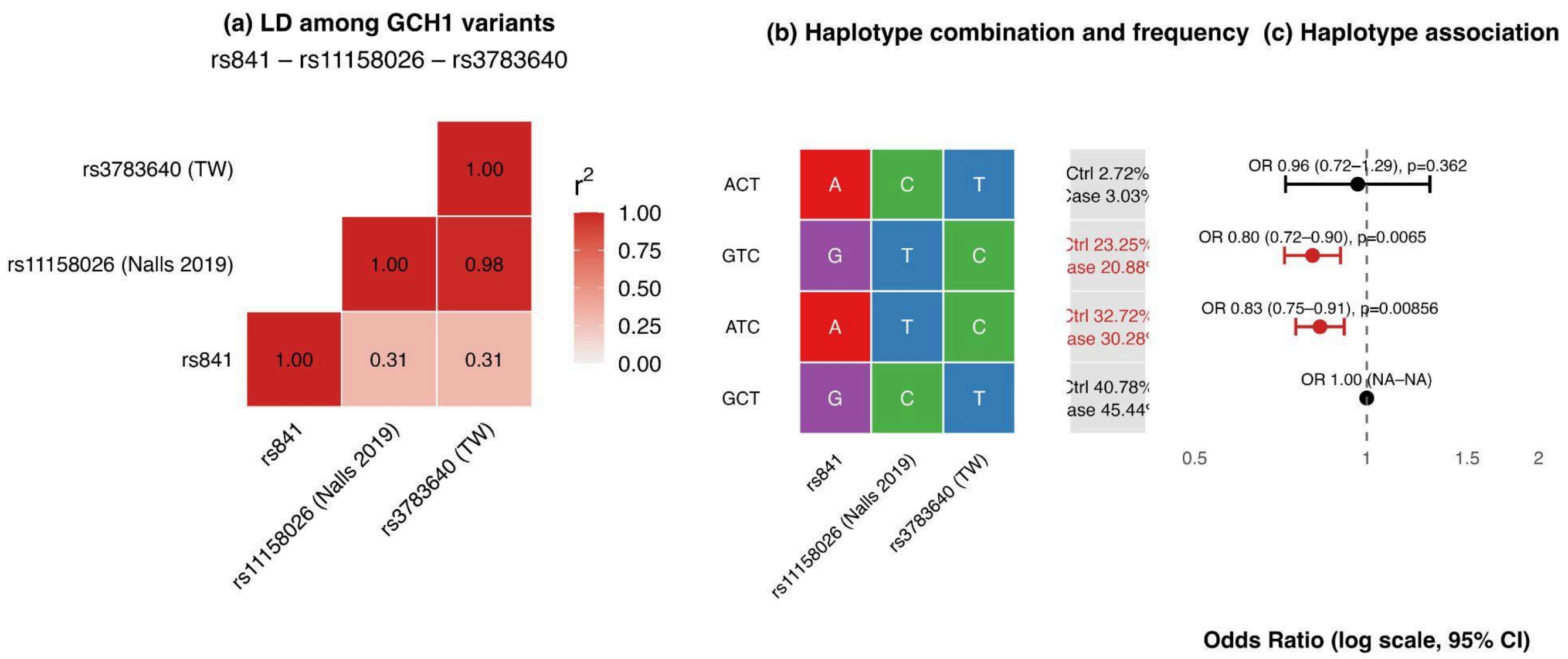

Supplementary Figure S7. Performance of externally derived PRS models in the Taiwan cohort.

We evaluated two PRS models constructed using *externally published* variant lists and effect sizes: (i) **PRS-EUR**, based on the EUR meta-analysis (Nalls et al.), and (ii) **PRS-EUR-Asia**, which augments PRS-EUR with 11 independent East Asian lead variants from the pan–East Asian GWAS (Foo et al., 2020). In our cohort, **PRS-EUR** achieved an AUC of **0.590** (95% CI, **0.574–0.607**). Adding the Asian variants modestly improved discrimination, with **PRS-EUR-Asia** reaching an AUC of **0.606** (95% CI, **0.590–0.623**).

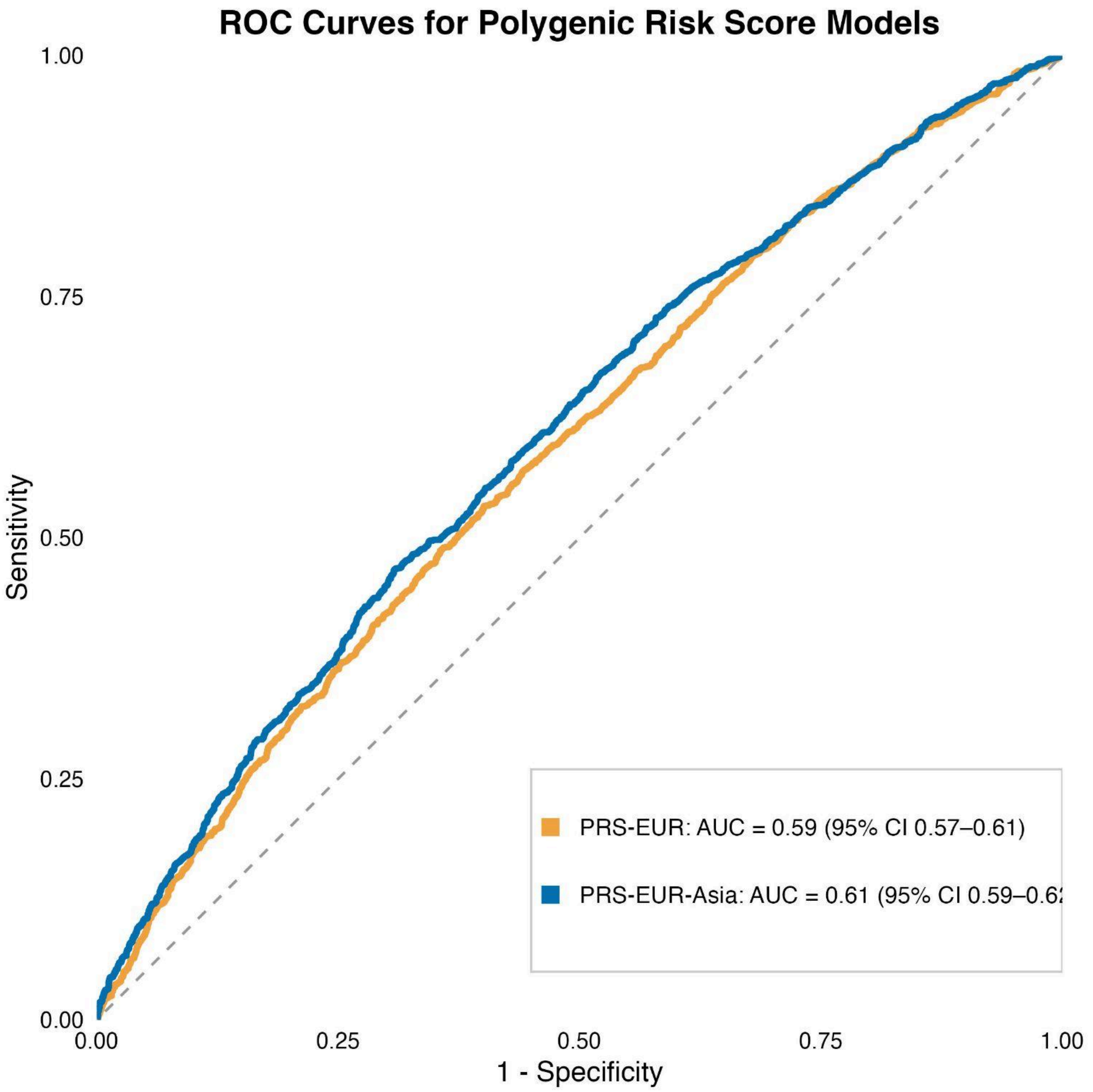

Supplement: Supplementary file 1 [file 41531_2026_1349_MOESM1_ESM.pdf]
